# Enantiodiscrimination of Inherently Chiral Thiacalixarenes by Residual Dipolar Couplings

**DOI:** 10.1021/acs.joc.2c02594

**Published:** 2023-01-19

**Authors:** Markéta Tichotová, Tomáš Landovský, Jan Lang, Sharon Jeziorowski, Volker Schmidts, Michal Kohout, Martin Babor, Pavel Lhoták, Christina M. Thiele, Hana Dvořáková

**Affiliations:** † Laboratory of NMR Spectroscopy, University of Chemistry and Technology Prague (UCTP), Technická 5, 166 28Prague 6, Czech Republic; ‡ Department of Physical and Macromolecular Chemistry, Charles University, Hlavova 8, 128 00Prague 2, Czech Republic; § Institute of Organic Chemistry and Biochemistry, Czech Academy of Sciences, Flemingovo náměstí 542, 160 00Prague 6, Czech Republic; ∥ Department of Organic Chemistry, University of Chemistry and Technology Prague (UCTP), Technická 5, 166 28Prague 6, Czech Republic; ⊥ Department of Chemistry, Technical University of Darmstadt, Alarich-Weiss-Straße 16, 64287Darmstadt, Germany; # Department of Solid State Chemistry, University of Chemistry and Technology Prague (UCTP), Technická 5, 166 28Prague 6, Czech Republic; ∇ Faculty of Mathematics and Physics, Charles University, Ke Karlovu 3, 121 16Prague 2, Czech Republic

## Abstract

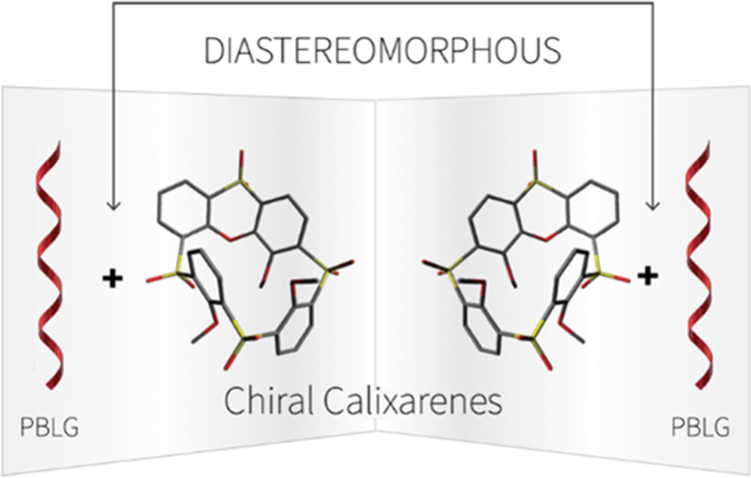

Inherently chiral compounds, such as calixarenes, are chiral due to a nonplanar three-dimensional (3D) structure. Determining their conformation is essential to understand their properties, with nuclear magnetic resonance (NMR) spectroscopy being one applicable method. Using alignment media to measure residual dipolar couplings (RDCs) to obtain structural information is advantageous when classical NMR parameters like the nuclear Overhauser effect (NOE) or *J*-couplings fail. Besides providing more accurate structural information, the alignment media can induce different orientations of enantiomers. In this study, we examined the ability of polyglutamates with different side-chain moieties—poly-γ-benzyl-l-glutamate (PBLG) and poly-γ-*p*-biphenylmethyl-l-glutamate (PBPMLG) —to enantiodifferentiate the inherently chiral phenoxathiin-based thiacalix[4]arenes. Both media, in combination with two solvents, allowed for enantiodiscrimination, which was, to the best of our knowledge, proved for the first time on inherently chiral compounds. Moreover, using the experimental RDCs, we investigated the calix[4]arenes conformational preferences in solution, quantitatively analyzed the differences in the alignment of the enantiomers, and discussed the pitfalls of the use of the RDC analysis.

## Introduction

Chirality plays an important role in biochemistry, as the vital molecular components, such as proteins and nucleic acids, are chiral. Enantiomers of a certain structure can interact differently with the target biomolecules. Thus, in the enantioselective synthesis of new drugs, there is a permanent interest in the development of methods for distinguishing the enantiomers and in the determination of enantiomeric purity. NMR spectroscopy provides several ways how to discriminate the enantiomers, e.g., shift reagents or the Mosher method. The principal of these methods is the interactions of the individual enantiomers with the chiral environment generating diastereomeric pairs that are distinguishable in NMR spectra (see below for a more detailed explanation).

To provide an alternative in the case when the above-mentioned isotropic methods fail, measurements of residual dipolar couplings (RDCs) can be applied. The RDC method has been widely used for structural studies of biological macromolecules and also the small molecules in the last decade (for review, see refs (1−3)) as it provides complementary information to the conventional NOE distance^4^ or to the dihedral angles from the vicinal couplings ^3^*J*.^5^ However, RDCs usually cannot be observed in an ordinary liquid solution, where molecular tumbling is isotropic, and thus the dipolar splitting is averaged to zero. To observe these parameters, the analyte must be partially oriented with respect to the external magnetic field using a suitable alignment medium so that a small part of the full dipolar coupling (usually comparable with the size of a scalar coupling) is recovered. For the partial orientation of small organic molecules, organic-solvent-compatible alignment media such as stretched/compressed polymer gels and lyotropic liquid crystalline (LLC) solutions have been designed (for review, see refs (1, 6)). Out of the class of LLC media, homopolypeptides like poly-γ-benzyl-l-glutamate (PBLG)^7^ and poly-γ-ethyl-l-glutamate (PELG)^8^ as well as oligopeptides,^9,10^ polyguanidines,^11^ polyisocyanides,^12^ and polyacetylenes^13,14^ have been widely used. Previously, we have successfully utilized PBLG and PELG for conformational analysis of intramolecular *meta*-bridged calix[4]arenes^15−18^ and for cyanomethyl-substituted phenoxathiin-based thiacalix[4]arene.^19^

**Figure 1:**
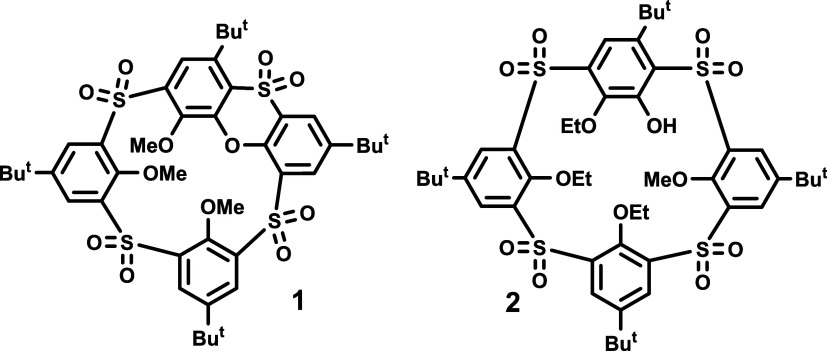
Studied compounds **1** and **2**. Compound **1** is an example of a “closed” phenoxantiin-based structure and compound **2** is an example of an alkylated “open” structure.

We have recently developed a synthetic methodology to produce phenoxathiin-based thiacalix[4]arene derivatives.^20−22^ Compound **1**(21) is an example of a macrocycle bearing a fused heterocyclic moiety that imparts considerable rigidity to the system. On the other hand, compound **2** displays more structural flexibility (Figure 1) due to the cleavage of the oxygen bridge. In contrast to classical calix[4]arenes which can adopt four conformations (called the *cone*, *partial cone* (*PaCo*), *1,2-alternate* (*1,2-alt*), and *1,3-alternate* (*1,3-alt*)),^23^ the structures of the phenoxathiin-based thiacalix[4]arenes **1** and **2** enable the formation of other conformations. In the case of **1**, four possible conformations can arise: *1,2-alt*, *cone*, *PaCoC*, and *PaCoD* (*PaCo* with ring C or D inverted, respectively). On the other hand, compound **2**, possessing structural features of both calixarenes and pillararenes^24^ (*meta* and *para* bridges), can afford eight conformations: *1,2-altAD* (rings A and D inverted), *1,2-altAB* (rings A and B inverted), *1,3-alt*, *cone*, *PaCoA*, *PaCoB*, *PaCoC*, and *PaCoD*.

**Figure 2:**
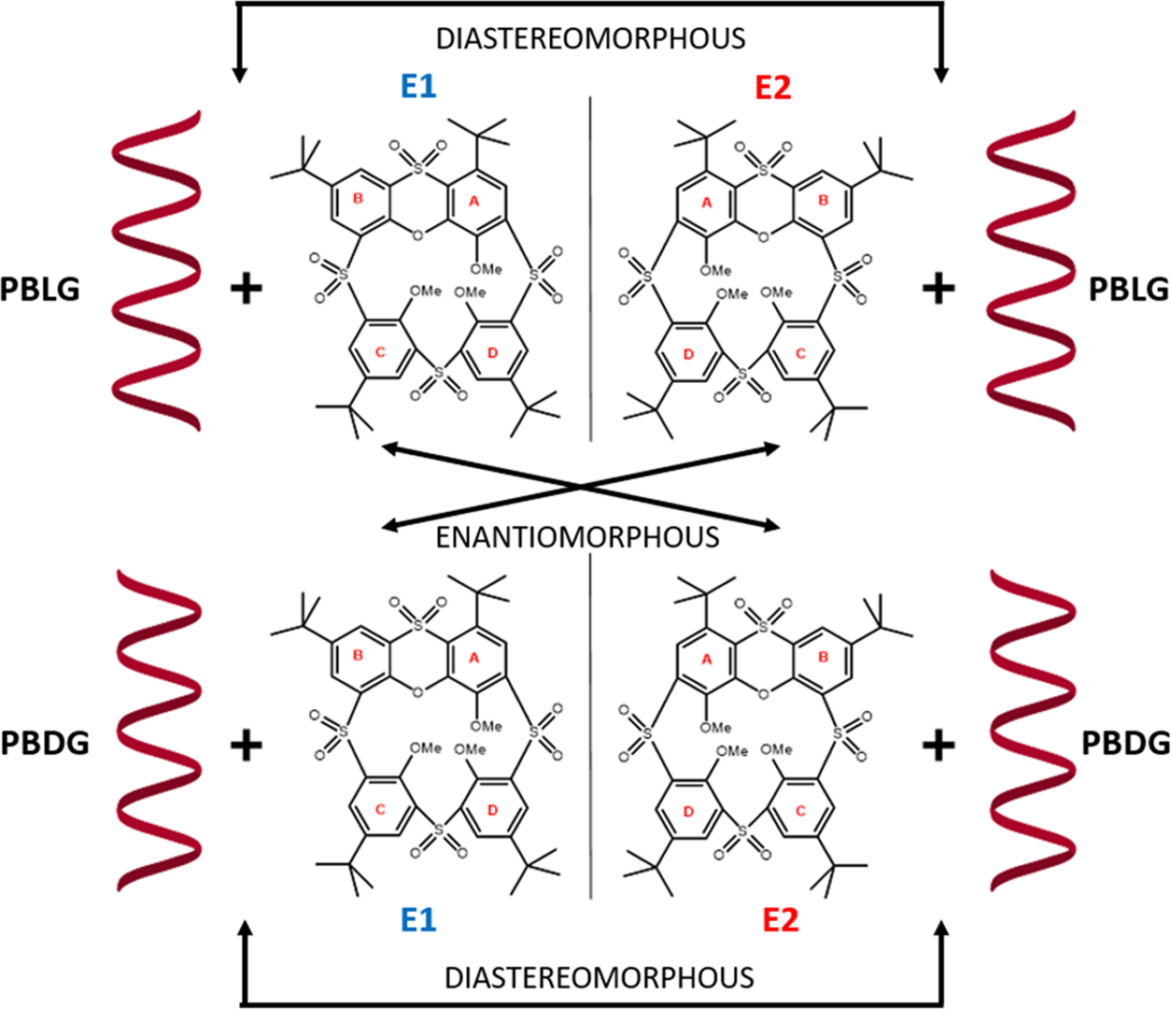
Possible stereochemical relations that need to be considered when combining both inherently chiral enantiomers of **1** (E1 and E2) with the racemic mixture of PBLG and PBDG (poly-γ-benzyl-l- and d-glutamates).

Similar to classical calix[4]arenes, the new types of cavities have potential in the design of various receptors and more complex supramolecular systems. Moreover, these types of calixarenes are inherently chiral, which brings other promising applications in diverse areas of chemistry. The asymmetry in inherent chirality arises from the nonplanar structure in 3D space, in contrast to the chirality caused by the stereogenic chiral center. Inherent chirality^25−27^ of compounds **1** and **2** is an excellent opportunity to probe whether their interaction with chiral alignment media leads to enantiodiscrimination. Enantiomers undergo diastereomorphous interactions with chiral alignment media. As a result, they orient differently and may become distinguishable in NMR experiments (Figure 2). This concept was first used by the group of Lesot and Courtieu, who showed two sets of detectable signals in NMR spectra, including ^2^H NMR.^28−32^ Since then, a number of examples of chiral discrimination of compounds with central chirality, such as (±)-isopinocampheol, (±)-β-pinene, etc., have been published (refs (14, 33−38)). Most frequently, polypeptide- and polyacetylene-based alignment media with varying side chains have been employed for enantiodiscrimination due to their helical secondary structure and the variability of the polymer (and the LLC phase) properties arising from the side-chain substitution. Recently, new types of alignment media (e.g., biphenyl-containing alignment medium PBPMLG) were prepared to examine the influence of a chiral side chain on enantiodiscrimination,^39,40^ to use their thermoresponsive properties,^41−43^ or to control their molecular weight.^44^

We decided to examine whether the reported enantiodiscriminating properties of PBLG and PBPMLG^43^ in both CDCl_3_ and THF-*d*_8_ as solvents can be applied to our calixarene-based inherently chiral systems. To the best of our knowledge, so far, there are only a few examples of discrimination of molecules lacking stereogenic centers, e.g., helically chiral tris (diamine)ruthenium(II) enantiomers,^45^ nonamethoxycyclotriveratrylene,^46^ and axially chiral biaryls^47^ using PBLG as an alignment medium.

## Results and Discussion

**Scheme 1:**
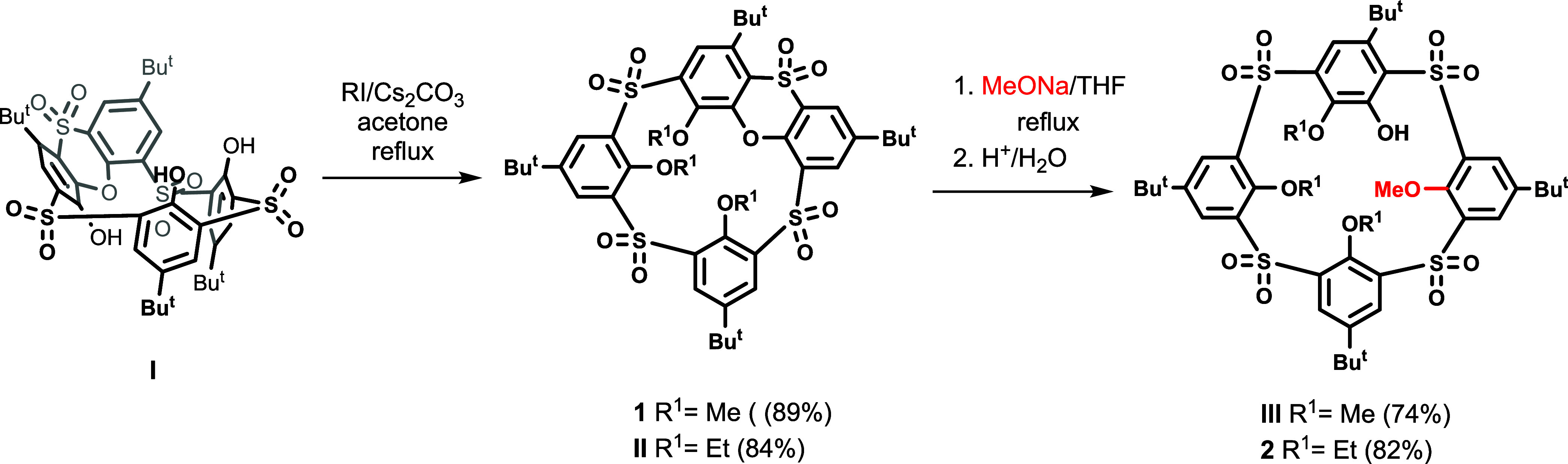
Synthesis of the Compounds Studied

Compounds used for this study were obtained following a recently developed synthetic methodology leading to the phenoxathiin-based thiacalix[4]arene derivatives.^20−22^ As shown in Scheme 1, starting tetrasulfone **I** was alkylated by the corresponding alkyl iodides in the presence of Cs_2_CO_3_ to provide the peralkylated derivatives **1** and **II** in very good yields (84–89%). As recently reported, the reaction of **1** with sodium methoxide in THF led smoothly to the cleaved product **III** in 74% yield.^48^ To evaluate the influence of substituents on the conformational behavior and mobility of the system, the same reaction was carried out with **II** bearing Et groups. The analogue **2** was isolated in 82% yields after column chromatography on a silica gel.

**Figure 3:**
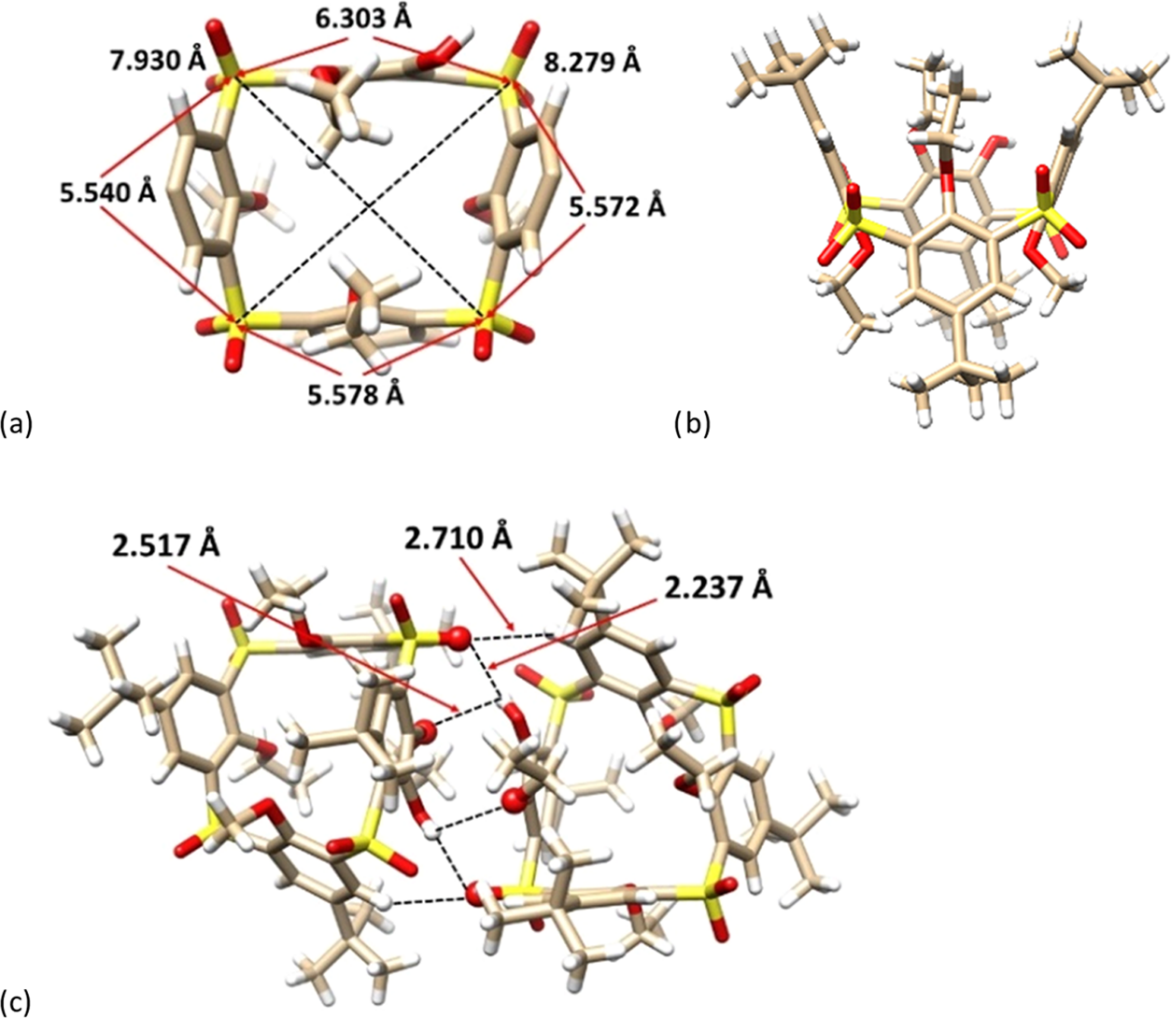
Single-crystal X-ray structures of compound **2** (racemic): (a) top-view (the *tert*-butyl groups removed for better clarity); (b) side-view; and (c) dimeric motif of **2** showing HB interactions between the enantiomers.

The single-crystal X-ray study of **2** provided the final unequivocal structural evidence. Compound **2** adopts the monoclinic system, space group *P*2_1/*c*_. Using the common nomenclature of calixarene systems (despite the fact that one aromatic unit is *para*-substituted), the molecule adopts the *1,3-alternate* conformation with alternating up and down mutual arrangement of the phenolic oxygens. Contrary to common thiacalix[4]arenes possessing a regular square shape for the *1,3-alternate* conformation, the cavity of **2** resembles a trapezoid due to the presence of the *para*-substituted phenolic unit. Consequently, one side is much longer (6.303 Å) than the others (5.572, 5.578, and 5.540 Å), with the main diagonals of 8.279 and 7.930 Å, measured as a distance between two opposite sulfur atoms (Figure 3a). If we define the main plane of the molecule by the four sulfur bridges, the corresponding interplanar angles Φ are 77.57, 108.84, 80.16, and 112.43° starting from *para*-bridged moiety and continuing clockwise (see Figure 3b).

The crystal contains both enantiomers in an equal ratio, which are connected via a net of hydrogen bonds, thus forming a dimeric motif within the crystal packing. As shown in Figure 3c, the free OH group from one molecule interacts with two oxygens on the opposite enantiomer (S=O and OEt), forming two HBs. The corresponding lengths (2.237 Å for the O–H···O=S interaction and 2.517 for the O–H···OEt bond) of these HBs indicate strong interactions in the solid state. The whole arrangement is completed by the close contact between one of the sulfoxide oxygens and the aromatic hydrogen atom on the opposite molecule (S=O···H–Ar = 2.710 Å).

**Figure 4:**
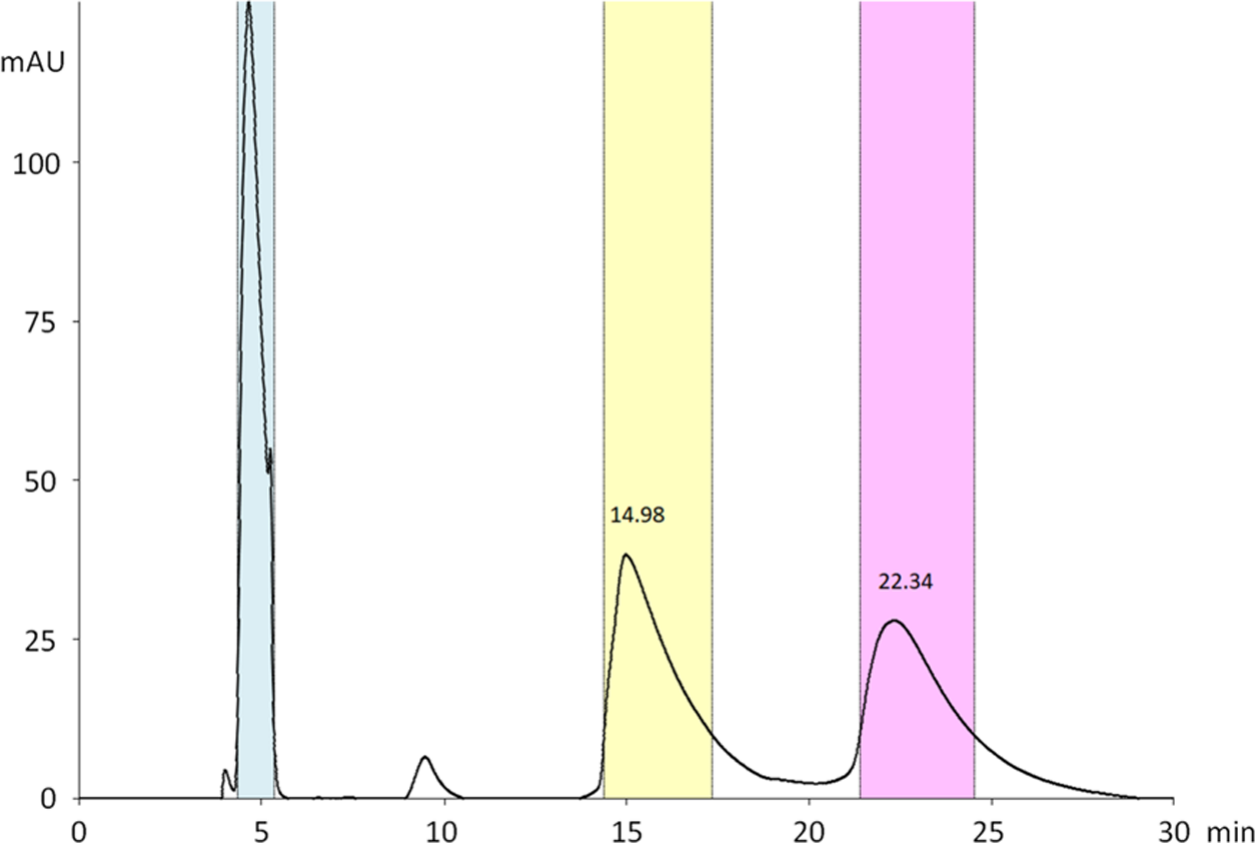
Chromatogram of the preparative chiral separation of **2** using Chiralpak IA (250 × 20 mm^2^ i.d., 5 μm) with the heptane/propan-2-ol (96/4, v/v) mixture as an eluent. The blue marked peak and the peak with a retention time of about 10 min were separated but not further analyzed.

Chiral resolution of compounds was performed using a polysaccharide-based analytical column Chiral Art Amylose-SA (250 × 4.6 mm^2^ i.d., 5 μm) (YMC, Germany). After finding the best possible conditions for each substance, a chiral preparative column, Chiralpak IA (250 × 20 mm^2^ i.d., 5 μm) from Daicel (Japan), was employed to perform their chiral separation on the multi-milligram scale. While the separation of compounds **II** and **III** did not provide fully separated enantiomers, compounds **1** and **2** were successfully resolved (Figure 4).

### Alignment of the Racemates of **1** and **2**

**Figure 5:**
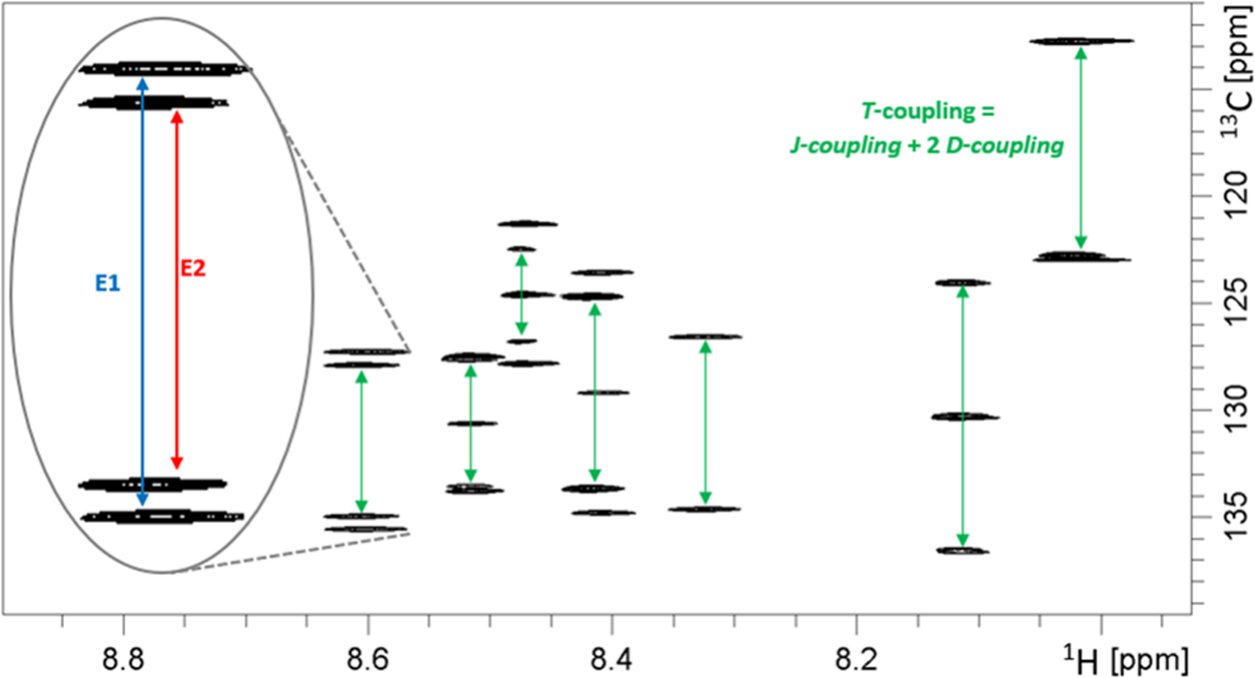
F1-coupled HSQC (aromatic part) of the anisotropic sample of compound **1** (PBLG, THF-*d*_8_) with visible splitting for two enantiomers, **E1** and **E2**. The splitting in the ^13^C dimension corresponds to the sum of the individual contributions to the ^13^C–^1^H coupling from the scalar (*J*) and dipolar (*D*) coupling, with total coupling *T* = *J* + 2*D*. A scaling factor of 8 has been used in the indirect dimension.

The racemates of **1** and **2** were measured in four combinations of PBLG or PBPMLG media and CDCl_3_ or THF-*d*_8_ solvents, as all of these LLC phases were reported to have enantiodiscriminating properties.^36,37,39,40^ The enantiodiscrimination was clearly demonstrated by the appearance of two separate doublets in the F1-coupled HSQC spectra in all acquired combinations of both **1** and **2** (see an example of **1** in Figure 5).

For **1**, we obtained seven or eight ^1^*D*_CH_ values for both enantiomers (**E1** and **E2**) (see Tables S5–S13); two values corresponding to the rotating *t*-butyl groups were converted to one-bond C–C ^1^*D*_*CC*_—the RDC between methyl and tertiary carbon nuclei.^49^ The RDC@hotFCHT software^50^ fitting procedure undoubtedly confirmed *PaCoD* as the best-fitting structure for both enantiomers E1 and E2 (Table 1), which is in accordance with the X-ray structure.^21^

**Table 1:** Weighted Quality Factors *q* for Racemic Mixtures (**E1** + **E2**) of Compound **1** in PBLG or PBPMLG with CDCl_3_ or THF-*d*_8_

	PBLG/CDCl_3_	PBLG/THF-*d*_8_	PBPMLG/CDCl_3_	PBPMLG/THF-*d*_8_
**E1**	0.0999^a^	0.1266	0.1120	0.0143
**E2**	0.0643	0.0672	0.0491	0.0295

a Lower value of the *q* factor indicates a better fit of the experimental RDCs to the best-fitting *PaCoD* structural model.

In the case of **2**, the differential splitting was observed for seven or eight cross-peaks. However, the enantiomer splitting was less distinctive. As a result, the values of the extracted RDCs were less accurate, giving an unsatisfactory fit to the structural models, as quantified by *q,*(51) an uncertainty-weighted quality factor. The derived values of *q* were either much larger than those observed for the good fit of compound **1** or, in the case of the PBLG/CDCl_3_ system, all conformations gave *q* < 0.1, essentially preventing an assignment of the best-fitting structure (see Tables S5 and S14–S19). Interestingly, we were not able to obtain any RDC data in the PBPMLG/CDCl_3_ system for compound **2**. The unsatisfactory results in the case of compound **2** can be explained by its much lower rigidity compared to macrocycle **1**, possessing the phenoxathiin system. A higher degree of freedom of the individual rings then leads to smaller differences in the orientations of the aromatic subunits within the given conformation, which is reflected in a lower agreement between the experiment and the theory.

Thus, although qualitatively, the presence of both enantiomers is confirmed for both **1** and **2**, there are sometimes complications with the quantitative analysis of RDC splitting in the racemic mixtures. The alignment tensor calculation is an important component of the RDC analysis since it enables a consistency check of the experimental data as well as the assumed geometry of the molecule. Therefore, there are several points that should be carefully examined during the data analysis:

(i) To prove that the observed two sets of signals are the result of the occurrence of different enantiomers, not, e.g., conformers. To solve this, we mixed the racemate of the studied compounds with the racemic mixture of poly-γ-benzyl-glutamate (PBLG and PBDG) (see Alignment of Racemates **1** and **2** with the Racemate of PBLG/PBDG).
(ii) To examine whether the pairs of coupled signals were identified correctly. The problem is to assign the obtained RDCs to the individual enantiomers. We have a total number of four components for two doublets (see, e.g., Figure 5) of a particular ^1^*D*_CH_ RDC. A first guess is that the outer pair and the inner pair belong to each enantiomer. Nevertheless, care must be taken as the centers of the doublet may be shifted due to residual chemical shift anisotropy (RCSA). It is not generally granted that the movements of doublet centers lead to a completely general situation for the ordering of the components of a given signal/C–H bond. However, the pulse sequence employed in this work^52^ uses a variable scaling factor for the coupling resulting in an overall splitting of ∼1 kHz for the scaling factor of 8 employed here. The observed RCSA is usually very small in these weakly aligned cases—usually, less than a few Hz, such that these effects can be discriminated safely. Therefore, in our spectra, the inner and outer components belong to the same C–H coupling in all cases. More importantly, there is no apparent simple way to assign the two measured RDC splittings to the enantiomers. Statistically, there are 2*^n^* (*n* is the number of the enantiomer-resolved RDCs) options for the assignment. We performed alignment tensor calculations for all possible combinations of the obtained RDCs to determine the correct combination according to *q* factors. In many cases, there are, however, several solutions with similar *q* factors. Thus, to facilitate an unambiguous assignment, we chromatographically separated the enantiomers and then compared the data with that of the racemic mixture (see Alignment of the Separated Enantiomers **1-E1**, **1-E2** and **2-E1**, **2-E2** with PBLG/THF-*d*_8_).
(iii) To check whether the conformations of the phenoxathiin-based thiacalix[4]arenes **1** and **2** have been represented correctly in the DFT structures. A wrong conformation would be indicated by a bad fit. To rule out any issues with the DFT structures, we fit our experimental data also to the X-ray structures (see Alignment of the Separated Enantiomers **1-E1**, **1-E2** and **2-E1**, **2-E2** with PBLG/THF-*d*_8_).
(iv) To prepare the anisotropic sample with the appropriate degree of alignment. It is sometimes not possible to distinguish the two doublets if one of the cross-peaks of the two respective enantiomers is broadened and, e.g., one of the peaks is not observable. This situation would lead to incorrect attribution of the same splitting to both enantiomers and to encumbering of the alignment tensor calculation by a gross error.

Although in our experience, the numerical analysis of the RDC splitting in racemic mixtures may sometimes be speculative, in the text below, we prove unambiguously that the splitting of the signals is the result of the enantiodiscrimination of both racemates **1** and **2**.

### Alignment of Racemates **1** and **2** with the Racemate of PBLG/PBDG

To unambiguously confirm that the observed two sets of signals are signals of enantiomers and not conformers, we performed an experiment with a 1:1 mixture of PBLG and PBDG. If the polymers have sufficiently similar molecular weight distributions, polydispersities, etc., and the components are carefully weighed, a polymer mixture can be prepared that behaves like a racemate. The stereochemical relations explaining our proof are outlined in Figure 2; the racemate **E1** + **E2** with PBLG (or with PBDG) undergoes diastereomorphous interactions, and as a result, **E1** and **E2** are distinguishable in NMR spectra. However, the mixture of the racemate **E1** + **E2** with the racemate of PBLG/PBDG affords enantiomorphous interactions. Therefore, the enantiomers **E1** and **E2** are indistinguishable in NMR spectra.

**Figure 6:**
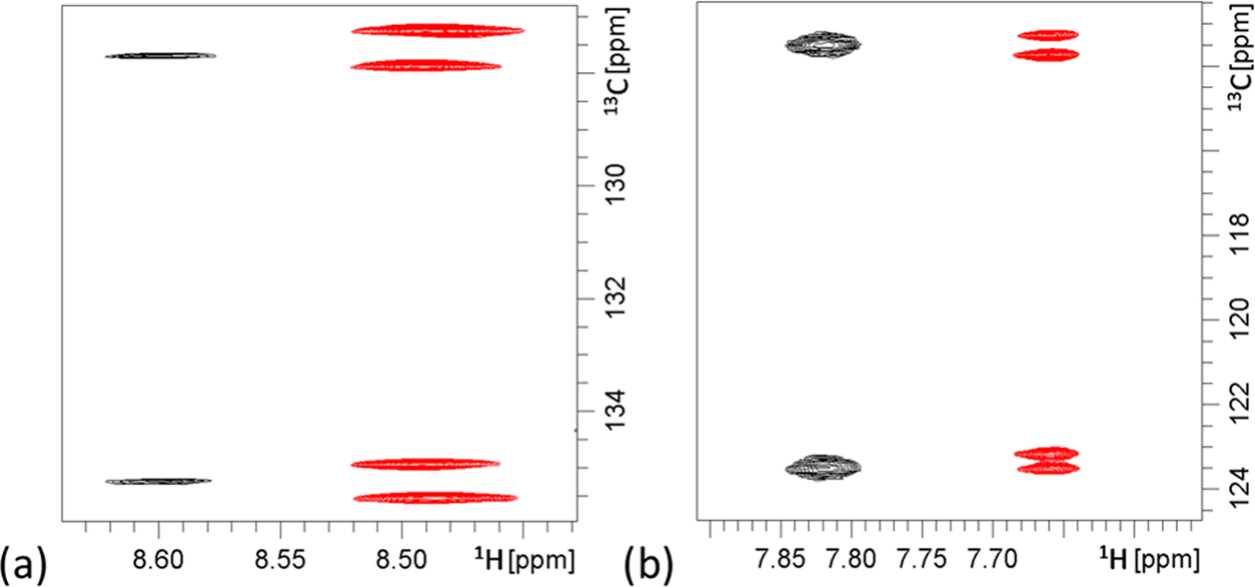
Zoomed in F1-coupled HSQC (full spectra in the Supporting Information, Figures S10–S13) of the anisotropic samples **1** (a) and **2** (b) (showing the cross-peak of B5). Red cross-peaks result from the diastereomorphous interactions measured with pure LLC-phase PBLG and THF-*d*_8_, while the black cross-peaks emerged when measured with the mixed LLC phase of PBLG and PBDG in THF-*d*_8_ (1:1).

Indeed, we observed the merging of the two sets of signals into one with an average chemical shift for both racemates **1** and **2** (see the Supporting Information, Figures S10–S13, and spectra of **1** and **2** (in THF-*d*_8_) in Figure 6), which is only explained if the respective racemate exhibits enantiomorphous interactions with the racemic polymer mixture. This unequivocally confirms that the two sets of signals are the result of enantiodiscrimination.

### Alignment of the Separated Enantiomers **1-E1**, **1-E2** and **2-E1**, **2-E2** with PBLG/THF-*d*_8_

As already mentioned, we achieved a physical separation of racemates **1** and **2** on a chiral column. This enabled us to resolve all ambiguities, investigate the alignment tensors, and, especially, evaluate differential alignment between the enantiomers. With these analyses in hand, we can also critically assess the prospects and pitfalls of the enantiodiscrimination study by means of RDC measurements of the racemic mixtures.

Due to the limited amount of physically separated enantiomers **E1** and **E2**, the alignment measurements were performed with the system of PBLG/THF-*d*_8_ only. This combination of the alignment medium and solvent was chosen due to the excellent reported data^43^ and on the basis of our results performed with racemates **1** and **2** under these conditions (see Table 1). The two samples of **1-E1** and **1-E2** (**2-E1** and **2-E2**, respectively) were prepared separately, and maximum effort was applied so that the compositions of the mixtures **E1** and **E2** and other conditions were identical (see the Supporting Information, Tables S3 and S4).

For **1**, we obtained eight or nine ^1^*D*_CH_ values for **E1** and **E2**, respectively (see Table S20), and two values corresponding to the rotating *t*-butyl groups were converted to one-bond C–C ^1^*D*_CC_. The RDC@hotFCHT^50^ fitting procedure undoubtedly identified *PaCoD* as the best-fitting structure for both enantiomers (Table 2).

**Table 2:** Weighted Quality Factors *q* of the Chromatographically Separated Enantiomers (**E1** and **E2**) of Compound **1** in PBLG and THF-*d*_8_

	*1,2-alt_AB*	*cone*	*PaCoC*	*PaCoD*	XRD (*PaCoD*)
**1-E1**	0.0436	0.0582	0.0864	0.0174	0.0209
**1-E2**	0.4434	0.5605	0.6847	0.0740	0.0683

Excellent results were obtained (Table 2, last column) also by the fitting on the crystal structure (*PaCoD*).^21^

Having calculated the alignment tensors, we could investigate the level of differences between the two enantiomers. A generalized dot product of two matrices (generalized angle β)^53^ evaluated in our case to a moderate value of 22.6° (the bigger, the more different alignment tensors). The magnitudes of the largest tensor components were very close for both enantiomers (−0.0026 and 0.0029). However, the components with the second largest magnitude deviated more strongly (0.0020 vs. −0.0028). The rhombicity of the alignment tensor of **E2** was close to the maximum value of 2/3, while for **E1**, it showed a much smaller value (0.39). This change in rhombicity, accompanied by the change in the orientation of the principle axes of the tensors, was responsible for the difference in the observed alignment of the two enantiomers. The rhombicity of E2, being very close to the maximum value, might explain the observed differences in the sign of these eigenvalues; given the E2 dataset, the numerical fitting routine found a best-fit value, which upon Eigen-decomposition resulted in a different ordering of these components (conventionally ordered by their magnitude |*zz*| > |*yy*| > |*xx*|). The corresponding Euler angles, describing the orientation of the tensor’s principle axes, were also affected by this reordering, making a simple comparison difficult (see Tables S21–S24 for an overview of the alignment parameters). We were also concerned about why we did not observe the cross-peak for all aromatic C–H couplings in the case of **E1**. The reason was that the interaction with the LLC medium led to a sizable line broadening. In the case of **E2**, all of the splittings remained observable. This is just another manifestation of the different interaction of the two enantiomers with the same LLC medium.

For **2**, we obtained seven ^1^*D*_CH_ values and one ^1^*D*_CC_ value for **E1** and **E2**, respectively (see Table S25). The RDC@hotFCHT fitting procedure on the calculated structures gave ambiguous results (Table 3). We achieved a significant improvement in the fit when the crystal structure (*1,3-alt*) was plugged into the calculation. A close inspection of the structures revealed the reason—a significantly different tilt of the D-ring between the theoretical and crystal *1,3-alt*structures. The different orientations of the aromatic rings of individual subunits suggest a higher flexibility of compound **2** compared to compound **1**, where the phenoxathiin ring reduces the degrees of freedom and no such large differences were observed in the DFT-optimized conformer compared to the XRD structure. This flexibility may also result in the ensemble-averaged geometry in the solution no longer being represented correctly by any individual DFT-optimized conformer or the XRD structure. While there are approaches to treat conformational flexibility in the employed best-fit analysis,^31,54^ the number of experimental RDCs available is too limited to simultaneously fit the alignment parameters and conformer populations associated with the ensemble averaging. We, therefore, limit the following discussion to reporting the alignment data and single-conformer analysis of the enantiodiscrimination of compound **2** for the best-fitting XRD structure without attempting an in-depth conformational analysis.

**Table 3:** Weighted Quality Factors *q* of the Chromatographically Separated Enantiomers (**E1** and **E2**) of Compound **2** in PBLG and THF-*d*_8_

	*1,2-alt_AB*	*1,2-alt_AD*	*1,3-alt*	*cone*	*PaCoA*	*PaCoB*	*PaCoC*	*PaCoD*	XRD (*1,3-alt*)
**2-E1**	0.4217	0.6589	0.8483	0.3768	0.2818	0.1290	0.6045	0.1857	0.0874
**2-E2**	0.1379	0.5951	0.6590	0.2969	0.2667	0.3192	0.4811	0.2596	0.0606

A generalized dot product of the two matrices was evaluated to a high value of 73.7°. The limited precision of the RDC fit for compound **2** explained above may change this value upon further study, but even as a first estimate based on the XRD structure, the value is significantly higher than the enantiodiscrimination observed for compound **1**. We do not have an explanation for this difference. A more detailed investigation of the underlying intermolecular interactions affecting enantiodiscrimination,^47,55^ which could help in clarifying the reasons for the difference in enantiodiscrimination, is outside of this work’s scope. The alignment tensor’s principle components for *zz* and *yy* were fairly similar for both enantiomers (*zz*: 0.0006, 0.0006; *yy*: −0.0005, −0.0004). However, the components for *xx* differed: −0.00004 and −0.0002 for **2-E1** and **2-E2**, respectively. The rhombicity of **E1** approached the maximum (0.58), while the rhombicity for **E2** reached approximately half the value (0.28). Thus, we could conclude similarly to compound **1** that the rhombicity and the change in principle axes were responsible for the different alignments of the two respective enantiomers (see Tables S26–S29 for an overview of the alignment parameters).

## Conclusions

The enantiodifferentiating ability of the LLC-based alignment media PBLG and PBPMLG in two different solvents (creating four different liquid crystal systems) have been tested to discriminate enantiomer mixtures of new types of inherently chiral phenoxathiin-based thiacalix[4]arene derivatives **1** and **2**. Enantiodiscrimination was unambiguously observed in all studied systems. However, in some cases, there were complications in the quantitative analysis of the RDC splitting in the racemic mixtures. Therefore, maximum attention was paid to (a) proving that the observed two sets of signals are the result of the occurrence of different enantiomers, not, e.g., conformers; (b) identification of the correct pairs of coupled signals; and (c) preparation of the anisotropic sample with the appropriate degree of alignment, as an unnecessarily high degree of alignment causes broadening of the signals.

To confirm the observed two sets of signals are doubtlessly signals of enantiomers and not conformers, the experiment with a racemic mixture of poly-γ-benzyl-glutamate (1:1 PBLG and PBDG) was performed. Under such conditions, we observed a merging of two sets of signals into one with an average shift. Other unequivocal proof of enantiodiscrimination was aligning of physically separated enantiomers **1-E1** and **1-E2** as well as **2-E1** and **2**-E2**** with PBLG in THF-*d*_8_. In these cases, the fitting procedures undoubtedly identified *PaCoD* as the best-fitting structure for both enantiomers **1-E1** and **1-E2** and the *1,3-alt* geometry for enantiomers **2-E2** and **2-E2**.

We also succeeded in quantifying the orientation properties of all four different liquid crystal systems of **1** (PBLG or PBPMLG in THF-*d*_8_ or CDCl_3_) and three liquid crystal systems of **2** using the generalized angle β. The LLC phases with THF-*d*_8_ exhibited a much larger degree of alignment than those with CDCl_3_, while the change of the alignment medium did not have a significant effect. To the best of our knowledge, we are the first to successfully quantitatively analyze the enantiodiscrimination using RDCs on inherently chiral systems.

## Experimental Section

### General Information

All chemicals were purchased from commercial sources and used without further purification. THF and CH_3_CN were dried using a column solvent purification system PureSolv MD7 (Inert). All samples were dried in the desiccator over P_2_O_5_ under vacuum (1 Torr) for at least 8 h. Melting points were measured on a Heiztisch Mikroskop Polytherm A (Wagner & Munz), and they were not corrected. ^1^H, ^13^C, COSY, HMQC, and HMBC spectra were measured on a Bruker Avance III 600 MHz operating at 600.13 MHz for ^1^H and 150.92 MHz for ^13^C.

Chemical shifts are given in δ-units (ppm) and are referenced to TMS or to the (residual) solvent signal. IR spectra were measured on an FTIR spectrometer Nicolet 6700 (Thermo-Nicolet) connected with a diamond ATR attachment GladiATR (PIKE) and DTGS detector. The measurement parameters were: spectral range 4000–400 cm^–1^, resolution 4 cm^–1^, 64 spectral accumulations, and Happ–Genzel apodization. ESI HRMS spectra were measured on a Q-TOF (Micromass) spectrometer. Substance purities and the reaction progress were monitored by thin layer chromatography (TLC) using silica gel 60 F_254_ on aluminum-backed sheets (Merck) and analyzed at 254 and 365 nm. Radial chromatography was carried out on a Chromatotron (Harrison Research) connected with a lab pump RHSY2 (Fluid Metering). Self-prepared glass disks were covered by silica gel 60 PF_254_ containing CaSO_4_ (Merck). Self-prepared glass plates for preparative TLC (20 × 20 cm^2^) were covered by silica gel 60 PF_254_ containing CaSO_4_ (Merck). Structural assignments were made with additional information from gCOSY, gHMQC, and gHMBC experiments.

The starting compounds **I**, **II**, **1**, and **III** were prepared according to the published procedures.^13−15^

#### Synthesis of Compound **2**

Macrocycle **II** (200 mg, 0.21 mmol) was dissolved in dry THF (20 mL), sodium methoxide (70 mg, 1.29 mmol) was added, and the solution was stirred and heated to reflux (67 °C) in an oil bath. After the entire starting compound disappeared (monitored by TLC, 3.5 h), the solvent was removed from the reaction mixture under reduced pressure. Subsequently, 1 M HCl (30 mL) was added to the residue, and the mixture was extracted with CH_2_Cl_2_ (3 × 20 mL). The combined organic layers were dried over MgSO_4_. The solvent was removed under reduced pressure to yield a crude mixture. Compound **2** was isolated using radial chromatography on silica gel (eluent CH_2_Cl_2_/isopropanol 300:1 v/v) which provided 170 mg of a white solid (82% yield). MP: 270–273 °C (CHCl_3_/MeOH).

^1^H NMR (600 MHz, CDCl_3_): δ 8.47 (d, *J* = 2.6 Hz, 1H, C-5), 8.46–8.44 (m, 1H, C-3), 8.46–8.44 (m, 1H, D-3), 8.46–8.44 (m, 1H, D-5), 8.41 (d, *J* = 2.5 Hz, 1H, B-5), 8.37 (d, *J* = 2.6 Hz, 1H, B-3), 7.80 (s,1H, A-3), 7.25 (brs, 1H, A-OH), 4.55–4.48 (m, 1H, OCH_2_-D), 4.38–4.30 (m, 1H, OCH_2_-D), 3.94 (s, 3H, OCH_3_), 4.86–3.79 (m, 1H, OCH_2_-A), 3.69–3.61 (m, 1H, OCH_2_-A), 3.56–3.47 (m, 1H, OCH_2_-C), 3.42–3.34 (m, 1H, OCH_2_-C), 1.55 (s, 9H, *t*Bu-A), 1.44 (s, 9H, *t*Bu-C), 1.43 (s, 9H, *t*Bu-D), 1.42 (s, 9H, *t*Bu-B), 1.11 (t, *J* = 6.9 Hz, 3H, CH_3_-D), 0.95 (t, *J* = 7.1 Hz, 3H, CH_3_-A), 0.57 (t, *J* = 7.0 Hz, 3H, CH_3_-C).

^1^H NMR (600 MHz, THF-*d*_8_): δ 8.63 (brs, 1H, A-OH), 8.58 (d, *J* = 2.6 Hz, 1H, C-3), 8.52 (d, *J* = 2.6 Hz, 1H, C-5), 8.49 (d, *J* = 2.6 Hz, 1H, D-3), 8.48 (d, *J* = 2.5 Hz, 1H, B-3), 8.48 (d, *J* = 2.6 Hz, 1H, D-5), 8.40 (d, *J* = 2.5 Hz, 1H, B-5), 7.84 (s,1H, A-3), 4.56-4.48 (m, 1H, OCH_2_-D), 4.35-4.28 (m, 1H, OCH_2_-D), 3.86 (s, 3H, OCH_3_), 4.78–4.71 (m, 1H, OCH_2_-C), 3.62–3.54 (m, 1H, OCH_2_-C), 3.44–3.36 (m, 1H, OCH_2_-A), 1.61 (s, 9H, *t*Bu-A), 1.49 (s, 9H, *t*Bu-C), 1.47 (s, 9H, *t*Bu-D), 1.44 (s, 9H, *t*Bu-B), 1.10 (t, *J* = 6.9 Hz, 3H, CH_3_-D), 0.93 (t, *J* = 7.0 Hz, 3H, CH_3_-A), 0.66 (t, *J* = 6.8 Hz, 3H, CH_3_-C).

^13^C NMR{^1^H} (151 MHz, CDCl_3_): δ 153.4 (quart. C, B1), 153.2 (quart. C, C1), 152.8 (quart. C, D1), 149.4 (quart. C, A1), 147.9, 146.9, 146.8 and 146.6 (quart. C, 4), 143.6 (quart. C, A6), 137.7, 137.3, 136.9, 136.7, 136.4, 135.9, 135.6 (quart. C, 2,6), 134.7 (CH, B-3), 134.3 (CH, C-3), 134.0 (CH, D-5), 133.2 (quart. C, A), 132.3 (CH, C-5), 131.5 (CH, D-3), 131.0 (CH, B-5), 120.3 (CH, A-3), 74.3 (OCH_2_-D), 72.7 (OCH_2_-C), 70.6 (OCH_2_-A), 66.0 (OCH_3_-B), 38.1 (quart. *C*–C(CH_3_)_3_-A), 35.4, 35.3 and 35.2 (quart. *C*–C(CH_3_)_3_-B, C, D), 32.5 (quart. C–*C*(CH_3_)_3_-A), 31.1, 31.1 and 31.0 (quart. C–*C*(CH_3_)_3_-B, C, D), 15.2 and 15.2 (CH_3_-A and D), 14.8 (CH_3_-C).

^13^C NMR{^1^H} (151 MHz, THF-*d*_8_): δ 153.3 (quart. C, C1), 153.2 (quart. C, B1), 152.7 (quart. C, D1), 149.6 (quart. C, A1), 147.5, 146.5, 145.9 and 145.7 (quart. C, 4), 145.6, 138.1, 137.7, 137.5, 137.34, 137.29, 137.2, 136.8 (quart. C), 135.8 (CH, B-3), 135.7 (quart. C), 134.4 (CH, C-3), 133.5 (CH, D-5), 131.8 (CH, C-5), 130.9 (CH, D-3), 129.9 (CH, B-5), 120.3 (CH, A-3), 73.5 (OCH_2_-D), 72.6 (OCH_2_-C), 69.5 (OCH_2_-A), 65.1 (OCH_3_-B), 37.6 (quart. *C*–C(CH_3_)_3_-A), 35.0, 34.9 and 34.8 (quart. *C*–C(CH_3_)_3_-B, C, D), 31.9 (quart. *C*–C(CH_3_)_3_-A), 30.4, 30.3 and 30.2 (quart. C–*C*(CH_3_)_3_-B, C, D), 14.9 (CH_3_-D), 14.7 (CH_3_-A), 14.5 (CH_3_-C).

HRMS (ESI^+^) *m/z*: calcd for (C_47_H_62_O_13_S_4_): 985.2966 [M + Na]^+^, 1001.2705 [M + K]^+^; found *m*/*z*: 985.2955 [M + Na]^+^, 1001.2699 [M + K]^+^.

### RDC Measurements and Calculations

For chemicals used for sample preparation, NMR measurement conditions, and the preparation and composition of anisotropic samples, see the Supporting Information.

The measurements of one-bond ^13^C–^1^H RDCs were carried out using the *J*-scaled F1-coupled HSQC pulse sequence providing doublets in the F1 domain due to the dipolar coupling with the components’ separation being 8-fold enhanced with respect to ^13^C chemical shift evolution. The RDCs (^1^*D*_*CH*_) were extracted using the scalar couplings, ^1^*J*_CH_, and the total couplings, ^1^*T*_CH_ (measured in aligned samples), according to the equation ^1^*T*_CH_ = 2^1^*D*_CH_ + ^1^*J*_CH_. ^1^*J*_CH_ was determined separately from an isotropic solution measurement. The experimental RDCs were evaluated using RDC@hotFCHT^50^ to calculate the alignment tensor and the corresponding back-calculated RDC values for a given structural model. The program returns several quantities to evaluate the goodness of fit. We chose the *q* factor based on the experimental-error-weighted root-mean-squared deviations as the most decisive one.

### Theoretical Calculations

Gaussian 03^56^ was used for DFT optimization of compound **1** (B3LYP^57^/6-31G*^58^) and Orca^59^ for optimizations of compound **2** (B3LYP,^57^ def2-SVP^60^ def2/J basis set, RIJCOSX^59^ approximation, D3BJ^61^ dispersion correction). All calculations were performed in vacuo.

Small imaginary frequencies (always max. −20 cm^–1^) were found in the case of **2** (*1,2-alternateAB* with inverted rings A and B, *1,3-alternate*, *cone*, *partial cone* with inverted ring A, and *partial cone* with inverted ring D). These imaginary modes were found for the *tert-*butyl groups, sometimes with the ethoxy/methoxy groups present at the lower rim. The exact position of the rotation does not concern the stability of the optimized structure nor the calculation of residual dipolar couplings, and, according to the literature, these could be neglected if the frequencies are less than tens of wavenumbers.^59,62^

## Data Availability

The data underlying this study are available in the published article and its online supplementary material.

## Supporting Information Available

Copies of ^1^H, ^13^C NMR spectra and HRMS of compound **2**, X-ray crystallographic data for compound **2**, quantum chemical calculations, and complete RDC analysis together with the corresponding results (PDF)
