# Supplementary material for: Enantiodiscrimination of Inherently Chiral Thiacalixarenes by Residual Dipolar Couplings
Source: J Org Chem. 2023 Jan 19;89(14):9711–20. doi: 10.1021/acs.joc.2c02594 (PMC11267606; doi:10.1021/acs.joc.2c02594)
Supplement: Supplementary file 1 — jo2c02594_si_001.pdf [file jo2c02594_si_001.pdf]

Supporting Information

Enantiodiscrimination of Inherently Chiral Thiacalixarenes by Residual Dipolar Couplings

Markéta Tichotová,[a], [b], [c] Tomáš Landovský,[d] Jan Lang,[g] Sharon Jeziorowski,[e] Volker Schmidts,[e] Michal Kohout,[d] Martin Babor,[f] Pavel Lhoták,*, [d] Christina M. Thiele*,[e] and Hana Dvořáková*, [a]

[a] Laboratory of NMR spectroscopy, University of Chemistry and Technology Prague, Technická 5, 166 28, Prague 6 (Czech Republic), E-mail: dvorakoh@vscht.cz

[b] Department of Physical and Macromolecular Chemistry, Faculty of Science, Charles University, 116 28 Prague, Czech Republic, E-mail: tichotom@natur.cuni.cz

[c] Institute of Organic Chemistry and Biochemistry, Czech Academy of Sciences, Flemingovo náměstí 542, 160 00 Prague, Czech Republic

[d] Department of Organic Chemistry, University of Chemistry and Technology Prague, Technická 5, 166 28, Prague 6 (Czech Republic), E-mail: lhotakp@vscht.cz

[e] Department of Chemistry, Technical University of Darmstadt, Alarich-Weiss-Str. 16, 64287 Darmstadt (Germany), E-mail: cthiele@thielelab.de

[f] Department of Solid State Chemistry, University of Chemistry and Technology Prague, Technická 5, 166 28, Prague 6 (Czech Republic)

[g] Faculty of Mathematics and Physics, Charles University, Ke Karlovu 3, 121 16, Prague 2 (Czech Republic), E-mail: Jan.Lang@mff.cuni.cz

Table of Contents

1. Spectral characterization of compound **2** ... S3

2. Preparative separation of enantiomers ... S9

3. Alignment measurements ... S10

3.1. Chemicals used for NMR samples preparation ... S10

3.2. NMR measurements ... S10

3.3. Preparation and composition of anisotropic samples ... S10

3.4. Atom numbering of compounds **1** and **2** ... S12

3.5. RDC data of racemic mixtures of **1** and **2** in PBLG/PBPMLG and $CDCl_3$/THF-d_8 ... S12

3.6. RDC data of isolated enantiomers **1-E1** and **1-E2**, **2-E1** and **2-E2** in PBLG/THF-d_8 ... S17

3.7. RDC data of racemic mixtures of **1** and **2** with racemic mixture of PBLG - PBDG (1:1) in $CDCl_3$/THF-d_8 ... S21

3.8. Spectra of **1** and **2** in the racemic mixtures of PBLG-PBDG ... S24

4. X-ray measurements ... S26

5. Theoretical Calculations ... S29

5.1. XYZ coordinates of **1** ... S29

5.2. XYZ coordinates of **2** ... S34

6. References ... S46

1. Spectral characterization of compound 2

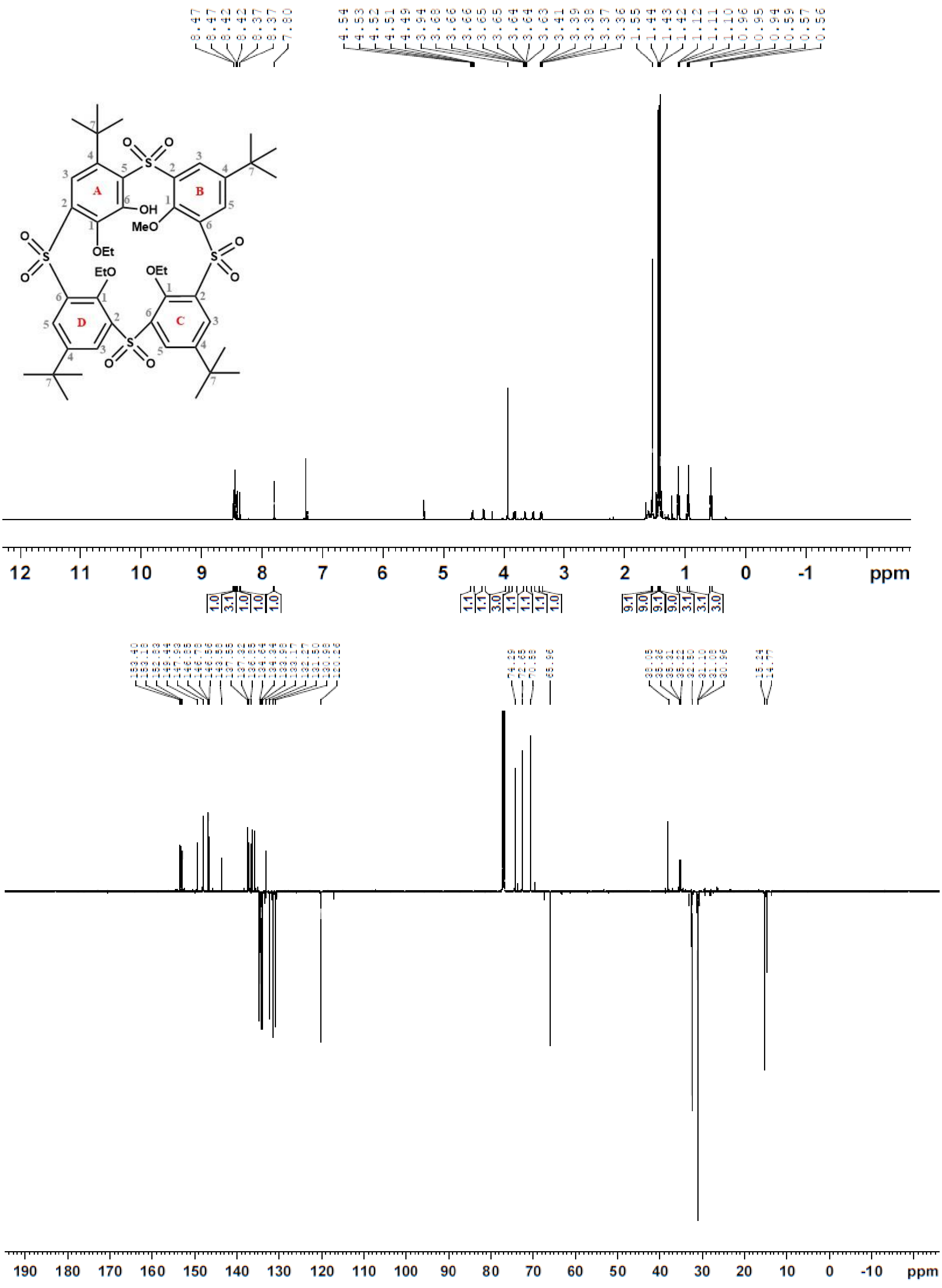

Figure S1: ^{1}H (600 MHz) (top) and $^{13}C\{^{1}H\}$ (125 MHz) (bottom) NMR spectra of **2** in $CDCl_3$ measured at room temperature.

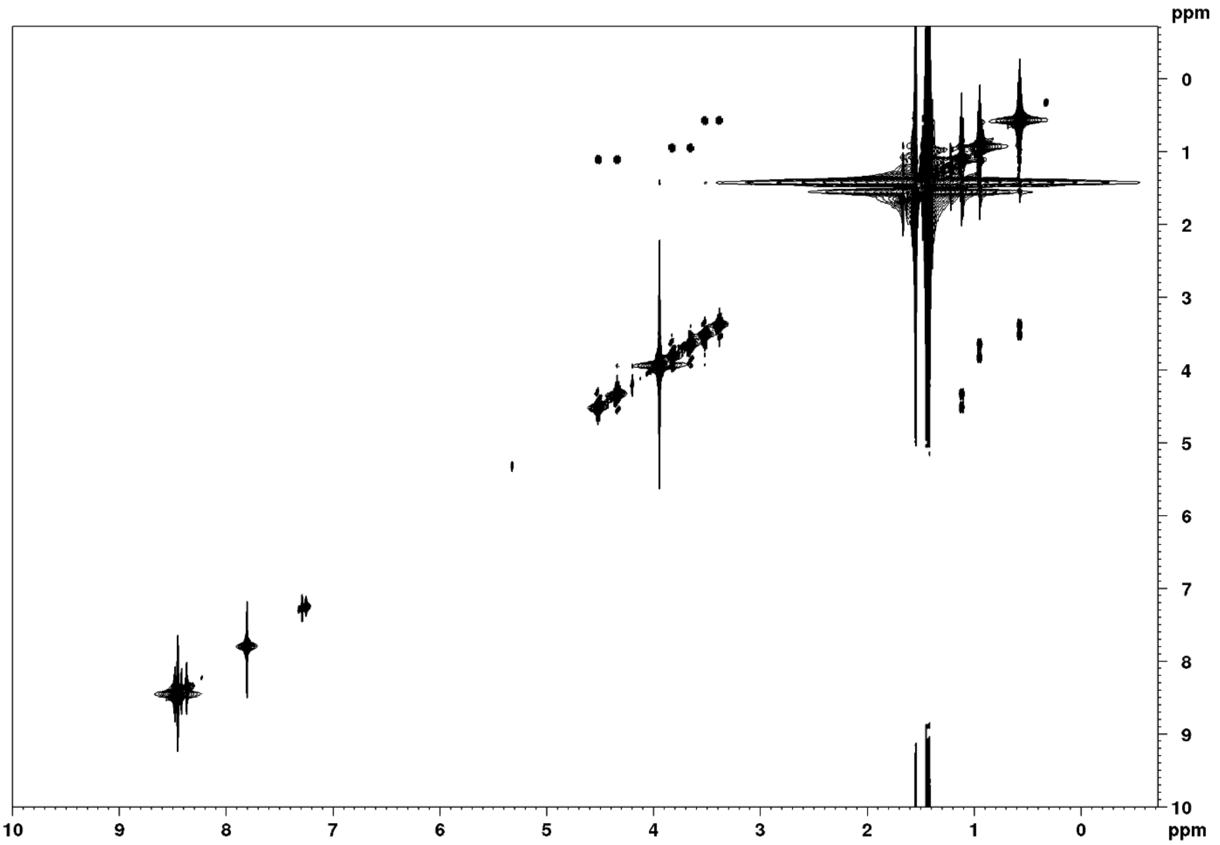

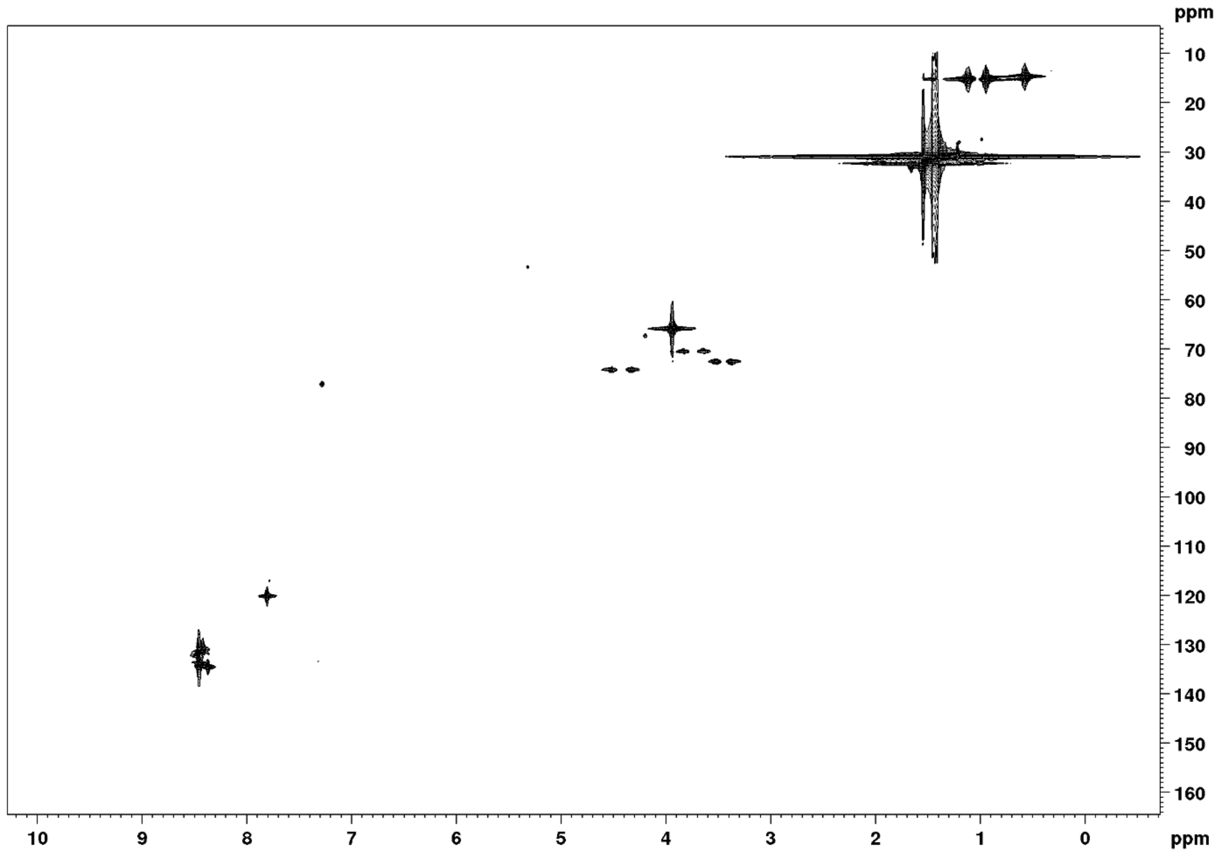

Figure S2: ^{1}H-^{1}H COSY (top) and ^{13}C-^{1}H HMQC (bottom) NMR spectra of **2** in $CDCl_3$ measured at room temperature at 600 MHz.

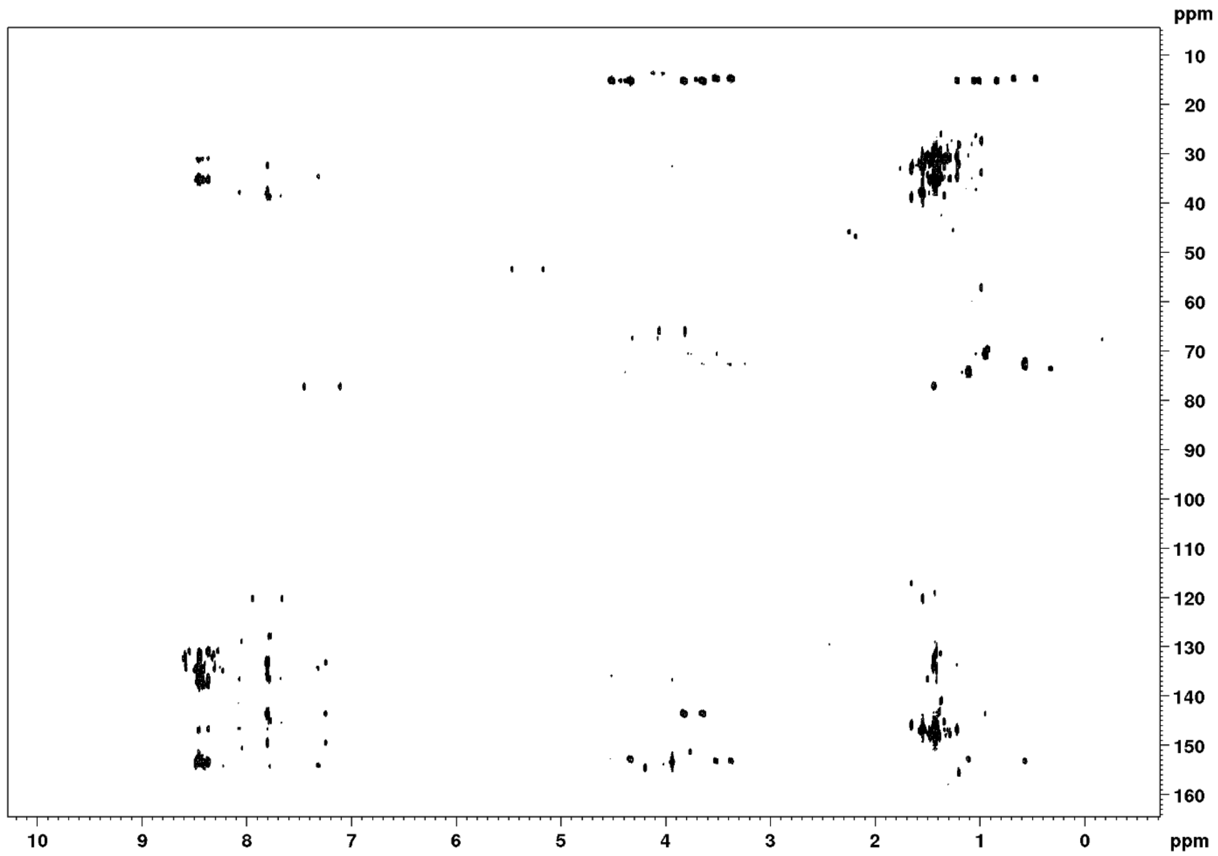

Figure S3: ^{13}C-^{1}H HMBC NMR spectra of **2** in $CDCl_3$ measured at room temperature at 600 MHz.

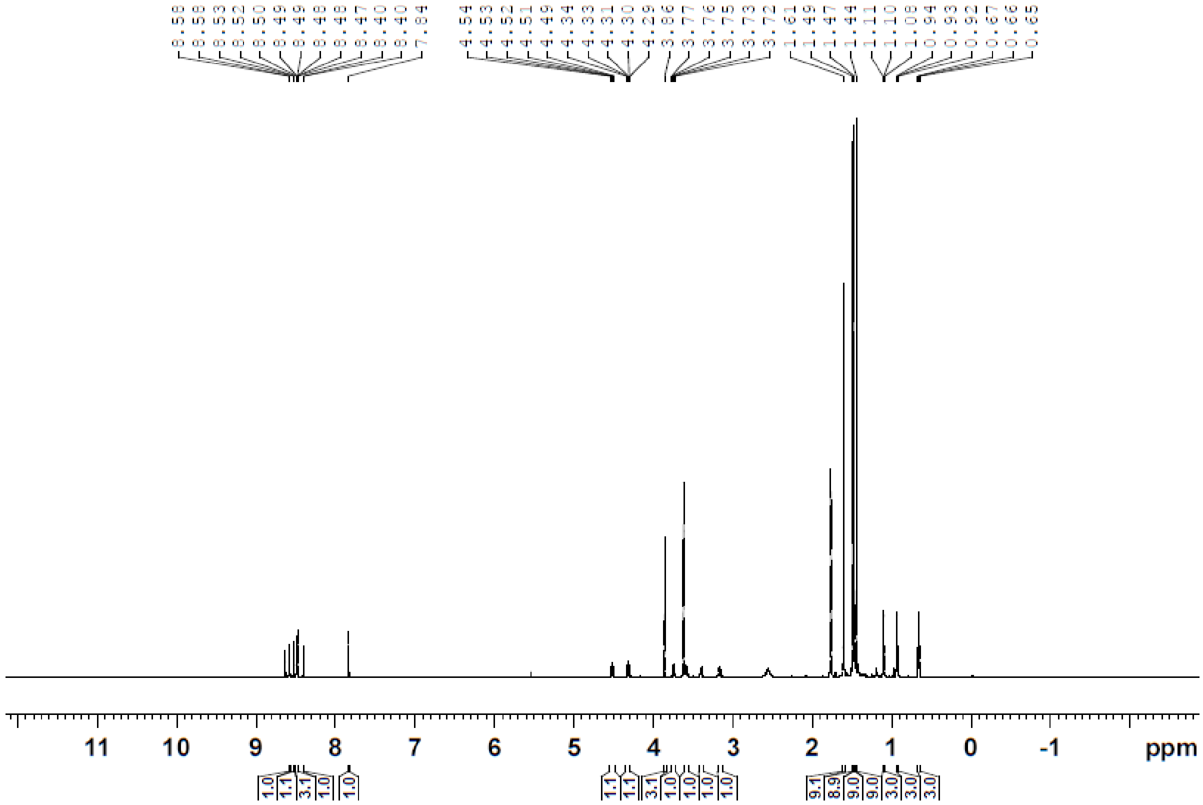

Figure S4: ^{1}H (600 MHz) NMR spectra of **2** in THF-d_8 measured at room temperature.

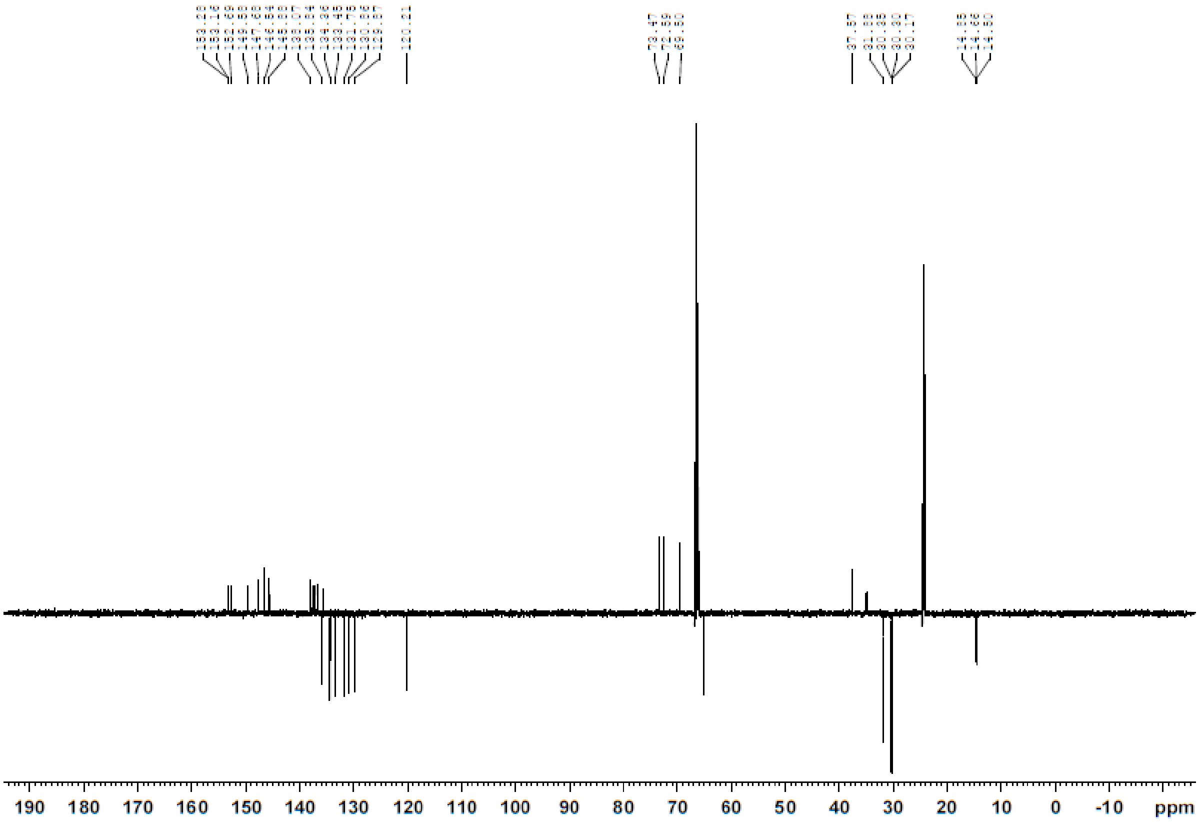

Figure S5: $^{13}C\{^{1}H\}$ (125 MHz) NMR spectra of **2** in THF-d_8 measured at room temperature.

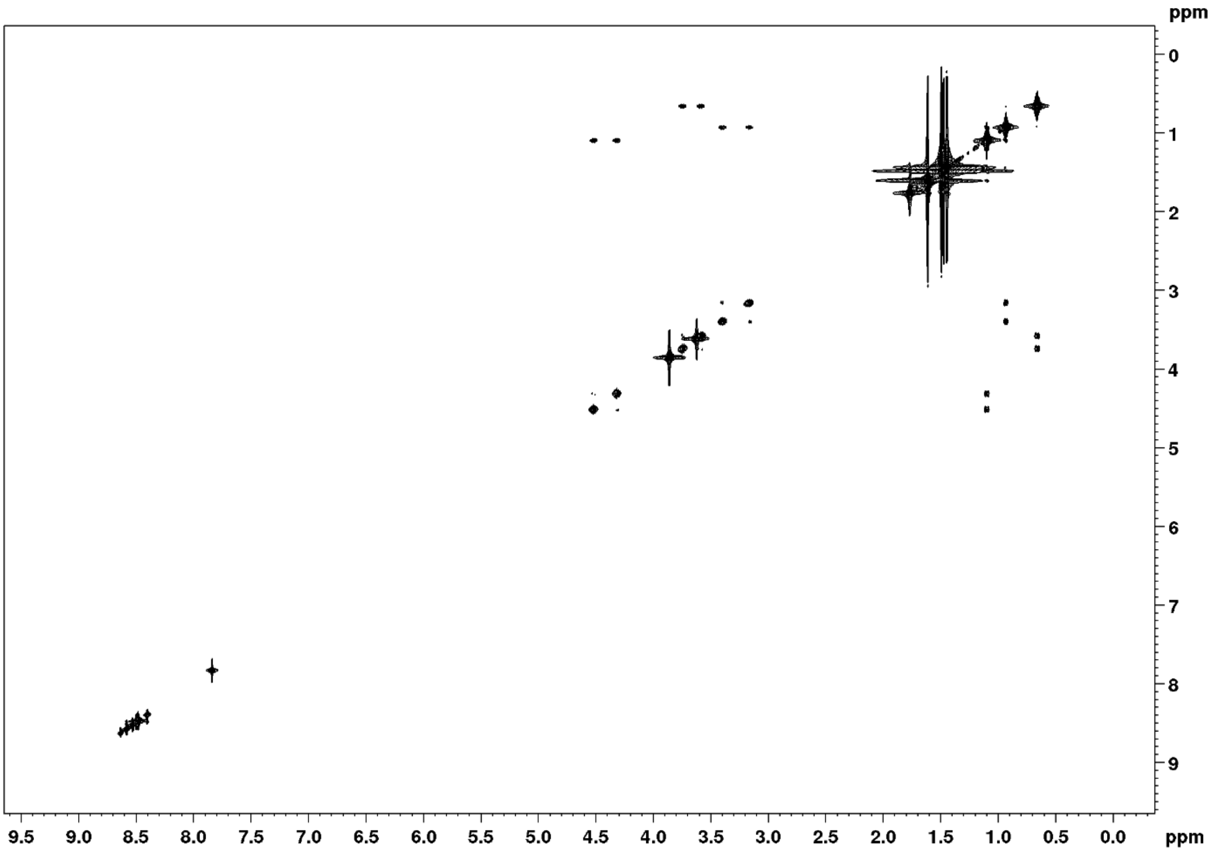

Figure S6: ^{1}H-^{1}H COSY NMR spectra of **2** in THF-d_8 measured at room temperature at 600 MHz.

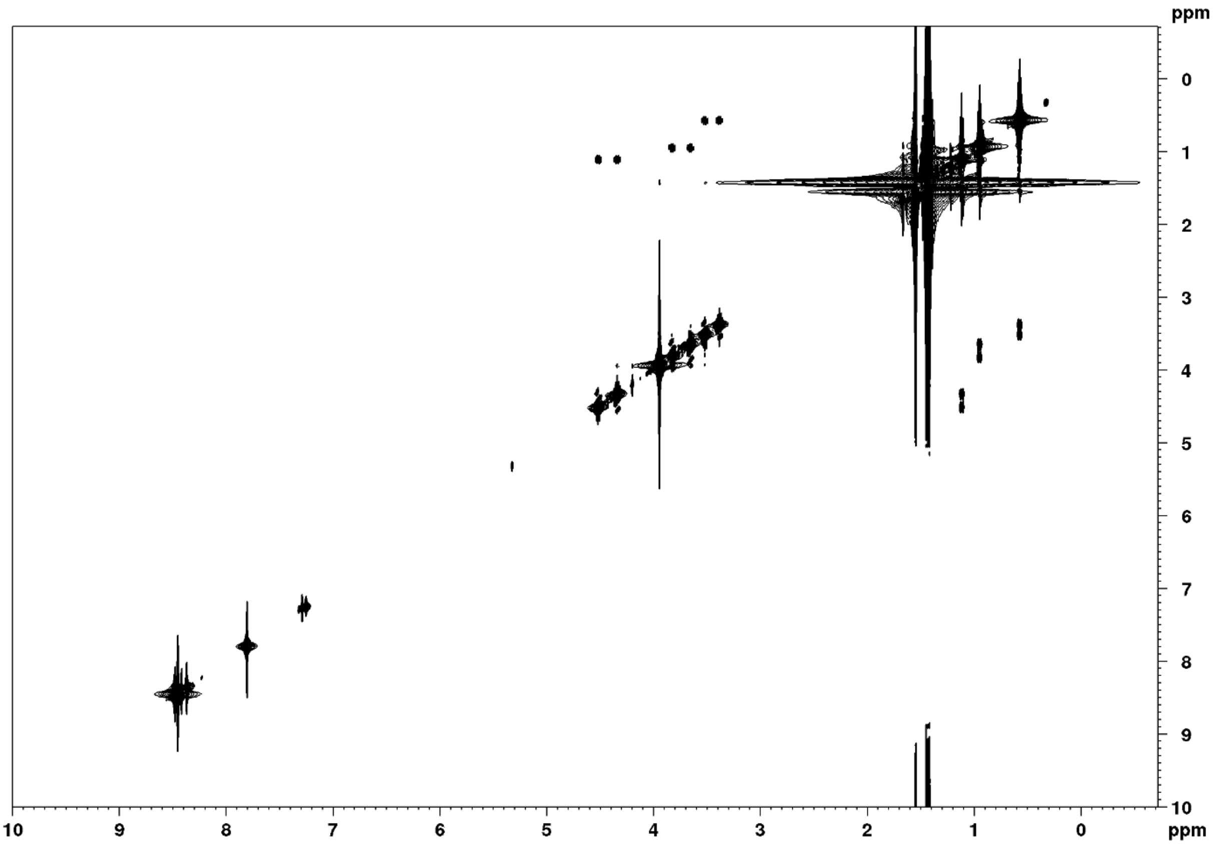

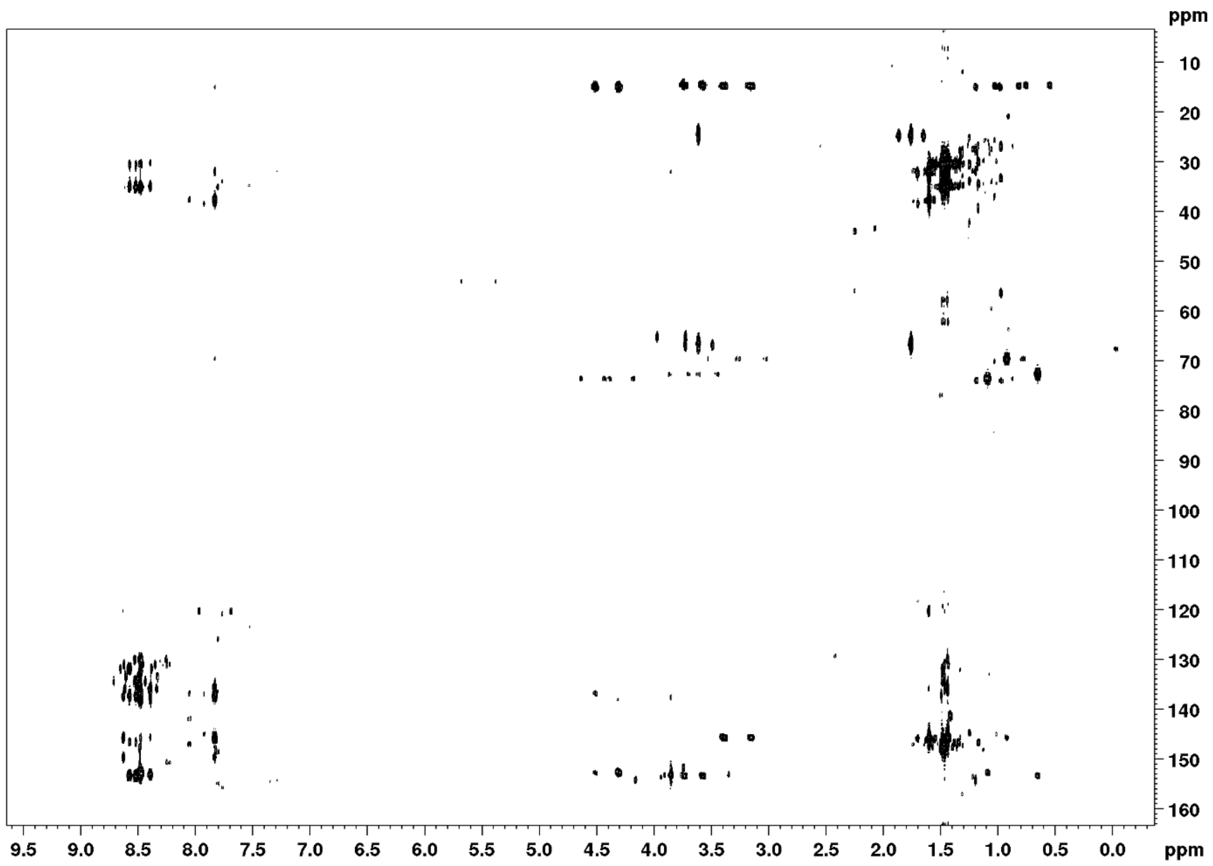

Figure S7: ^{13}C-^{1}H HMQC (top) and ^{13}C-^{1}H HMBC (bottom) NMR spectra of **2** in THF-d_8 measured at room temperature at 600 MHz.

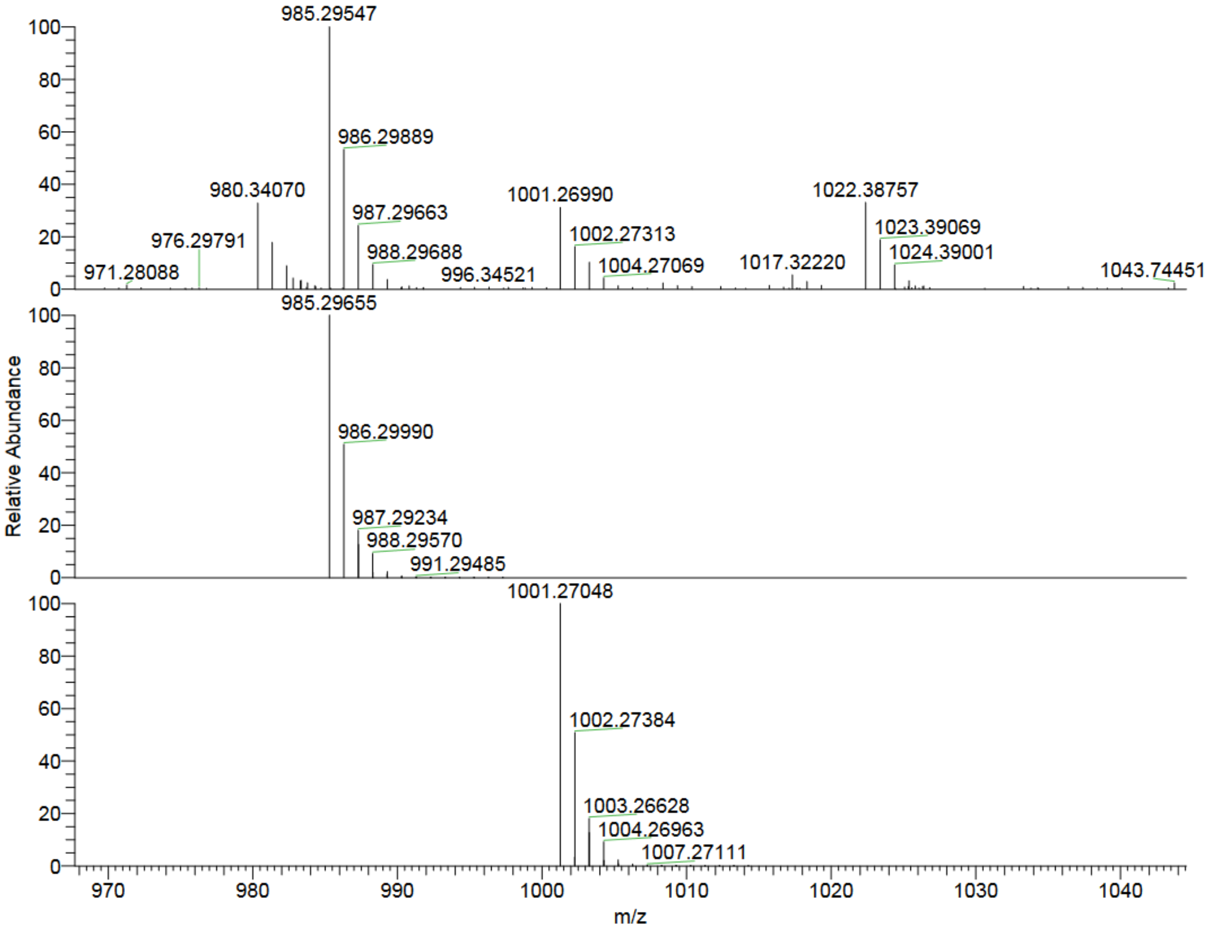

Figure S8: HR-MS spectra of **2**.

2. Preparative separation of enantiomers

Preparative chiral separation of compounds was performed using an AutoPuritication system (Waters) equipped with an automated analytical control of the collected fractions. The analytical mode was utilized to screen the chromatographic conditions for the target preparative enantioseparation using a polysaccharide-based analytical column Chiral Art Amylose-SA (250×4.6 mm i.d., 5 µm) (YMC, Germany).

After finding the best possible conditions for each substance, a chiral preparative column, Chiralpak IA (250×20 mm i.d., 5 µm) from Daicel (Japan), was employed to perform their chiral resolution on multi-milligram scale. While the separation of compounds II and III did not provide fully separated enantiomers, compounds **1** and **2** were successfully resolved.

Thus, the best conditions for chiral resolution of **2** consisted of a mobile phase comprising a heptane/propan-2-ol (96/4, v/v) mixture with the flow rate set to 15 mL•min^{-1}. The corresponding retention times were 14.98 and 22.34 min. The separation was performed at room temperature (21-23 °C, air-conditioning). Injection volume was 0.5 mL and the sample concentration was 5 mg•mL^{-1} in a heptane/propan-2-ol (1/2, v/v) mixture. The detection wavelength was 235 nm, and the automatic fraction collection was set to ensure the highest possible amount of the collected substance and the highest possible purity of the respective fractions (Figure 4, main text).

3. Alignment measurements

3.1. Chemicals used for NMR samples preparation

Chloroform-*d* ($CDCl_3$) – isotopic purity 99.8 % D (Merck, Germany); tetrahydrofuran-d_8 (THF-d_8) – isotopic purity 99.5 % D (Armar Chemicals, Germany); dimethyl sulfoxide-d_6 (DMSO) – isotopic purity 99.9 % D (Armar Chemicals, Germany); acetone-d_6 – isotopic purity 99.8 % D (Chemotrade, Germany); 1,1,2,2-tertrachloroethan-d_2 (TCE-d_2) isotopic purity 99.5 % D (Armar Chemicals, Germany); poly-*γ*-benzyl-L-glutamate (PBLG) – molecular weight 150 000 – 350 000 g/mol (Sigma-Aldrich).

Homopolypeptide-based alignment media for the measurements in the racemic mixture of poly-*γ*-benzyl-L-glutamate (PBLG[1]) and poly-*γ*-benzyl-D-glutamate (PBDG[2]) as well as poly-*γ*-*p*-biphenylmethyl-L-glutamate (PBPMLG[3]) were synthesized in the group of C. M. Thiele from Technische Universität Darmstadt (PBLG – molecular weight 554 000 g/mol, PBDG - molecular weight 111 000 g/mol, PBPMLG – molecular weight 1 150 000 g/mol).

3.2. NMR measurements

NMR spectra were recorded on spectrometer Bruker Avance III 600 MHz operating at 600.13 MHz for ^1H and 150.92 MHz for ^{13}C with 5 mm triple-resonance TCI cryoprobe. The anisotropic samples were measured using F1-coupled HSQC[4] spectra (2k data points in both the direct and the indirect dimension, NS = 8, scaling factor = 8).

3.3. Preparation and composition of anisotropic samples

The calculated amount of the alignment media was weighted directly in the NMR tube to which, subsequently, the analyte dissolved in the solvent was transferred. The amount of the alignment medium was derived from the volume of the solvent (the amount of the analyte was negligible) as well as from the molecular weight of the alignment medium itself. The weights of each part of the individual samples are in the Tables S1-S4. After mixing of all the components, the sample was left a few hours to dissolve spontaneously. Then, it was necessary to homogenize the solution due to its high viscosity. For that, manual centrifuging was used. Homogeneity of the sample was controlled by a handmade polarizer as well as by the measurement of ^2H NMR. This can reveal a possible inhomogeneity and/or residual signal of non-aligned solvent. If the latter is the case, further alignment medium needs to be added or part of the solvent evaporated.

Adequate alignment order can be also monitored by the measurement of quadrupolar splitting $\Delta\nu Q$. We took an advantage of using $CDCl_3$ and THF-d_8 as the solvents as reliable probes for examining the chiral properties of the sample. For isotropic samples, $CDCl_3$ exhibits a singlet, while in an anisotropic sample, the singlet splits into a doublet. On the other hand, THF-d_8 in ^2H NMR spectra has two doublets for the two non-equivalent methylenes in the achiral anisotropic sample, while in the chiral anisotropic sample, the signals split into four doublets.

Due to the alignment, a lock on the solvent might not be possible. Therefore, an external capillary with a different solvent is usually inserted into the sample. The choice of a suitable solvent is driven by the chemical shift of the solvent peaks in the ^{2}H experiment, as they should not interfere with the peaks of the solvent in which the alignment medium and analyte are dissolved. A DMSO-d_6 capillary was selected for the combination PBLG + $CDCl_3$ and PBLG + PBDG + $CDCl_3$, 1,1,2,2-tetrachloroethane-d_2 for PBLG + THF-d_8 as well as for PBLG + PBDG + THF-d_8 and PBPMLG + THF-d_8 and an acetone capillary for PBPMLG + $CDCl_3$.

Table S1: Quantities of individual components for all the samples involving compound **1.**

**Alignment medium**<br>**Solvent**	PBLG<br>$CDCl_3$	PBLG<br>THF-d_8	PBPMLG<br>$CDCl_3$	PBPMLG<br>THF-d_8	PBLG + PBDG<br>$CDCl_3$	PBLG + PBDG<br>THF-d_8
$m_{analyte}$ **[mg]**	12.2	12.0	14.7	14.7	13.0	12.0
$m_{solvent}$ **[mg]**	1125.0	689.5	896.4	455.9	1050.0	689.5
m_{medium}**[mg]**	93.0	101.0	79.5	95.9	L: 48.2<br>D: 48.3	L: 50.2<br>D: 50.4
w_{medium}**[%]**	7.6	12.6	8.0	16.9	8.3	12.5

Table S2: Quantities of individual components for all the samples involving compound **2.**

**Alignment medium**<br>**Solvent**	PBLG<br>$CDCl_3$	PBLG<br>THF-d_8	PBPMLG<br>$CDCl_3$	PBPMLG<br>THF-d_8	PBLG + PBDG<br>$CDCl_3$	PBLG + PBDG<br>THF-d_8
$m_{analyte}$ **[mg]**	12.1	12.6	12.0	12.5	12.1	12.6
$m_{solvent}$ **[mg]**	1050.0	613.5	814.0	453.1	1050.8	689.5
m_{medium}**[mg]**	86.6	89.7	99.3	85.3	L: 48.3<br>D: 48.3	L: 50.5<br>D: 50.5
w_{medium}**[%]**	7.5	12.5	10.7	15.5	8.3	12.6

Table S3: Quantities of individual components for samples involving isolated enantiomers **1-E1** and **1-E2.**

**Enantiomer**	**1-E1**	**1-E2**
**Alignment medium**<br>**Solvent**	PBLG<br>THF-d_8	PBLG<br>THF-d_8
$m_{analyte}$ **[mg]**	10.0	17.0
$m_{solvent}$ **[mg]**	591.0	591.0
m_{medium}**[mg]**	85.5	86.3
w_{medium}**[%]**	12.5	12.4

Table S4: Quantities of individual components for samples involving isolated enantiomers **2-E1** and **2-E2.**

**Enantiomer**	**2-E1**	**2-E2**
**Alignment medium**<br>**Solvent**	PBLG<br>THF-d_8	PBLG<br>THF-d_8
$m_{analyte}$ **[mg]**	10.8	13.3
$m_{solvent}$ **[mg]**	591.0	591.0
m_{medium}**[mg]**	86.4	86.4
w_{medium}**[%]**	12.6	12.5

3.4. Atom numbering of compounds 1 and 2

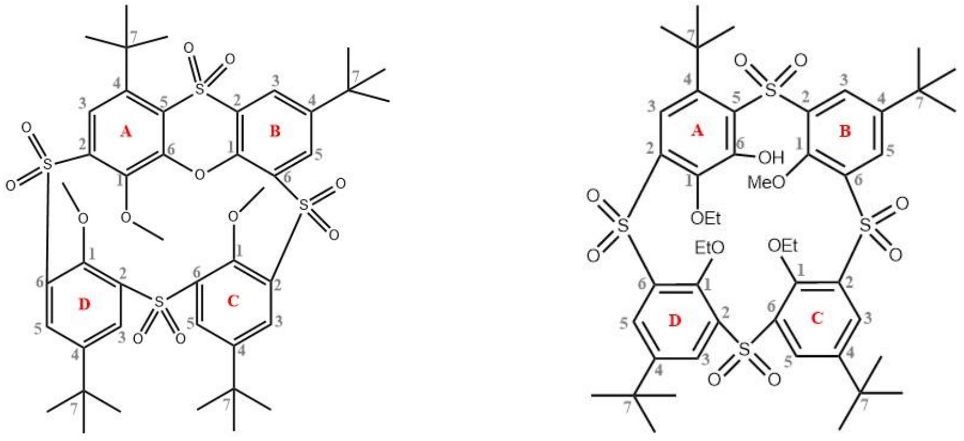

Figure S9. Numbered structures of **1** (left) and **2** (right).

3.5. RDC data of racemic mixtures of 1 and 2 in PBLG/PBPMLG and $CDCl_3$/THF-d_8

The assignment of the RDCs to **E1** or **E2** of the racemic mixtures of compounds **1** and **2** was performed by systematic variation of all possible assignments and subsequent selection of the combination with the best uncertainty-weighted quality factor *q*.[5]

Due to the uncertainties in the conformational assignment, we fit the RDC data of the racemic mixtures also to the structures obtained by X-ray diffraction.

Table S5. Weighted quality factors *q* for both enantiomers (**E1** and **E2**) of racemic mixtures **1** and **2** within PBLG and PBPMLG alignment media in $CDCl_3$ or THF-d_8.

	**PBLG/$CDCl_3$**	**PBLG /THF-d_8**	**PBPMLG/$CDCl_3$**	**PBPMLG/THF-d_8**
**1 E1** (*PaCoD*)	0.0999	0.1266	0.1120	0.0143
**1 E2** (*PaCoD*)	0.0643	0.0672	0.0491	0.0295
**2 E1** (*1,3-alt*)	n.d.*	0.4411	n.d.**	0.0300
**2 E2** (*1,3 -alt*)	n.d.*	0.2432	n.d.**	0.0511

* All *q* factors were very similar with values around 0.1, therefore, we were not able to determine the correct conformation.

** The measurements of the racemic mixture of compound **2** in the system PBPMLG/$CDCl_3$ did not provide any results.

Table S6. Weighted quality factors *q* for both enantiomers (**E1** and **E2**) of the racemic mixture of **1** in PBLG and $CDCl_3$.

	1,2-altAB	***Cone***	***PaCoC***	***PaCoD***
**E1**	0.3133	0.3392	0.4762	0.0999
**E2**	0.2071	0.3599	0.3374	0.0643

Table S7. One bond residual dipolar couplings ^1D(C-H) or ^1D(C-C) for both enantiomers (**E1** and **E2**) of the racemic mixture of **1** in PBLG/$CDCl_3$ at 300 K extracted from F1-coupled HSQC spectra.

	**E1** D_{exp} **[Hz]**	**E1** **Error [Hz]**	**E2** D_{exp} **[Hz]**	**E2** **Error [Hz]**
**A3**	11.27	0.35	13.60	0.22
**B3**	−35.00	0.63	−31.18	0.65
**B5**	−6.58	0.10	−4.34	0.46
**C3**	–	–	–	–
**C5**	–	–	–	–
**D3**	−13.18	0.17	−13.18	0.17
**D5**	14.19	0.70	11.86	0.09
***t*Bu A**	−0.38	0.03	−0.38	0.03
***t*Bu C**	1.31	0.09	1.31	0.09

Table S8. Weighted quality factors *q* for both enantiomers (**E1** and **E2**) of the racemic mixture of **1** in PBLG and THF-d_8.

	1,2-altAB	***Cone***	***PaCoC***	***PaCoD***	**XRD (*PaCoD*)**
**E1**	0.2762	0.3285	0.4916	0.1266	0.1196
**E2**	0.5921	0.4806	0.6327	0.0672	0.0517

Table S9. One bond residual dipolar couplings ^1D(C-H) or ^1D(C-C) for both enantiomers (**E1** and **E2**) of the racemic mixture of **1** in PBLG/THF-d_8 at 300 K extracted from F1-coupled HSQC spectra.

	**E1** D_{exp} **[Hz]**	**E1** **Error [Hz]**	**E2** D_{exp} **[Hz]**	**E2** **Error [Hz]**
**A3**	10.01	0.62	10.01	0.62
**B3**	−22.70	0.08	−43.57	0.21
**B5**	−17.43	0.06	−5.75	0.07
**C3**	−42.28	0.31	−42.28	0.31
**C5**	34.20	0.17	34.20	0.17
**D3**	−26.99	0.24	−23.21	0.10
**D5**	2.22	0.08	23.61	0.12
***t*Bu A**	–	–	–	–
***t*Bu C**	0.26	0.03	0.26	0.03

Table S10. Weighted quality factors *q* for both enantiomers (**E1** and **E2**) of the racemic mixture of **1** in PBPMLG and $CDCl_3$.

	1,2-altAB	***Cone***	***PaCoC***	***PaCoD***
**E1**	0.4521	0.2524	0.4806	0.1120
**E2**	0.2839	0.1132	0.2516	0.0491

Table S11. One bond residual dipolar couplings ^1D(C-H) or ^1D(C-C) for both enantiomers (**E1** and **E2**) of the racemic mixture of **1** in PBPMLG/$CDCl_3$ at 300 K extracted from F1-coupled HSQC spectra.

	**E1** D_{exp} **[Hz]**	**E1** **Error [Hz]**	**E2** D_{exp} **[Hz]**	**E2** **Error [Hz]**
**A3**	5.66	0.28	11.32	0.05
**B3**	−16.65	0.46	−16.65	0.46
**B5**	−13.69	0.17	−9.65	0.19
**C3**	−27.48	0.64	−27.48	0.64
**C5**	23.58	0.41	21.17	0.44
**D3**	−19.65	0.22	−19.65	0.22
**D5**	0.43	0.20	0.43	0.20
***t*Bu A**	0.00	0.08	0.00	0.08
***t*Bu C**	–	–	–	–

Table S12. Weighted quality factors *q* for both enantiomers (**E1** and **E2**) of the racemic mixture of **1** in PBPMLG and THF-d_8.

	1,2-altAB	***Cone***	***PaCoC***	***PaCoD***
**E1**	0.0215	0.0154	0.0441	0.0143
**E2**	0.1153	0.0554	0.1021	0.0295

Table S13. One bond residual dipolar couplings ^1D(C-H) or ^1D(C-C) for both enantiomers (**E1** and **E2**) of the racemic mixture of **1** in PBPMLG/THF-d_8 at 300 K extracted from F1-coupled HSQC spectra.

	**E1** D_{exp} **[Hz]**	**E1** **Error [Hz]**	**E2** D_{exp} **[Hz]**	**E2** **Error [Hz]**
**A3**	27.73	0.02	27.73	0.02
**B3**	−20.56	0.10	−20.56	0.10
**B5**	−21.62	0.04	−10.26	0.02
**C3**	7.82	0.29	−6.04	0.09
**C5**	18.74	0.01	18.74	0.01
**D3**	−41.44	0.54	−41.44	0.54
**D5**	−20.17	0.20	5.19	0.14
***t*Bu A**	–	–	–	–
***t*Bu C**	–	–	–	–

Table S14. Weighted quality factors *q* for both enantiomers (**E1** and **E2**) of the racemic mixture of **2** in PBLG and $CDCl_3$.

	1,2-altAB	***1,2-altAD***	***1,3-alt***	***Cone***	***PaCoA***	***PaCoB***	***PaCoC***	***PaCoD***
**E1**	0.0121	0.0526	0.0171	0.1114	0.0096	0.0213	0.0294	0.0180
**E2**	0.0180	0.0488	0.0187	0.1864	0.0180	0.0243	0.0320	0.0217

Table S15. One bond residual dipolar couplings ^1D(C-H) or ^1D(C-C) for both enantiomers (**E1** and **E2**) of the racemic mixture of **2** in PBLG/$CDCl_3$ at 300 K extracted from F1-coupled HSQC spectra.

	**E1** D_{exp} **[Hz]**	**E1** **Error [Hz]**	**E2** D_{exp} **[Hz]**	**E2** **Error [Hz]**
**A3**	18.33	0.02	18.33	0.02
**B3**	−2.84	0.11	−2.84	0.11
**B5**	−1.22	0.47	1.21	0.49
**C3**	–	–	–	–
**C5**	−9.86	0.04	−9.86	0.04
**D3**	−9.34	0.08	−9.34	0.08
**D5**	−8.67	0.01	−8.67	0.01
***t*Bu A**	1.40	0.22	1.63	0.12
***t*Bu C**	–	–	–	–

Table S16. Weighted quality factors *q* for both enantiomers (**E1** and **E2**) of the racemic mixture of **2** in PBLG and THF-d_8.

	1,2-altAB	***1,2-altAD***	***1,3-alt***	***Cone***	***PaCoA***	***PaCoB***	***PaCoC***	***PaCoD***	**XRD (*1,3-alt*)**
**E1**	0.2626	0.4659	0.4411	0.3424	0.1886	0.1310	0.4718	0.0635	0.1498
**E2**	0.2106	0.5263	0.2432	0.4053	0.2355	0.1570	0.8343	0.1482	0.1658

Table S17. One bond residual dipolar couplings ^1D(C-H) or ^1D(C-C) for both enantiomers (**E1** and **E2**) of the racemic mixture of **2** in PBLG/THF-d_8 at 300 K extracted from F1-coupled HSQC spectra.

	**E1** D_{exp} **[Hz]**	**E1 Error [Hz]**	**E2** D_{exp} **[Hz]**	**E2 Error [Hz]**
**A3**	13.17	0.11	5.46	0.05
**B3**	−14.40	0.15	2.25	0.14
**B5**	−0.08	0.31	−0.80	0.31
**C3**	−12.51	0.11	−18.54	0.10
**C5**	−15.20	0.23	−15.20	0.23
**D3**	−8.44	0.46	−6.45	0.43
**D5**	5.68	0.02	10.70	0.14
***t*Bu A**	0.96	0.02	0.96	0.02
***t*Bu C**	–	–	–	–

Table S18. Weighted quality factors *q* for both enantiomers (**E1** and **E2**) of the racemic mixture of **2** in PBPMLG and THF-d_8.

	1,2-altAB	***1,2-altAD***	***1,3-alt***	***Cone***	***PaCoA***	***PaCoB***	***PaCoC***	***PaCoD***
**E1**	0.1257	0.2582	0.0300	0.1829	0.2699	0.3417	0.1853	0.3775
**E2**	0.1011	0.2557	0.0511	0.2137	0.2483	0.3916	0.2313	0.4460

Table S19. One bond residual dipolar couplings ^1D(C-H) or ^1D(C-C) for both enantiomers (**E1** and **E2**) of the racemic mixture of **2** in PBPMLG/ THF-d_8 at 300 K extracted from F1-coupled HSQC spectra.

	E1<br>D_{exp} [Hz]	E1<br>Error [Hz]	E2<br>D_{exp} [Hz]	E2<br>Error [Hz]
**A3**	0.96	0.39	4.40	0.21
**B3**	21.59	0.32	21.59	0.32
**B5**	−14.70	0.28	−14.70	0.28
**C3**	−8.38	0.03	−8.38	0.03
**C5**	15.83	0.24	3.45	0.10
**D3**	−11.84	0.68	−11.84	0.68
**D5**	24.66	0.21	15.09	0.08
***t*Bu A**	−1.37	0.24	−1.37	0.24
***t*Bu C**	–	–	–	–

3.6. RDC data of isolated enantiomers 1-E1 and 1-E2, 2-E1 and 2-E2 in PBLG/THF-d_8

Table S20. One bond residual dipolar couplings ^1D(C-H) or ^1D(C-C) of **1-E1** and **1-E2** in PBLG/THF-d_8 at 300 K extracted from F1-coupled HSQC spectra.

	E1<br>D_{exp} [Hz]	E1<br>Error [Hz]	E2<br>D_{exp} [Hz]	E2<br>Error [Hz]
**A3**	12.28	0.04	14.66	0.05
**B3**	−27.91	0.01	−51.58	0.07
**B5**	−21.41	0.06	−6.97	0.05
**C3**	–	–	−50.34	0.11
**C5**	43.03	0.17	40.75	0.09
**D3**	−33.57	0.23	−27.93	0.07
**D5**	2.76	0.07	27.99	0.03
***t*Bu A**	0.02	0.06	1.78	0.05
***t*Bu C**	2.76	0.05	0.39	0.01

Table S21. Orientational properties of *PaCoD* conformation of **1-E1** in PBLG/THF-d_8.

	**1-E1**
**Euler α**	82.40
**Euler β**	9.17
**Euler γ**	144.44
**Da [10^{-3}]**	-1.29
**Dr [10^{-4}]**	-5.00

Table S22. Tensor parameters of *PaCoD* conformation of **1-E1** in PBLG/THF-d_8.

**Saupe Vector (Losonczi Method)**					
	**zz**	**xx-yy**	**xy**	**xz**	**yz**
**Average**	$-2.46\cdot10^{-3}$	$6.35\cdot10^{-5}$	$6.93\cdot10^{-4}$	$-5.68\cdot10^{-4}$	$-4.45\cdot10^{-4}$
**Error**	$1.20\cdot10^{-5}$	$9.40\cdot10^{-6}$	$6.21\cdot10^{-6}$	$1.21\cdot10^{-5}$	$4.49\cdot10^{-6}$
**% Error**	0.49	14.81	0.90	2.13	1.01
**Saupe Order Matrix**					
	$1.26\cdot10^{-3}$	$6.93\cdot10^{-4}$	$-5.67\cdot10^{-4}$		
	$6.93\cdot10^{-4}$	$1.20\cdot10^{-3}$	$-4.45\cdot10^{-4}$		
	$-5.67\cdot10^{-4}$	$-4.45\cdot10^{-4}$	$-2.46\cdot10^{-3}$		
**Eigenvalues of Saupe Matrix**					
**xx**	$5.36\cdot10^{-4}$				
**yy**	$2.04\cdot10^{-3}$				
**zz**	$-2.57\cdot10^{-3}$				

Table S23. Orientational properties of *PaCoD* conformation of **1-E2** in PBLG/THF-d_8.

	**1-E2**
**Euler α**	96.90
**Euler β**	81.35
**Euler γ**	-24.73
**Da [10^{-3}]**	1.47
**Dr [10^{-4}]**	8.91

Table S24. Tensor parameters of *PaCoD* conformation of **1-E2** in PBLG/THF-d_8.

Saupe Vector (Losonczi Method)					
	**zz**	**xx-yy**	**xy**	**xz**	**yz**
**Average**	$-2.64\cdot10^{-3}$	$1.87\cdot10^{-3}$	$1.16\cdot10^{-3}$	$-9.03\cdot10^{-4}$	$-6.88\cdot10^{-5}$
**Error**	$4.12\cdot10^{-6}$	$4.04\cdot10^{-6}$	$3.27\cdot10^{-6}$	$3.82\cdot10^{-6}$	$1.93\cdot10^{-6}$
**% Error**	0.16	0.22	0.28	0.42	2.81
**Saupe Order Matrix**					
	$2.25\cdot10^{-3}$	$1.16\cdot10^{-3}$	$-9.03\cdot10^{-4}$		
	$1.16\cdot10^{-3}$	$3.87\cdot10^{-4}$	$-6.88\cdot10^{-5}$		
	$-9.03\cdot10^{-4}$	$-6.88\cdot10^{-5}$	$-2.64\cdot10^{-3}$		
**Eigenvalues of Saupe Matrix**					
**xx**	$-1.33\cdot10^{-4}$				
**yy**	$-2.81\cdot10^{-3}$				
**zz**	$2.94\cdot10^{-3}$				

Table S25. One bond residual dipolar couplings ^{1}D(C-H) or ^{1}D(C-C) of **2-E1** and **2-E2** in PBLG/THF-d_8 at 300 K extracted from F1-coupled HSQC spectra.

	**E1** D_{exp} **[Hz]**	**E1** **Error [Hz]**	**E2** D_{exp} **[Hz]**	**E2** **Error [Hz]**
**A3**	11.70	0.12	5.40	0.05
**B3**	−12.98	0.06	1.94	0.16
**B5**	−0.04	0.02	−1.99	0.08
**C3**	−11.30	0.09	−17.14	0.15
**C5**	−13.28	0.23	−14.24	0.38
**D3**	−8.19	0.10	−5.69	0.15
**D5**	5.63	0.07	9.36	0.04
***t*Bu A**	1.06	0.08	0.75	0.20

Table S26. Orientational properties of *1,3-alternate* conformation of **2-E1** in PBLG/THF-d_8.

	**2-E1**
**Euler α**	-53.81
**Euler β**	128.73
**Euler γ**	-76.62
**Da [10^{-4}]**	2.89
**Dr [10^{-4}]**	1.69

Table S27. Tensor parameters of *1,3-alternate* conformation of **2-E1** in PBLG/THF-d_8.

**Saupe Vector (Losonczi Method)**					
	**zz**	**xx-yy**	**xy**	**xz**	**yz**
**Average**	$3.67{\cdot}10^{-6}$	$-2.40{\cdot}10^{-4}$	$2.29{\cdot}10^{-4}$	$-7.64{\cdot}10^{-5}$	$4.91{\cdot}10^{-4}$
**Error**	$7.99{\cdot}10^{-6}$	$1.61{\cdot}10^{-5}$	$3.84{\cdot}10^{-6}$	$3.33{\cdot}10^{-6}$	$4.96{\cdot}10^{-6}$
**% Error**	217.99	6.70	1.67	4.36	1.01
**Saupe Order Matrix**					
	$-1.22{\cdot}10^{-4}$	$2.29{\cdot}10^{-4}$	$-7.66{\cdot}10^{-5}$		
	$2.29{\cdot}10^{-4}$	$1.19{\cdot}10^{-4}$	$4.91{\cdot}10^{-4}$		
	$-7.66{\cdot}10^{-5}$	$4.91{\cdot}10^{-4}$	$3.50{\cdot}10^{-6}$		
**Eigenvalues of Saupe Matrix**					
**xx**	$-3.59{\cdot}10^{-5}$				
**yy**	$-5.42{\cdot}10^{-4}$				
**zz**	$5.78{\cdot}10^{-4}$				

Table S28. Orientational properties of *1,3-alternate* conformation of **2-E2** in PBLG/THF-d_8.

	**2-E2**
**Euler α**	39.37
**Euler β**	118.67
**Euler γ**	-31.07
**Da [10^{-4}]**	2.82
**Dr [10^{-5}]**	7.97

Table S29. Tensor parameters of *1,3-alternate* conformation of **2-E2** in PBLG/THF-d_8.

Saupe Vector (Losonczi Method)					
	zz	xx-yy	xy	xz	yz
**Average**	$-6.93\cdot10^{-5}$	$2.19\cdot10^{-4}$	$3.27\cdot10^{-4}$	$3.50\cdot10^{-4}$	$9.09\cdot10^{-5}$
**Error**	$1.02\cdot10^{-5}$	$2.61\cdot10^{-5}$	$4.99\cdot10^{-6}$	$5.47\cdot10^{-6}$	$8.21\cdot10^{-6}$
**% Error**	14.69	11.92	1.53	1.56	9.03
**Saupe Order Matrix**					
	$1.45\cdot10^{-4}$	$3.27\cdot10^{-4}$	$3.50\cdot10^{-4}$		
	$3.27\cdot10^{-4}$	$-7.59\cdot10^{-5}$	$9.05\cdot10^{-5}$		
	$3.50\cdot10^{-4}$	$9.05\cdot10^{-5}$	$-6.86\cdot10^{-5}$		
**Eigenvalues of Saupe Matrix**					
**xx**	$-1.63\cdot10^{-4}$				
**yy**	$-4.02\cdot10^{-4}$				
**zz**	$5.65\cdot10^{-4}$				

3.7. RDC data of racemic mixtures of 1 and 2 with racemic mixture of PBLG - PBDG (1:1) in $CDCl_3$/THF-d_8

Table S30. Weighted quality factors *q* of the racemic mixture **1** in the racemic mixture of PBLG-PBDG and $CDCl_3$.

1,2-altAB	***Cone***	***PaCoC***	***PaCoD***	**XRD (*PaCoD*)**
0.1220	0.1757	0.1141	0.0220	0.0588

Table S31. One bond residual dipolar couplings ^{1}D(C-H) and ^{1}D(C-C) of racemic mixture **1** in the racemic mixture of PBLG-PBDG and $CDCl_3$ at 300 K extracted from F1-coupled HSQC spectra.

	D_{exp} [Hz]	Error [Hz]
**A3**	11.65	0.04
**B3**	−30.77	0.14
**B5**	−5.41	0.26
**C5**	50.17	0.22
**D3**	−12.60	0.06
**D5**	11.89	0.18
**tBu A**	−0.34	0.07
**tBu D**	1.95	0.18

Table S32. Weighted quality factors *q* of the racemic mixture **1** in the racemic mixture of PBLG-PBDG and THF-d_8.

1,2-altAB	***Cone***	***PaCoC***	***PaCoD***	**XRD (*PaCoD*)**
0.1446	0.1460	0.3781	0.0462	0.0367

Table S33. One bond residual dipolar couplings ^1D(C-H) of racemic mixture **1** in the racemic mixture of PBLG-PBDG and THF-d_8 at 300 K extracted from F1-coupled HSQC spectra.

	D_{exp} **[Hz]**	**Error [Hz]**
**A3**	12.17	0.07
**B3**	−36.27	0.06
**B5**	−12.72	0.13
**C3**	−47.46	0.48
**C5**	37.73	0.09
**D3**	−27.46	0.45
**D5**	14.30	0.01

Table S34. Weighted quality factors *q* of the racemic mixture **2** in the racemic mixture of PBLG-PBDG and $CDCl_3$.

1,2-altAB	***1,2-altAD***	***1,3-alt***	***Cone***	***PaCoA***	***PaCoB***	***PaCoC***	***PaCoD***	**XRD (*1,3-alt*)**
0.2585	0.0279	0.0383	0.3606	0.0888	0.4402	0.2348	0.3525	0.1904

Table S35. One bond residual dipolar couplings ^1D(C-H) and ^1D(C-C) of racemic mixture **2** in the racemic mixture of PBLG-PBDG and $CDCl_3$ at 300 K extracted from F1-coupled HSQC spectra.

	D_{exp} **[Hz]**	**Error [Hz]**
**A3**	23.64	0.11
**B3**	-7.14	0.08
**B5**	3.36	0.14
**C5**	−10.68	0.39
**D3**	0.81	0.56
**D5**	−13.34	0.14
**tBu A**	1.77	0.03

Table S36. Weighted quality factors *q* of the racemic mixture **2** in the racemic mixture of PBLG-PBDG and THF-d_8.

1,2-altAB	***1,2-altAD***	***1,3-alt***	***Cone***	***PaCoA***	***PaCoB***	***PaCoC***	***PaCoD***	**XRD (*1,3-alt*)**
0.2628	0.8132	0.4653	0.5469	0.3144	0.1672	0.7756	0.1220	0.2457

Table S37. One bond residual dipolar couplings ${}^{1}D$(C-H) and ${}^{1}D$(C-C) of racemic mixture **2** in the racemic mixture of PBLG-PBDG and THF-d_8 at 300 K extracted from F1-coupled HSQC spectra.

	D_{exp} **[Hz]**	**Error [Hz]**
**A3**	10.63	0.25
**B3**	−8.24	0.56
**B5**	−1.81	0.18
**C3**	−15.96	0.19
**C5**	−15.20	0.38
**D3**	−9.34	0.22
**D5**	6.86	0.26
**tBu A**	1.13	0.07

3.8. Spectra of 1 and 2 in the racemic mixtures of PBLG-PBDG

All the spectra of compounds **1** and **2** in the racemic mixtures of PBLG-PBDG (1:1) in $CDCl_3$ or THF-d_8 were folded in the ^{13}C domain to measure smaller window, and, thus, obtain better resolution.

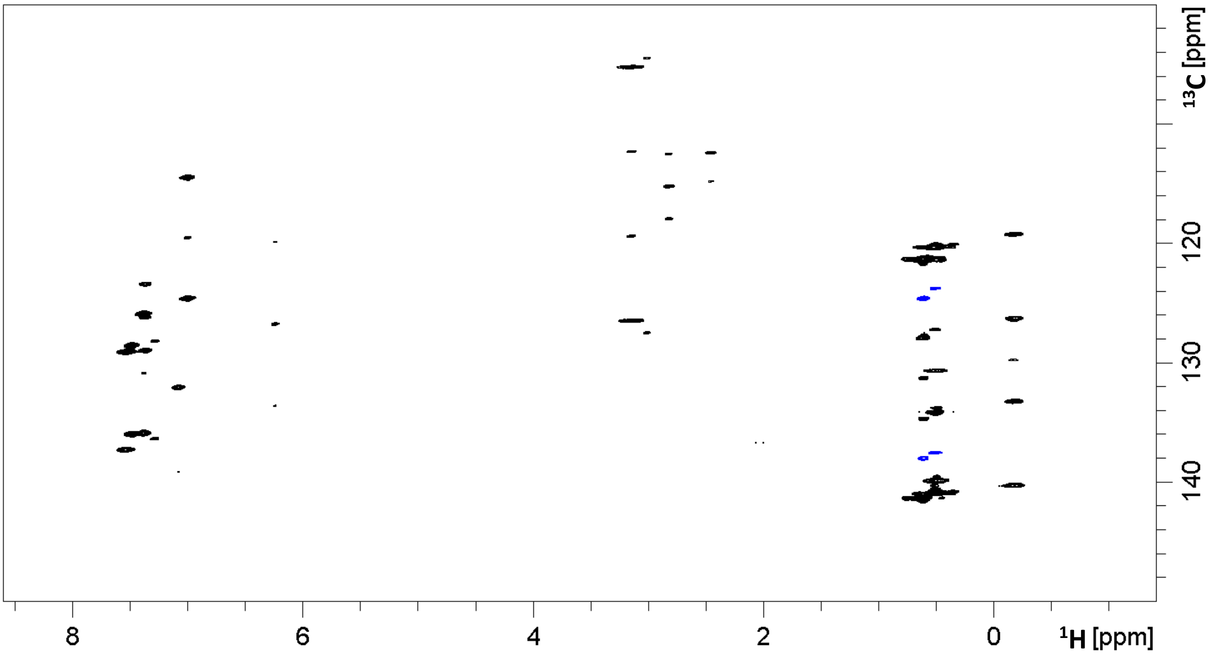

Figure S10: F1-coupled HSQC spectrum of **1** in the racemic mixture of PBLG-PBDG and $CDCl_3$ measured at room temperature and 600 MHz.

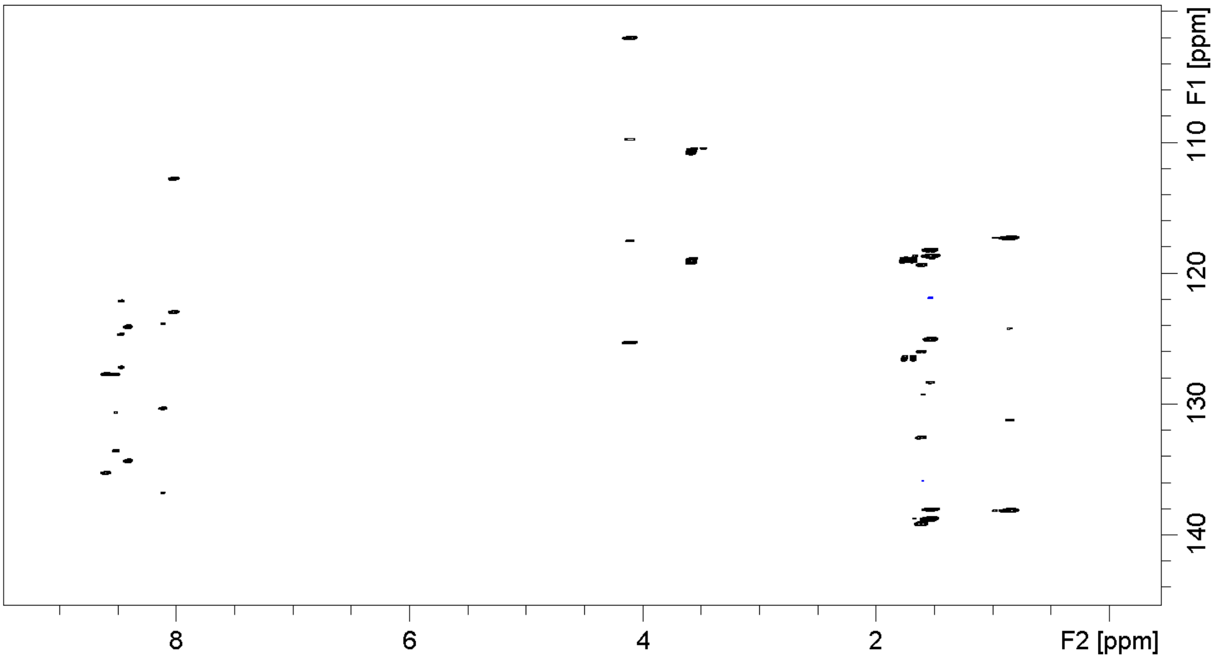

Figure S11: F1-coupled HSQC spectrum of **1** in the racemic mixture of PBLG-PBDG and THF-d_8 measured at room temperature and 600 MHz.

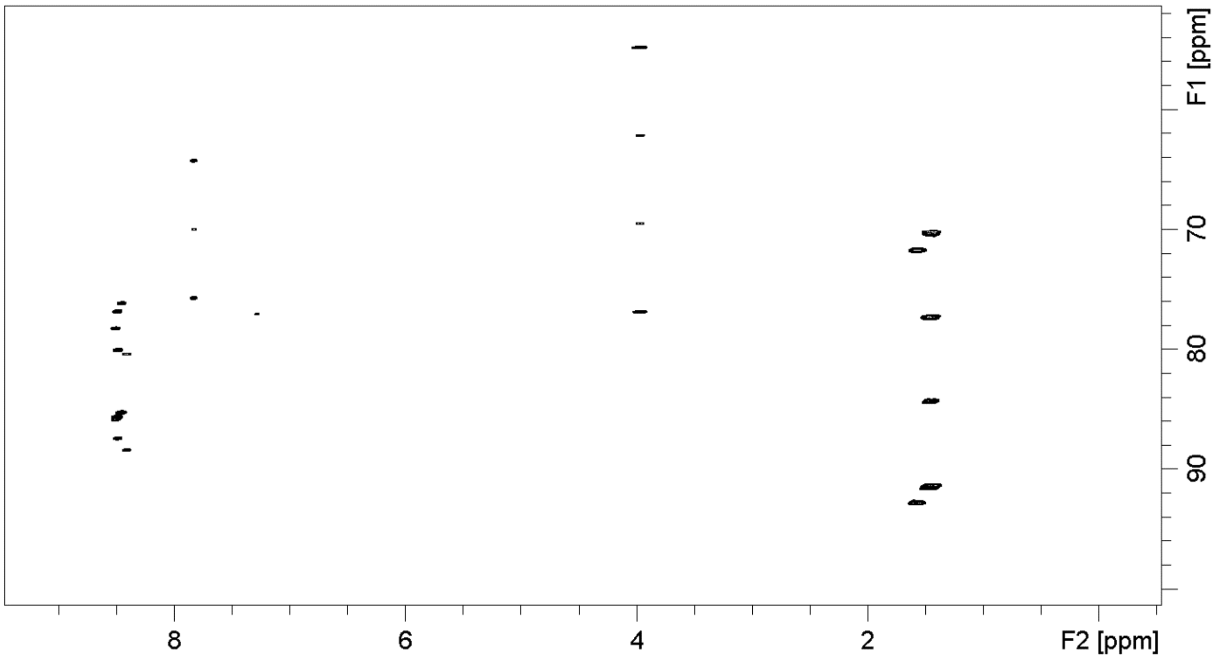

Figure S12: F1-coupled HSQC spectrum of **2** in the racemic mixture of PBLG-PBDG and $CDCl_3$ measured at room temperature and 600 MHz.

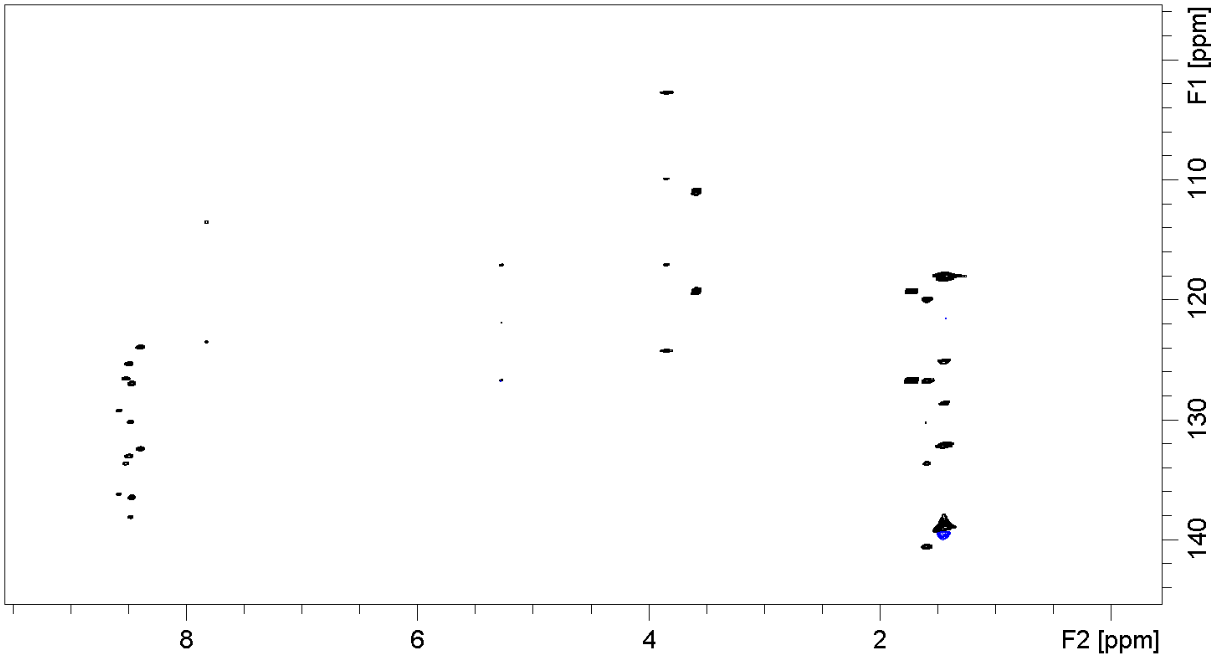

Figure S13: F1-coupled HSQC spectrum of **2** in the racemic mixture of PBLG-PBDG and THF-d_8 measured at room temperature and 600 MHz.

4. X-ray measurements

Crystallographic data for 2:

A slow evaporation of the solvent ($CHCl_3$/EtOH mixture) was used to crystallize the product from its nearly saturated solution at room temperature. The structure of **2** was measured using a D8 VENTURE equipped with a Photon CMOS detector with Cu-Ka (λ=1.54178 Å) radiation at 180 K. The structure was in a triclinic system, $P\,2_1/c$ space group with lattice parameters a=13.4176(3) Å, b=24.4722(6) Å, c=16.4261(4) Å, α=90° β=103.8661(11)° γ=90°, Z=2, V=2660.5(5) Å^3, D_c=1.296 g/cm^3 , μ(Cu-K α)=2.846 mm^{-1}. The data reduction and absorption correction were done with the Apex3 software. The structure was solved by charge-flipping methods using the Superflip software and refined by full matrix least squares on F squared value using Crystals software to the final values R=0.0579 and wR=0.1431 using 9581 independent reflections (Θ_{max}=68.244°), 680 parameters and 88 restraints. The MCE software was used for the visualization of residual electron density maps. According to common practice, the hydrogen atoms attached to carbon atoms were placed geometrically with U_{iso}(H) in the range 1.2–1.5 U_{eq} of the parent atom (C). The disordered functional groups were refined with restrained geometry and occupancy constrained to full for each atomic position. The crystal was partially solvated (0.487(2)) with chloroform. The structure was deposited into Cambridge Structural Database under number CCDC 2039755.

Empirical formula	C48 H63 Cl3 O13 S4
Molecular formula	C47.49 H62.49 Cl1.46 O13 S4
Formula weight	1021.40
Temperature	180 K
Wavelength	1.54178
Crystal system	monoclinic
Space group	$P\,2_1/c$
Unit cell dimensions	a = 13.4176(3) Å
	b = 24.4722(6) Å
	c = 16.4261(4) Å
	β = 103.8661(11)°
Volume	5236.5(2) Å^3
Z	4
Density	1.296 Mg/m^3
Absorption coefficient	2.846 mm^{-1}
F(000)	2160.974
Crystal size	0.151x0.303x0.324 mm^3
Crystal color, habit	colorless, plate
Theta range for data collection	3.308° to 68.244°
Index ranges	-16<=h<=16 -29<=k<=28 -19<=l<=19
Reflections collected	91509
Independent reflections	9581 [R(int) =0.042]
Completeness to theta = 68.244°	1
Absorption correction	multi-scan
Max. and min. transmission	0.42 and 0.65

Refinement method	Full-matrix least-squares on F^2
Data / restraints / parameters	9581/88/680
GoF on F2	0.91
Final R indices [I>2sigma(I)]	R1 = 0.0520, wR2 =0.1405
R indices (all data)	R1 = 0.0579, wR2 = 0.1431
Largest diff. peak and hole	-0.47 and 1.24 e.Å^{-3}

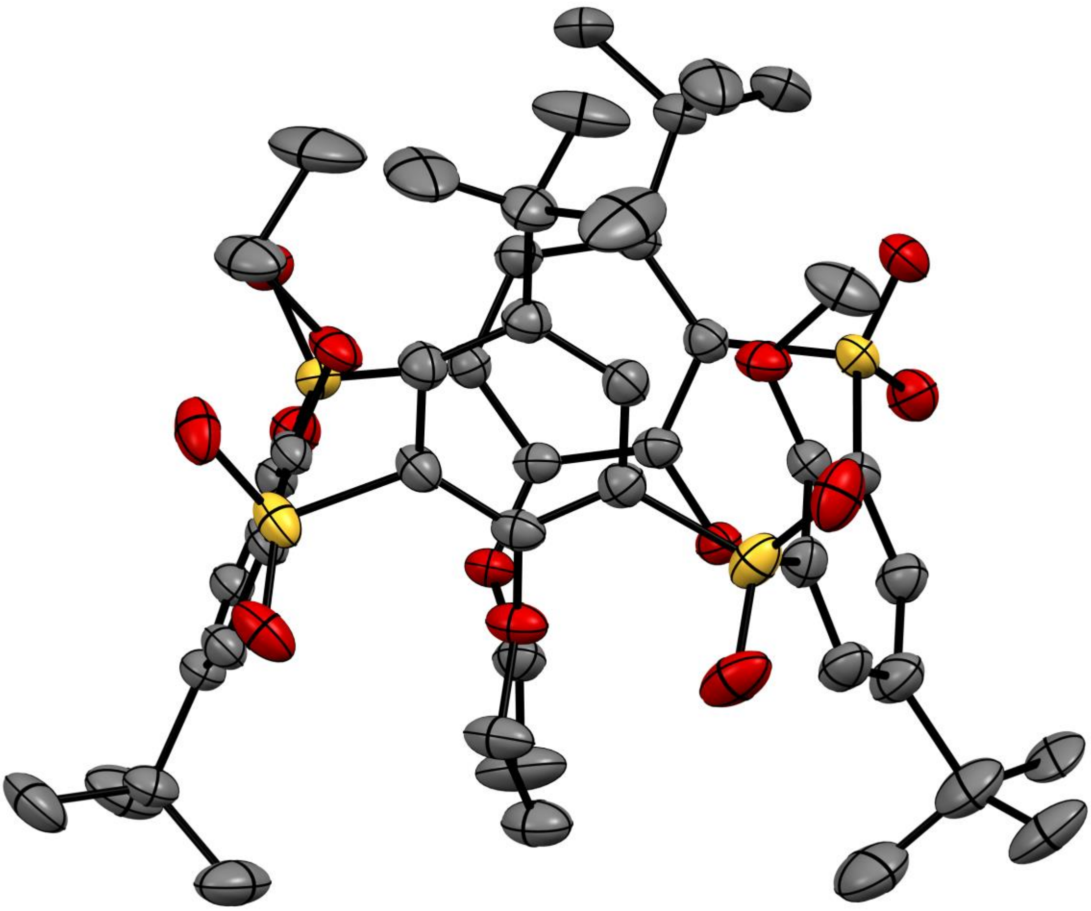

Figure S14. Thermal ellipsoid plot (50% probability level) of compound **2** (without solvent).

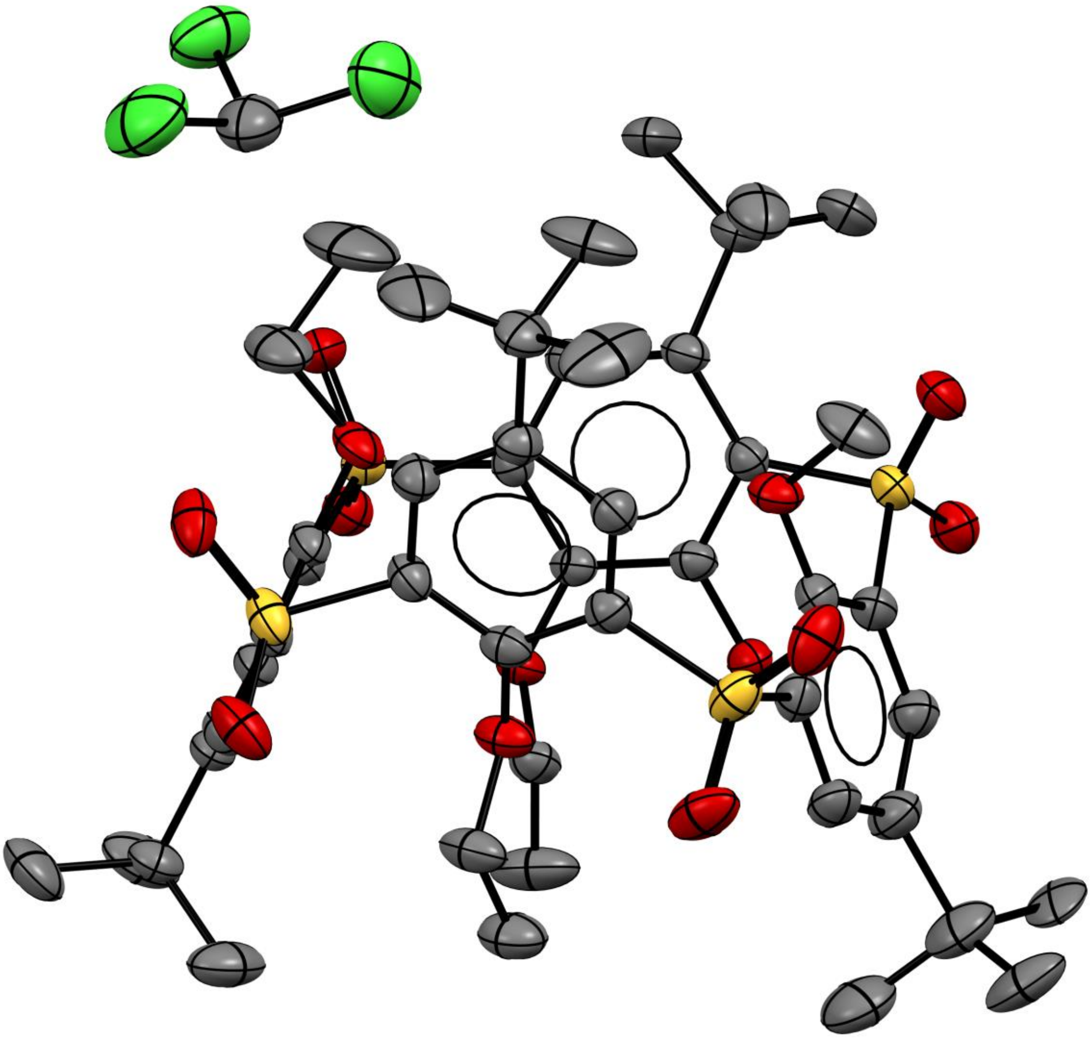

Figure S15. Thermal ellipsoid plot (50% probability level) of compound **2** (with solvent).

5. Theoretical Calculations

Gaussian 03[6] was used for DFT optimization of compound **1** (B3LYP[7]/6-31G*[8]) and *Orca*[9] for optimizations of compound **2** (B3LYP[7], def2-SVP[10] def2/J basis set, RIJCOSX[9] approximation, D3BJ[11] dispersion correction). Calculations were performed in vacuo.

Small imaginary frequencies (always max. -20 cm^{-1}) were found in the case of **2** (*1,2-alternateAB* with inverted rings A and B*, 1,3-alternate*, *cone*, *partial cone* with inverted ring A and *partial cone* with inverted ring D). These imaginary modes were found for the *tert*-butyl groups, sometimes with the ethoxy/methoxy groups present at the lower rim. The exact position of the rotation does not concern the stability of the optimized structure, nor the calculation of residual dipolar couplings, and, according to the literature, these could be neglected if the frequencies are less than tens of wavenumbers[9, 12].

5.1. XYZ coordinates of 1

1,2-altAB

atom	x	y	z
C	-3.48818	4.763495	1.447942
C	-3.70482	3.070706	-0.38617
C	1.655735	-2.24359	1.106391
C	-3.64825	0.73117	-1.05561
C	-0.64981	-2.21208	-1.50122
C	2.709841	2.564236	1.66988
C	1.837811	1.478138	1.541556
C	-4.58902	-2.91295	1.801493
C	-4.24536	-3.79738	0.576833
C	3.169	-2.81269	-0.75325
C	1.786263	3.556295	-0.30879
C	-2.40831	3.192912	-0.9356
C	-1.80412	-3.86806	-0.11497
C	-0.66105	-3.39982	-0.76338
C	-3.02832	-1.93549	-0.8599
C	2.687721	3.645074	0.76518
C	3.731786	-0.98879	0.702609
C	0.882131	1.406896	0.495287
C	2.016878	-3.03986	-0.00283
C	-5.77368	3.943601	0.809894
C	0.915113	2.463091	-0.43997
C	-4.50144	5.420007	-0.7767
C	2.549094	-1.20367	1.434075
C	-5.44021	-3.96315	-0.39254
C	0.572113	-1.64951	-3.46891
C	-3.91918	-5.20768	1.117872
C	-3.0328	-3.18398	-0.15836
C	-4.37356	4.288205	0.270336
C	4.062457	-1.77553	-0.41161
C	-1.73101	2.106692	-1.50478

Cone

atom	x	y	z
C	-4.23053	0.531766	2.544986
C	-0.18957	1.461529	-1.05396
C	-3.37714	1.240451	0.350466
C	-0.89518	2.493497	-0.42723
C	1.913543	2.53176	-0.28366
C	-3.73148	1.525627	1.683539
C	3.969805	0.682094	-0.65161
C	1.226479	1.52504	-1.00315
C	5.148369	0.080478	-0.19119
C	3.178262	-1.44874	-1.45311
C	4.362208	-2.05098	-1.0145
C	-1.57371	-2.45532	0.329804
C	-0.74073	-2.60252	-0.79983
C	-0.22528	3.514028	0.258087
C	0.618736	-2.23091	-0.62833
C	5.378607	-1.29861	-0.38305
C	2.98573	-0.06426	-1.30677
C	-3.52389	-0.05536	-0.18811
C	1.178489	3.577146	0.368198
C	-4.24136	-0.79261	2.052421
C	-3.87682	-1.0703	0.734294
C	0.720748	5.763665	1.605221
S	-2.70691	2.629654	-0.60801
C	0.238842	-1.50549	1.680301
C	-4.00929	-0.06049	4.991226
C	-4.74233	0.8317	3.96261
C	7.87946	-1.2515	-0.57365
C	0.784581	-0.83068	2.947745
S	-3.37653	-2.78301	0.350348
S	1.796873	-2.50351	-1.99744

C	-2.35885	0.848194	-1.58968	O	-3.26442	-0.37014	-1.47334
C	-4.31631	1.806277	-0.45459	O	-1.18057	-3.02264	-2.0085
C	-1.85319	-1.46626	-1.50477	C	-0.32767	-0.62524	3.99161
C	6.12777	-0.32458	-0.74103	C	1.797573	4.741301	1.177287
C	-1.0651	0.302471	1.313601	C	-4.40854	-0.55334	-2.32234
C	5.288991	-1.49275	-1.29034	C	-4.52904	2.307488	4.344969
O	0.45771	-2.43636	1.715444	C	-6.2582	0.5163	3.995389
S	-0.01587	2.416988	-2.00585	C	6.729295	-3.44513	-0.1587
C	3.652867	4.824594	0.954024	C	1.904037	-1.7095	3.552655
C	6.175545	-2.75753	-1.3581	C	-1.09049	-1.92166	1.539666
O	0.496533	-1.74299	-2.0366	C	6.681252	-1.93367	0.129198
S	2.194295	0.075986	2.664678	O	1.802863	0.477123	-1.68323
C	5.106532	4.299782	0.865747	S	3.73502	2.452737	-0.49394
S	0.880254	-4.32507	-0.58492	C	-1.27225	-4.44449	-2.19963
C	3.447287	5.905377	-0.12241	C	2.4259	4.176398	2.47642
C	4.78858	-1.12225	-2.70888	C	2.829507	5.519765	0.3258
C	0.463336	-3.22909	2.917873	C	-0.88432	0.268781	-3.02039
S	-4.46109	-0.85343	-1.22481	C	1.091193	-1.70765	0.576889
O	0.010691	0.375628	0.346619	C	1.356608	0.555859	2.566416
C	3.41207	5.453943	2.347062	C	6.776555	-1.71461	1.658152
O	-1.71899	-0.2317	-2.10432	O	-0.80165	0.391164	-1.58255
O	-4.79003	-1.09329	-2.64878	O	4.022495	3.091434	-1.79955
O	0.504972	1.306302	-2.82759	O	1.2755	-1.9821	-3.27649
O	0.002221	3.809853	-2.52639	O	2.26169	-3.91203	-1.89336
O	-5.54533	-0.85527	-0.2086	O	4.512839	2.844065	0.710756
O	1.01542	-0.27029	3.495872	O	-3.87118	-3.24671	-0.96432
O	3.479244	0.410382	3.331179	O	-3.64912	-3.57749	1.575404
O	0.67298	-5.32733	0.492571	O	-3.05696	2.472361	-2.03766
O	1.347455	-4.75231	-1.92705	O	-3.08001	3.86473	0.132272
H	-2.47445	5.055851	1.108904	H	-3.6239	2.568048	2.011408
H	-3.37303	3.965744	2.210279	H	5.87302	0.717469	0.336572
H	-3.9452	5.647683	1.938007	H	4.435404	-3.14124	-1.13171
H	3.439524	2.517574	2.492922	H	-0.85395	4.294119	0.702317
H	-3.72529	-2.87684	2.497556	H	-4.48816	-1.64891	2.698877
H	-4.85679	-1.8847	1.507324	H	0.196896	6.21385	0.738837
H	-5.45214	-3.3481	2.346646	H	1.215526	6.584883	2.160657
H	3.323595	-3.44437	-1.64047	H	-0.03882	5.324935	2.283379
H	1.727065	4.333779	-1.08276	H	-4.13677	-1.13999	4.772246
H	-1.87599	4.155137	-0.90594	H	-2.9234	0.157858	5.00742
H	-1.69915	-4.80141	0.449392	H	-4.41187	0.121553	6.008757
H	4.376412	-0.15684	1.014982	H	7.915712	-0.16301	-0.36519
H	-6.458	3.613927	0.0022	H	7.824784	-1.38673	-1.67315
H	-5.73333	3.14509	1.578678	H	8.833467	-1.69163	-0.21798
H	-6.22332	4.839028	1.283107	H	-0.76801	-1.58605	4.326286
H	-5.12245	5.098092	-1.63657	H	0.081756	-0.11645	4.886693
H	-3.51379	5.733215	-1.16967	H	-1.14462	0.007181	3.586931

H	-4.97837	6.312326	-0.322
H	-5.17169	-4.64726	-1.2222
H	-5.79003	-3.01038	-0.82195
H	-6.29959	-4.40677	0.151149
H	1.56705	-1.22457	-3.6874
H	-0.20054	-0.96508	-3.8678
H	0.488151	-2.66352	-3.91237
H	-3.6252	-5.91025	0.312621
H	-3.11597	-5.19281	1.881685
H	-4.8261	-5.61727	1.604533
H	-5.31483	1.614195	-0.04114
H	6.503744	-0.5321	0.281133
H	5.550242	0.622151	-0.71308
H	7.007814	-0.15683	-1.39334
H	-1.56167	-0.6666	1.13613
H	-1.77587	1.137962	1.14463
H	-0.66925	0.318595	2.345372
H	5.625165	-3.62383	-1.77562
H	6.540459	-3.04177	-0.35049
H	7.057086	-2.57578	-2.00626
H	5.315277	3.532696	1.63874
H	5.304948	3.847174	-0.12716
H	5.826309	5.131034	1.014507
H	2.417122	6.315969	-0.10336
H	3.645062	5.517868	-1.14275
H	4.145326	6.748663	0.052098
H	4.217948	-1.95296	-3.17053
H	4.126002	-0.23308	-2.6745
H	5.647174	-0.89391	-3.37343
H	0.860537	-4.24077	2.700247
H	1.04431	-2.71698	3.710467
H	-0.59335	-3.30381	3.230933
H	3.575114	4.723186	3.16471
H	2.375242	5.836976	2.436698
H	4.108319	6.3018	2.509229

H	-4.012	-0.9099	-3.28876
H	-5.09231	-1.32139	-1.90711
H	-4.92495	0.418529	-2.46369
H	-5.0718	2.993772	3.665038
H	-4.90716	2.487927	5.371063
H	-3.45534	2.584671	4.331484
H	-6.45645	-0.54855	3.758165
H	-6.8103	1.135769	3.260239
H	-6.67397	0.722336	5.00327
H	5.90808	-3.99057	0.348008
H	6.670587	-3.66204	-1.24447
H	7.684731	-3.86505	0.213788
H	2.742401	-1.84833	2.839375
H	1.519669	-2.71283	3.824247
H	2.316278	-1.23673	4.467707
H	-1.79326	-1.81562	2.374956
H	-0.2731	-4.9134	-2.07629
H	-1.62446	-4.59033	-3.23628
H	-2.01109	-4.88525	-1.49907
H	1.64149	3.709969	3.106369
H	3.209231	3.430122	2.26141
H	2.885097	5.003227	3.057074
H	2.363955	5.893867	-0.60753
H	3.71671	4.925149	0.053809
H	3.192745	6.394746	0.902093
H	-1.57893	1.041681	-3.40445
H	-1.27556	-0.74541	-3.20244
H	0.119364	0.348839	-3.479
H	2.150057	-1.43722	0.669543
H	2.199566	0.483037	1.849488
H	0.5773	1.194818	2.104048
H	1.740735	1.077386	3.466106
H	6.787387	-0.63848	1.923974
H	5.919957	-2.18651	2.181129
H	7.70955	-2.1657	2.053823

PaCoC

atom	x	y	z
C	-0.62829	3.882607	1.306553
C	1.400876	-2.16794	2.005696
C	-2.55275	4.690145	2.829568
C	-2.86201	3.112454	0.846158
C	-3.1841	-1.19338	-2.09984
C	-1.46433	5.551446	3.494803
C	-2.58148	0.050083	-1.72725
C	0.198951	-3.2888	0.265161

PaCoD

atom	x	y	z
C	-4.61009	1.44149	-0.31161
C	7.05175	-1.53509	-2.91251
C	3.1809	5.2981	0.12648
C	2.34204	4.56768	1.20362
C	2.2384	-1.16885	2.93979
C	1.09135	-1.63022	2.02899
S	-2.96147	-2.02495	2.1635
S	-2.66549	3.21499	0.47525

C	-0.11501	3.059305	0.294466	C	2.68831	0.22921	2.44599
C	-3.69668	5.612605	2.348889	C	6.00876	-3.72232	-2.25049
C	1.940221	-0.78438	-2.3553	C	4.72717	-0.16616	-1.44927
C	-1.17054	0.172265	-1.60903	C	0.33269	-2.20159	-0.24337
C	-3.09773	3.679129	3.868243	C	2.30328	-0.14376	-1.71493
C	-0.94712	-2.20094	-1.90677	C	-0.53892	1.67308	-0.54241
C	3.673114	1.611457	-0.68871	C	-2.27443	1.37122	3.56993
C	4.608842	0.578183	-0.47227	C	6.15638	-2.23881	-1.86481
C	-0.99647	-3.05747	0.969235	C	-1.26516	-1.97505	1.53088
C	-0.33264	-0.96617	-1.66284	C	-4.73709	-0.85243	0.40502
C	-5.26157	-0.31522	-3.29523	C	-5.81115	0.19309	-2.84457
C	0.211092	-1.89424	2.684659	O	1.09317	0.452	-1.62057
C	2.552798	1.764039	0.137691	C	-0.25028	-1.6694	2.44306
C	-4.68607	-1.43285	-2.3943	O	-2.0684	-2.37179	-0.68638
C	2.291724	0.888401	1.211123	C	-0.92204	2.74412	0.26848
C	4.344501	-0.31469	0.582089	O	-2.01092	0.85395	2.24731
C	3.212691	-0.16365	1.395013	C	-3.56491	1.65726	0.58968
C	-4.88329	-2.76181	-3.16285	C	-3.68998	-0.61715	1.30587
C	-1.9979	3.890633	1.641138	C	3.57781	-2.18975	-2.08565
C	-5.46067	-1.54838	-1.0571	C	1.40333	3.51811	0.55831
C	-1.02703	-2.32116	2.166295	C	3.42517	-2.15506	2.85503
C	-2.31697	-2.30052	-2.1599	C	-7.50836	0.978	-1.13653
C	-0.97567	2.233914	-0.44643	C	1.81078	2.43814	-0.29448
C	1.440025	-2.83613	0.761247	C	-2.28138	-3.68829	-1.22714
C	-2.3501	2.321768	-0.18777	S	3.47205	2.25982	-1.03853
C	3.249227	-4.19069	0.016226	C	-6.37396	-0.03373	-1.42025
C	1.288583	1.851071	3.144438	C	4.79622	-1.52562	-1.81785
C	6.677008	-0.79394	-1.02962	C	6.81776	-2.1517	-0.46766
C	5.860107	0.469563	-1.35919	C	1.78305	-1.05707	4.40631
O	0.966702	-0.86019	-1.3007	S	0.82322	-2.49429	-1.97563
S	-0.06279	-3.73959	-1.48054	C	-6.94678	-1.46068	-1.3387
C	-2.35933	-1.82666	2.750808	C	1.35474	-1.94784	0.68348
C	-2.21856	-1.44163	4.236577	C	3.49965	0.50936	-1.40668
S	1.632226	3.300418	-0.12386	C	1.53512	5.65925	1.94318
O	2.607092	-2.90765	0.079435	C	0.02276	3.61679	0.81711
O	1.163241	1.021314	1.967078	C	2.35375	-1.51689	-2.01408
S	2.922073	-1.44109	2.648805	C	-5.24202	0.18182	-0.40409
C	5.449986	0.415724	-2.84903	C	3.23695	3.87264	2.25916
C	-3.4649	-2.89255	2.612195	C	-1.82769	0.71328	-2.33026
C	6.739403	1.719155	-1.10883	C	0.84482	1.54126	-0.82001
O	-0.48135	1.33829	-1.34354	C	-3.09866	0.64887	1.45346
C	-2.74366	-0.56731	1.929388	C	-1.02436	-2.21351	0.16097
S	-3.47144	1.600278	-1.37855	O	-1.41959	0.73032	-0.94915
O	-3.43715	2.427985	-2.60622	O	4.59761	2.47394	-0.09116
O	1.708192	3.612495	-1.57106	O	-0.17405	-1.91601	-2.9024
O	2.154279	4.266425	0.882131	O	1.22369	-3.91978	-2.10934

O	-4.75525	1.389617	-0.65963	O	3.43063	3.0109	-2.31545
O	2.598576	-0.79915	3.949448	O	-3.63199	-3.25698	1.67813
O	4.032789	-2.42128	2.570004	O	-2.89953	-1.73649	3.62002
O	1.212086	-3.85057	-2.22461	O	-2.76249	3.97005	1.75137
O	-1.06706	-4.83054	-1.5339	O	-3.08903	3.85211	-0.79921
H	0.095384	4.515613	1.835954	H	-4.88011	2.27205	-0.98004
H	-3.95052	3.10383	1.004111	H	7.22076	-0.46949	-2.65953
H	-0.63298	4.935379	3.892883	H	6.59115	-1.57617	-3.9203
H	-1.03657	6.294599	2.791003	H	8.04327	-2.02907	-2.9654
H	-1.89823	6.110325	4.347532	H	2.52584	5.72172	-0.66052
H	-4.53776	5.03685	1.914109	H	3.93239	4.65231	-0.35507
H	-3.3389	6.325468	1.578644	H	3.73194	6.13507	0.60088
H	-4.10091	6.196589	3.200663	H	3.08359	0.19422	1.41086
H	2.907451	-1.01571	-1.88048	H	1.83948	0.94245	2.45234
H	1.728737	-1.54079	-3.13682	H	3.49328	0.63466	3.09196
H	1.95894	0.240581	-2.78029	H	5.38585	-4.2809	-1.52262
H	-3.91044	3.055783	3.444344	H	5.55925	-3.84557	-3.25671
H	-2.29549	2.995722	4.214036	H	7.00643	-4.20369	-2.27036
H	-3.50326	4.210388	4.75379	H	5.62771	0.39919	-1.16785
H	3.796063	2.328894	-1.51377	H	-1.28309	1.50005	4.04104
H	-1.93318	-3.39288	0.502776	H	-2.78171	2.35417	3.50031
H	-4.65998	-0.20408	-4.2191	H	-2.87155	0.63593	4.14529
H	-5.31434	0.665242	-2.79527	H	-5.11145	-1.882	0.33543
H	-6.29638	-0.58092	-3.59087	H	-4.99748	-0.52825	-3.06349
H	0.28311	-1.30532	3.607897	H	-5.40066	1.21497	-2.97151
H	4.996796	-1.1706	0.797974	H	-6.60846	0.05284	-3.60349
H	-4.29222	-2.79301	-4.10021	H	-0.55176	-1.45795	3.47709
H	-4.62894	-3.65379	-2.5565	H	3.53849	-3.26808	-2.29037
H	-5.95247	-2.85815	-3.43507	H	3.83076	-2.22944	1.82555
H	-5.37791	-0.62366	-0.46144	H	3.12663	-3.17246	3.1798
H	-6.53498	-1.73581	-1.2624	H	4.25351	-1.81139	3.50678
H	-5.07405	-2.4003	-0.46081	H	-7.91694	0.84019	-0.11445
H	-2.71127	-3.30814	-2.33986	H	-7.15827	2.02597	-1.2228
H	3.572905	-4.4955	1.032188	H	-8.33846	0.83982	-1.85998
H	4.133257	-4.05585	-0.6319	H	-2.6959	-4.34297	-0.43325
H	2.579161	-4.94444	-0.44338	H	-3.01227	-3.56927	-2.04635
H	0.258293	2.004717	3.514895	H	-1.34348	-4.1162	-1.63588
H	1.739548	2.828423	2.878959	H	6.18168	-2.63267	0.30345
H	1.898183	1.325379	3.905155	H	6.99588	-1.1029	-0.15677
H	7.052619	-0.78688	0.013029	H	7.7982	-2.66951	-0.47598
H	6.078155	-1.7171	-1.17259	H	1.40487	-2.02476	4.79318
H	7.557104	-0.85847	-1.6995	H	0.98249	-0.30015	4.5325
H	-1.84867	-2.29182	4.845037	H	2.63445	-0.74742	5.04506
H	-1.52583	-0.58876	4.382066	H	-7.34909	-1.68673	-0.33056
H	-3.20381	-1.13453	4.64059	H	-6.1841	-2.22743	-1.58504
H	4.86577	1.306338	-3.15578	H	-7.7772	-1.57355	-2.06422

H	4.839395	-0.48405	-3.06398	H	2.39675	-1.95577	0.33818
H	6.353358	0.373731	-3.49079	H	0.93626	5.25404	2.78317
H	-3.68491	-3.13205	1.552824	H	0.85732	6.21636	1.26503
H	-3.1869	-3.83487	3.125895	H	2.24719	6.38874	2.37717
H	-4.40827	-2.52105	3.059768	H	-0.36544	4.41658	1.45804
H	6.203645	2.653621	-1.3705	H	2.61235	3.4499	3.07196
H	7.0361	1.787395	-0.04308	H	3.84497	3.06547	1.8164
H	7.661709	1.67475	-1.72337	H	3.92882	4.61362	2.71056
H	-2.87943	-0.81799	0.858147	H	-2.55835	-0.11047	-2.40064
H	-1.94788	0.20328	1.992164	H	-2.30322	1.68238	-2.58842
H	-3.69231	-0.12506	2.296543	H	-0.97239	0.48957	-2.9952

5.2. XYZ coordinates of 2

1,2-altAB

atom	x	y	z
H	-5.74541	-3.7788	-2.38257
H	6.77155	-1.48596	1.93713
C	3.14378	-1.74183	-3.40604
H	-5.59977	-1.24336	-0.66827
H	5.14647	4.1073	-3.50564
C	-0.83174	4.14221	3.90245
H	-6.09544	0.8697	3.34419
H	-6.95965	3.43657	0.7122
H	7.60336	-1.29044	-1.12337
S	2.77011	1.70024	1.96318
C	-4.41239	1.45636	-0.3412
C	4.52934	4.03529	-2.59598
S	-1.32864	4.79164	-0.39927
H	6.19245	-3.16948	1.88079
C	6.69254	-2.37985	1.29893
H	3.4699	-3.44222	-0.26177
H	-4.25592	3.34771	3.3435
C	-4.02741	-4.29505	-0.41989
H	5.45715	-4.20898	-0.32901
C	-0.45506	-2.38832	-0.80414
H	-6.00176	3.30923	3.66413
C	-4.22968	-2.91121	-1.082
C	2.96084	0.06059	1.23919
H	5.07109	0.31791	1.17879
C	2.49799	4.29551	-4.04415
S	-3.74846	0.19961	-2.66708
H	3.78678	1.34729	-2.71945
C	-1.77886	-2.74242	-0.55412
C	1.02035	4.01762	-1.5829

1,2-altAD

atom	x	y	z
C	-4.47562	2.13956	0.02626
C	6.26422	0.31322	0.5083
C	1.24175	-3.68325	4.4792
C	0.65333	-3.1178	3.18038
S	-2.88401	-1.66611	1.92394
C	0.70779	0.80083	2.67342
S	-2.3156	3.75624	0.43904
C	1.65773	-5.15221	4.23749
C	4.40514	1.3116	1.89022
C	3.51844	1.27825	-1.61248
H	-1.42711	-1.93388	-2.3596
C	0.9209	-2.68112	0.80209
C	1.96416	-0.53002	-2.19868
C	-0.83507	2.42847	-3.10831
C	-0.77103	1.121	2.65435
C	5.01436	1.21657	0.47449
C	-1.13551	-2.10267	1.85378
C	-4.60066	-0.19038	0.51839
C	-6.63189	-0.08274	-1.76392
C	-0.63214	-2.57739	3.07466
O	-0.74389	-1.61317	-0.50173
C	-1.64214	2.82289	-2.04259
O	-1.20013	1.00608	1.27839
C	-3.16057	2.15786	0.49506
C	-3.29264	-0.16661	1.00503
C	3.4472	-0.66801	-0.24586
C	0.16201	3.90973	-0.83788
C	0.23032	-3.63974	5.633
C	-7.15277	2.24952	-1.01402

H	2.35476	1.57019	-3.75256
C	1.7265	-2.16489	-3.0979
H	0.59469	4.57726	-2.41309
H	3.14576	4.30374	-4.93409
C	2.08461	-2.01541	0.47574
H	3.70522	2.23712	-0.55107
H	3.97253	2.00229	-4.36368
C	-0.14558	-1.49235	-1.81244
C	2.27541	3.40261	-1.67359
C	3.32603	2.00641	-3.47109
O	-0.06722	3.20676	1.83136
C	-5.24777	3.40308	2.8678
H	-3.71142	-4.22931	0.63225
H	-0.20463	-1.55339	2.51436
H	-7.05674	1.68036	0.44603
C	1.96969	2.63091	0.63312
H	-5.58944	0.10056	1.82537
H	1.51944	3.89391	-4.34982
C	-4.39502	2.3818	0.71119
C	5.90786	-2.08037	0.00494
H	6.90776	-3.61831	-1.16181
C	3.37459	-2.44814	0.17136
C	-5.22761	-2.14881	-0.1848
H	1.70486	-3.14087	-2.59173
C	-1.60339	0.03904	2.02926
C	-2.54456	2.53322	-1.4811
C	-2.51523	3.43534	-0.40184
H	1.23091	3.79713	3.33705
C	-0.20974	-0.47405	2.31123
C	-1.20346	-0.79642	-2.43961
H	5.07482	3.40871	-1.87353
H	-1.94547	-3.49866	0.20696
H	2.35352	5.33887	-3.72444
C	-3.40527	3.36913	0.66887
C	-2.55212	-1.0756	-2.10185
O	0.58741	-0.2299	1.11334
C	1.82915	-0.73634	0.99543
H	1.11204	-2.2364	-4.01269
C	-3.51289	1.50825	-1.40955
C	4.25494	-0.36936	0.94984
C	4.4897	-1.63053	0.37927
H	5.29926	-3.1953	-1.78655
H	-4.36438	0.74957	2.94257
H	-7.62584	2.36996	1.98232
C	-4.81374	-3.19927	-2.48538
H	6.02489	-0.682	-1.68119

C	1.03017	3.53209	-1.89518
C	-1.7561	-2.21789	-1.35245
S	1.93771	1.83301	-3.76557
C	-6.64429	0.87465	-0.55327
C	3.9774	0.60633	-0.47757
C	5.42423	2.63199	0.03432
C	2.48734	-2.86146	4.87536
S	1.99403	-2.90975	-0.62679
C	-7.59034	0.3321	0.53787
C	1.4205	-3.15531	2.00862
C	2.51802	0.74777	-2.43155
C	-1.14744	3.46332	-0.90054
C	2.48207	-1.22847	-1.08714
C	-5.22001	0.95732	0.00597
C	-2.51665	1.00663	0.98032
C	-0.37392	-2.14099	0.67406
H	-4.88367	3.0805	-0.33964
H	6.70486	0.21126	-0.49546
H	6.0233	-0.69636	0.87569
H	7.02761	0.74094	1.1774
H	0.79631	-5.75952	3.91823
H	2.43431	-5.23883	3.46229
H	2.06658	-5.58984	5.16239
H	3.50913	1.94841	1.89389
H	4.1128	0.32544	2.28104
H	5.13704	1.74847	2.58846
H	3.93771	2.24017	-1.87703
H	-0.97761	2.14955	2.98564
H	-1.35521	0.4232	3.27412
H	-5.13042	-1.14311	0.55454
H	-6.29586	-1.09193	-1.47907
H	-5.95319	0.29044	-2.54726
H	-7.64086	-0.17237	-2.19715
H	-1.28577	-2.50202	3.94281
H	3.79568	-1.26624	0.5969
H	-0.66979	-4.2327	5.4091
H	-0.08882	-2.61188	5.86558
H	0.68798	-4.05972	6.54181
H	-7.19002	2.96904	-0.18178
H	-6.51978	2.67444	-1.80797
H	-8.17151	2.15495	-1.4206
C	-1.87216	-3.72001	-1.21641
H	-2.71509	-1.72061	-1.14012
H	4.56468	3.31888	0.01412
H	5.8901	2.63436	-0.96363
H	6.1639	3.03975	0.73967

C	-6.8531	2.45527	1.20072
H	0.41091	5.13094	2.43735
H	6.74738	-0.05098	-0.18193
C	-2.86295	-2.17889	-1.23986
C	2.72809	2.72034	-0.5377
C	6.60847	-0.95828	-0.78804
C	5.88286	-3.34784	-0.86569
S	0.78098	-3.21773	0.20008
C	-5.4493	2.29585	1.82371
H	-3.29959	4.11837	1.4511
O	1.12344	-1.17786	-2.21661
H	-5.15705	0.66399	-0.35135
H	-4.75777	-1.88718	0.77662
C	0.26928	3.94768	-0.41001
C	0.71143	3.2554	0.7333
H	-6.09227	-2.79581	0.02989
H	0.26626	0.07027	3.13745
H	-3.29692	-4.9113	-0.9655
H	-4.10688	-3.80267	-3.07636
H	4.43678	5.04314	-2.16159
H	-5.0495	-2.28256	-3.03117
H	-4.99105	-4.82474	-0.43304
C	-5.36555	0.92276	2.52102
C	3.14405	3.44453	-2.93973
C	0.28526	4.13507	2.88937
H	-5.35718	4.403	2.41749
H	7.71285	-2.72298	1.06045
O	-3.38011	0.66212	-4.00488
O	-1.49963	5.52715	0.86355
O	1.91875	1.59257	3.15408
O	-5.13442	-0.24662	-2.45863
O	-1.44548	5.53051	-1.66666
O	4.12595	2.25442	2.09497
O	0.14213	-3.52636	1.48876
O	1.36945	-4.32472	-0.57523
O	-1.63086	2.64398	-2.46438
O	-0.85171	0.13038	-3.3405
C	-2.02376	3.28689	-3.6923
H	0.1113	0.24493	-3.24507
H	-2.89818	2.78302	-4.12041
H	-1.17277	3.16242	-4.37414
H	-2.20611	4.35422	-3.50322
H	3.17501	-0.80052	-3.97332
H	3.64084	-2.52181	-4.00333
H	3.71176	-1.60508	-2.47516
H	-1.75679	4.52429	3.44807
H	2.22339	-1.80973	5.07087
H	3.25488	-2.88067	4.0855
H	2.94026	-3.27793	5.78951
H	-7.61861	1.00813	1.40674
H	-7.27815	-0.66056	0.8952
H	-8.6135	0.23754	0.14129
H	2.42705	-3.57212	2.00279
O	3.00216	2.85989	-3.94926
O	1.57824	1.03582	-4.93706
O	1.24855	-3.56676	-1.70485
O	3.21431	-3.54985	-0.10765
O	-3.63022	-2.76282	1.28117
O	-3.16926	-1.33841	3.33265
O	-1.61284	3.96628	1.71063
O	-3.30542	4.73762	-0.02981
O	0.98571	-1.02474	-2.97799
C	1.38457	-1.86321	-4.07879
H	1.93653	-1.26574	-4.81648
H	0.45573	-2.23929	-4.52435
O	0.6351	4.68948	0.15137
C	0.56388	2.68984	-2.94288
C	-1.54078	1.88274	-4.37864
O	2.28212	3.99554	-1.7824
C	-1.40161	0.35568	-4.50577
C	-3.05558	2.18532	-4.33474
H	-3.57874	1.62383	-3.54581
H	-3.49856	1.87244	-5.29195
H	-3.26663	3.25711	-4.19993
H	-0.36743	0.05657	-4.67314
H	-1.52928	2.23667	-6.5225
H	-1.76523	-0.13776	-3.59262
C	-0.9913	2.59922	-5.63192
H	0.07425	2.40167	-5.78828
H	-1.14982	3.68708	-5.55955
H	-2.0104	-0.00136	-5.35197
C	1.49738	4.05762	1.11065
H	2.76268	3.81202	-2.63011
H	0.85766	3.64138	1.90343
C	2.46443	5.082	1.65754
H	2.03507	3.22849	0.62177
H	-2.7063	2.61284	-2.08036
H	1.97455	-2.71258	-3.71293
H	-2.22875	-4.00392	-0.21725
H	-0.91271	-4.20533	-1.43413
H	-2.62075	-4.07589	-1.94134
H	1.23566	1.42451	1.93944

H	-0.55785	4.79831	4.74303	H	0.89365	-0.25081	2.41579
H	-1.00642	3.12983	4.2975	H	1.13066	1.0015	3.67071
H	-2.04217	-0.46361	1.15466	H	1.92222	5.89689	2.16076
H	-1.59109	1.12427	1.85034	H	3.07023	5.51183	0.84547
H	-2.24819	-0.16783	2.89782	H	3.13795	4.60681	2.38871

1,3-alt

atom	x	y	z
H	17.93088	2.2032	8.80939
H	13.70177	3.21584	8.92369
C	16.61142	8.61192	5.74661
C	17.61578	8.6823	6.71691
H	14.43117	2.31212	10.27661
S	14.96812	7.49283	3.85433
C	13.68994	6.54352	4.70236
O	17.12194	8.20303	10.72139
S	10.75851	6.91121	7.49401
O	16.80105	5.13479	4.55495
C	11.96384	6.2636	6.32529
C	12.72382	4.33023	5.05639
C	16.8631	6.94384	10.32945
C	11.91681	4.89974	6.05245
C	16.31614	7.43851	5.0503
C	12.81823	7.14065	5.63063
C	18.08023	6.29166	6.20776
O	18.77084	5.14508	6.30663
C	17.95084	8.35805	11.88091
C	17.72816	4.80191	3.49834
C	17.0596	6.27877	5.2267
C	15.688	6.27641	10.7218
C	12.5082	7.06407	9.68213
C	13.43658	9.21867	10.38146
C	15.37286	4.99507	10.26043
C	16.2279	4.30975	9.39387
C	11.76822	7.77072	8.71951
S	14.4321	7.07891	11.74721
H	16.68375	0.94681	8.64353
C	13.63233	5.17668	4.40924
S	19.11274	7.04732	8.59927
C	18.27088	7.44439	7.02183
C	12.66362	9.91856	9.4438
C	11.83302	9.16233	8.61308
C	17.42553	4.94346	9.04094
C	13.35933	7.83306	10.50267
C	17.72575	6.24239	9.45749
O	12.42307	5.72044	9.79434

Cone

atom	x	y	z
C	2.53701	4.53118	1.02834
H	-4.48311	3.46923	5.05759
C	-5.41231	0.93831	4.52844
H	7.16354	-2.84844	-0.82347
H	-0.17343	-4.92886	-1.35064
C	-2.86743	3.37256	-5.2436
H	5.8197	-3.01033	1.40259
C	-6.12284	2.7496	2.96741
H	-2.3948	4.29993	-3.34128
C	3.01719	2.06648	1.44135
H	4.67943	-2.90339	-0.39209
H	-5.82892	3.55353	2.27605
H	3.4622	-3.42429	4.8627
H	-0.8587	-2.22664	3.39184
H	8.52763	-2.20424	0.12468
O	-0.98818	-3.03758	-1.06041
C	6.26409	-2.04986	1.70405
C	-1.15314	-4.42429	-1.4118
H	3.30971	2.18792	2.49643
C	5.36671	-0.83952	-0.29959
H	3.58351	-3.54267	3.09357
H	-0.68773	2.72302	0.71172
H	0.37492	-1.33355	5.05
S	2.10211	-2.97493	-1.39691
C	-0.16975	2.74636	-0.24153
H	2.11194	-0.07712	3.71783
H	0.42516	-3.11392	5.15488
C	0.93743	3.09293	2.26629
H	-6.07308	0.19827	4.04973
O	-1.01885	2.80346	-3.83844
H	-1.8402	-4.88017	-0.67971
C	-3.4799	-1.03541	-2.37925
C	-0.37803	2.84411	-2.63887
H	3.02402	4.77141	1.98811
H	3.41323	-0.87067	4.64701
C	4.47027	-1.86211	-0.63846
H	2.28937	-4.4138	3.95246
C	3.25574	-1.5857	-1.26256

C	11.35768	5.24851	10.6742	H	3.28697	4.61918	0.23481
O	12.80633	8.46164	5.88967	H	1.74166	5.27286	0.85267
C	12.05344	9.38122	5.03536	H	2.63932	1.04071	1.31554
O	15.33729	6.74658	2.64474	H	-3.56865	3.72486	3.55125
O	14.5771	8.90407	3.73453	H	8.18857	-0.15627	1.63051
O	20.10492	5.98206	8.27772	C	2.87408	-3.4802	3.93252
O	19.60999	8.19909	9.34868	C	1.04419	-2.20825	5.0577
O	15.02532	8.18013	12.51536	H	2.87451	-2.53215	1.26668
O	13.7081	6.01287	12.45095	H	-3.98582	3.14009	1.19291
O	9.95815	7.92761	6.79376	O	-1.79725	-0.38164	-0.70505
O	10.07477	5.76032	8.10433	H	-6.67999	3.21235	3.79788
H	17.58705	11.09603	5.30623	H	7.90904	-1.30032	-1.28452
C	15.8683	2.96622	8.74835	C	1.93571	3.10683	1.08929
C	14.47948	2.4859	9.19022	H	1.49004	3.32749	3.18794
C	16.91977	1.90712	9.1297	H	3.41942	-0.92635	2.86794
C	15.85208	3.16344	7.21598	C	3.81696	0.73856	-1.32958
H	16.03378	9.49058	5.47868	H	-6.807	2.07499	2.42944
C	12.76082	11.44601	9.36191	C	1.22434	2.84228	-0.26839
C	12.20998	12.04409	10.67329	C	7.61543	-1.92576	-0.42718
C	11.96816	12.00202	8.16992	C	1.93998	-2.25734	3.81148
C	14.23757	11.85404	9.19193	H	-4.05371	-0.71068	3.16375
C	12.52561	2.86913	4.63643	H	-4.58643	0.39689	5.01381
C	12.29899	1.9656	5.86144	H	-1.32011	-1.29682	-2.42104
C	11.27421	2.81894	3.732	C	1.78515	-2.54248	1.29038
C	13.73797	2.33955	3.85435	C	-0.31337	-2.77271	0.06498
H	18.11628	4.43547	8.37441	C	-3.78786	-0.04065	2.34707
H	14.42923	4.56608	10.5811	C	-0.27368	-2.3616	2.48359
H	14.1218	9.73868	11.05085	C	-0.97176	-2.53021	1.28538
H	11.21302	9.62914	7.85283	C	6.63768	-1.16816	0.49211
H	11.20237	4.30208	6.618	C	-3.06255	-0.59371	1.28751
H	14.31776	4.80443	3.65101	C	2.7705	-0.95908	3.75555
H	18.75384	4.97104	3.86171	C	2.89788	-0.27638	-1.64225
C	17.52557	3.35186	3.12327	C	-3.95503	2.95561	4.23765
H	17.52331	5.47834	2.6532	H	-3.09187	2.42214	4.66662
H	10.43728	5.78339	10.40306	H	5.67286	1.30403	-0.45146
C	11.1852	3.76427	10.47164	C	-4.90561	1.97574	3.51688
H	11.65566	5.49296	11.70365	S	-2.7504	2.65389	-1.18145
H	12.78067	10.1326	4.70435	C	-3.02826	1.52815	0.19407
H	11.30407	9.84445	5.69217	C	-4.14433	1.31286	2.35975
C	11.36644	8.73499	3.85154	H	6.62687	0.69877	1.64034
H	20.10873	9.51175	7.64026	H	7.67006	0.74987	0.19437
H	17.71612	11.42266	8.91665	C	-2.06789	-0.55333	-2.12865
H	19.832	10.18048	6.00745	C	-1.88243	3.90351	-4.2295
H	16.52229	10.10195	8.8269	C	-0.96096	2.6959	-1.39384
H	17.76252	12.1663	6.7151	C	5.0212	0.46139	-0.67784
H	16.27118	11.23414	6.50633	H	-1.24326	4.7033	-4.64478

H	18.16206	9.77118	9.40072
H	14.23625	1.53495	8.6914
H	16.94094	1.74531	10.21896
H	15.59999	2.21431	6.71729
H	15.09907	3.91108	6.92252
H	16.82031	3.50103	6.8236
C	17.58268	10.35272	8.687
C	18.01969	10.0906	7.23248
C	19.54616	10.25539	7.06867
C	17.36383	11.19731	6.37922
H	19.85072	11.2492	7.43569
H	12.29279	13.14327	10.66106
H	11.15011	11.7776	10.81326
H	12.76992	11.67207	11.54513
H	12.08539	13.09599	8.12212
H	12.33146	11.58299	7.2191
H	10.89082	11.78727	8.24932
H	14.32642	12.95131	9.14111
H	14.65305	11.42892	8.26594
H	14.86396	11.51155	10.02832
H	12.16031	0.92386	5.53098
H	13.15841	2.00225	6.54613
H	11.40016	2.24505	6.43212
H	11.08129	1.78799	3.39337
H	11.40656	3.45389	2.84235
H	10.38313	3.17566	4.27208
H	13.58499	1.27943	3.60016
H	13.88645	2.88233	2.90913
H	14.66358	2.42346	4.44261
H	19.50379	5.27079	6.96594
H	18.2168	9.42109	11.92957
H	18.87091	7.76523	11.77447
H	17.38257	8.06937	12.77638
H	18.2326	3.06863	2.32852
H	16.50445	3.17525	2.75316
H	17.70197	2.70094	3.99317
H	10.92119	3.54708	9.42672
H	10.37059	3.40353	11.11828
H	12.09681	3.21106	10.74067
H	10.63933	7.97801	4.17819
H	12.0893	8.28657	3.15657
H	10.81781	9.51753	3.30294

C	-2.63984	0.16791	0.17927
H	5.5417	-1.5339	2.3545
C	1.03709	2.93997	-2.72404
H	3.9196	2.19568	0.84297
C	1.83979	2.83815	-1.56612
H	-1.86487	0.38905	-2.64939
C	1.09226	-2.72235	0.09132
C	-1.71085	-4.47753	-2.81458
H	-5.98811	1.44243	5.32026
C	7.31547	0.10802	1.01487
C	-3.74922	2.07743	1.25425
S	3.61227	2.46993	-1.86664
H	7.16063	-2.27756	2.30186
C	1.12485	-2.38631	2.51657
H	0.46546	2.10655	2.40362
H	1.67447	-2.13415	5.95794
H	0.14608	3.84972	2.16377
S	-2.76671	-2.38296	1.39528
O	3.89969	2.45665	-3.30297
O	2.8851	-4.1963	-1.15245
O	-3.43022	-3.06377	0.27835
O	4.47301	3.30044	-1.00462
O	1.30413	-2.86252	-2.62199
O	-3.12224	-2.76325	2.77397
O	-3.40358	2.09598	-2.37529
O	-3.14148	4.00164	-0.73462
O	1.70404	-0.01361	-2.21666
O	1.52157	3.08933	-3.96177
H	0.74644	2.99884	-4.54908
C	1.67037	-0.07937	-3.65448
H	0.64762	0.19424	-3.94093
H	1.88065	-1.10527	-3.9851
H	2.38567	0.63756	-4.07955
H	-2.34166	2.89309	-6.08437
H	-3.4792	4.19663	-5.64238
H	-3.52388	2.63642	-4.76096
H	-0.99609	-4.02549	-3.51662
H	-2.66332	-3.93045	-2.86801
H	-1.89161	-5.52268	-3.11036
H	-3.60175	-1.25958	-3.45097
H	-4.2106	-0.25472	-2.12289
H	-3.68792	-1.94088	-1.79314

PaCoA

atom	x	y	z
H	-6.92836	2.30781	4.44636

PaCoB

atom	x	y	z
C	0.40546	6.24109	1.49351

C	-4.90875	0.59613	5.19598	H	-2.49901	3.48265	6.40293
H	8.02106	-1.15641	-1.5024	C	-0.61503	3.20211	4.398
C	-1.20723	-5.0796	-2.46258	C	-0.91521	-0.6139	-2.2688
H	7.43108	-3.18556	-0.07389	H	-3.74153	3.87266	-3.02483
C	-6.82975	-0.33354	3.85427	C	-1.28229	1.14153	5.64256
H	5.07106	-3.17736	0.04001	H	-2.43466	3.9003	-4.25797
H	-7.3605	-0.32875	2.88937	C	2.11394	6.23414	-0.28723
H	2.88183	0.25948	4.533	H	-2.08894	0.45561	5.94349
H	-0.87405	-2.22427	3.63727	H	1.82482	-4.7131	6.23006
H	8.99601	-0.53248	-0.14427	H	-1.77023	-3.60044	3.56668
O	-0.73102	-3.56785	-0.69094	O	-0.46359	-1.08398	0.03967
C	7.386	-2.52353	0.80434	C	-0.74921	-1.70563	-1.24369
C	-0.79784	-4.97147	-1.01355	H	2.71489	6.91092	0.34188
C	5.56632	-1.06699	-0.21156	C	2.37453	0.65882	-0.72498
H	1.4858	0.45491	3.45059	H	1.01353	-3.16499	5.87623
O	-0.45715	1.23186	1.48644	H	-1.38032	4.87439	0.87936
H	2.78693	-3.50998	4.66572	H	0.9059	-6.40151	2.96752
S	2.4001	-3.54889	-0.78872	S	2.65331	-1.96937	0.43129
C	-0.21385	1.65559	0.21119	C	-0.9846	4.34	0.02213
H	0.5692	-2.53159	5.57711	H	-1.21494	-5.7583	4.28381
H	3.82806	-2.51542	3.61536	H	2.44372	-5.57085	3.28765
H	-4.53272	-0.41727	5.40715	C	2.13639	4.48342	1.59621
H	0.19182	-5.42744	-0.8478	H	0.18397	2.65231	3.87828
H	-2.1126	-0.13246	-2.1906	O	-2.30222	2.38707	-2.83489
C	-0.78646	2.46749	-1.99073	H	0.08851	-2.37757	-1.49212
H	1.47013	-1.0955	6.1235	H	-5.15944	-0.69695	-0.05961
C	4.75296	-2.20032	-0.3228	C	-1.50419	3.02024	-1.93013
H	3.02162	-0.20669	2.82329	H	1.07381	6.91933	2.04609
C	3.47033	-2.10449	-0.86879	H	-0.28765	-5.99663	5.78184
H	-6.71206	2.19843	2.68734	C	2.4219	-0.75136	-0.88133
H	7.94043	-0.20593	2.16129	H	2.50487	-3.60613	5.01235
C	2.34029	-0.19572	3.68816	H	-0.28451	6.85681	0.89607
C	3.08402	-2.477	4.42649	H	-0.17939	5.68686	2.24325
H	2.95899	-2.43779	1.68817	H	2.80891	5.68691	-0.93108
H	-4.60086	2.20862	1.73258	H	-3.74744	2.40658	5.71657
O	-1.94706	-0.91622	-0.26552	C	1.56658	-4.00503	5.42701
H	-7.54869	-0.06424	4.64393	C	1.49751	-5.89434	3.74565
H	7.75074	0.49259	-0.89272	H	2.44539	-3.43834	2.63837
H	0.04957	-0.90432	5.08116	H	-2.86549	3.67355	2.62077
C	3.76175	0.23444	-1.21673	O	-3.42507	0.38789	-0.43672
H	-6.49109	-1.36268	4.05022	H	-0.84891	1.57268	6.55772
C	1.1357	1.65876	-0.19321	C	1.27063	5.30865	0.61935
C	7.98929	-0.53868	-0.59163	H	2.77683	5.16177	2.18413
C	1.8587	-1.61884	4.0513	H	-1.14135	-4.4636	5.50887
H	-4.36286	-1.7624	3.43059	C	2.67349	0.82854	-3.16796
H	-4.05021	1.28461	5.21082	H	-0.49333	0.55294	5.14826

C	-3.67448	-1.61196	-1.89494	C	0.3873	4.38447	-0.25745
C	1.87579	-2.55853	1.68733	C	0.71318	-4.7094	4.35163
C	-0.12945	-3.20177	0.4478	H	-2.11839	-0.39109	3.84798
C	-4.12914	-1.01337	2.67301	H	-0.92228	4.03375	3.74743
C	-0.24082	-2.42581	2.77441	C	-4.61819	-0.75921	-2.1536
C	-0.85788	-2.90202	1.6145	C	1.40455	-3.17662	2.44228
C	6.96423	-1.0977	0.41687	C	-0.17298	-1.93349	1.0564
C	-3.24691	-1.38528	1.66308	C	-2.41262	0.3536	3.10975
C	0.92886	-1.53633	5.27219	C	-0.9215	-3.28003	2.96559
C	2.94926	-0.89922	-1.3541	C	-1.18193	-2.40194	1.91523
C	-6.19836	2.0835	3.65435	C	-2.83442	-0.1036	1.85453
H	-5.40089	2.8409	3.71388	C	-0.56047	-5.25723	5.01408
H	5.58875	1.08482	-0.54425	C	2.65767	-0.55919	-3.32457
C	-5.64987	0.66137	3.84302	C	-2.89531	3.05732	5.46737
S	-2.94877	2.09974	-0.43801	H	-3.27449	3.89163	4.85752
C	-3.37042	0.79645	0.72308	C	-1.79663	2.2653	4.73169
C	-4.67948	0.27616	2.71956	S	-3.55832	3.45517	-0.03708
H	6.22113	-0.61005	2.41998	C	-3.20924	2.15293	1.17203
H	6.66464	0.8196	1.45937	C	-2.35941	1.71474	3.41595
C	-2.26948	-1.09088	-1.67643	C	-4.75284	-0.03167	-0.83495
C	-1.17087	2.01082	-0.73074	C	-3.11172	3.24992	-3.67467
C	5.0311	0.15368	-0.65484	C	-1.91079	3.63645	-0.75571
C	-2.85756	-0.5078	0.63472	C	2.52617	1.408	-1.91194
H	6.70012	-2.97127	1.5395	C	-3.96382	2.37875	-4.56708
C	0.54915	2.58858	-2.39747	C	-3.19823	0.78396	0.82505
C	1.51752	2.07128	-1.48616	C	-0.12263	3.01855	-2.23303
H	-1.51389	-1.81218	-2.00648	H	1.45367	6.85315	-0.91735
C	1.26518	-3.04217	0.53051	C	0.82698	3.60319	-1.36736
H	-1.53297	-5.43815	-0.33927	H	-5.37324	0.87219	-0.92088
H	-5.58923	0.88186	6.01481	C	1.13827	-2.31644	1.37085
C	6.9458	-0.22007	1.68743	H	-1.66549	-2.30166	-1.12991
C	-4.26512	1.17364	1.73097	H	-0.18909	3.63369	5.31782
S	3.30148	1.87911	-1.81511	C	-2.82053	2.6006	2.43126
H	8.3878	-2.50368	1.26082	S	2.55564	3.21551	-1.86576
C	1.14249	-2.21669	2.83004	C	0.3832	-3.70767	3.23852
H	3.58411	-2.04773	5.30926	H	2.7767	3.76121	1.083
S	-2.65052	-3.0847	1.71154	H	1.73725	-6.62983	4.52967
O	3.55931	1.83086	-3.26513	H	1.47634	3.95383	2.30266
O	3.18548	-4.65513	-0.22071	S	-2.902	-1.90174	1.70908
O	-3.18278	-3.8169	0.55665	O	2.72119	3.69426	-3.24401
O	4.03637	2.85052	-0.99412	O	3.63103	-1.42826	1.38974
O	1.72219	-3.73827	-2.07589	O	-3.37861	-2.31999	0.38545
O	-2.94278	-3.56525	3.07357	O	3.55307	3.61525	-0.86715
O	-3.60442	1.83766	-1.7296	O	2.94178	-3.25411	-0.22904
O	-3.22111	3.37807	0.24702	O	-3.6088	-2.37295	2.9104
O	1.67601	-0.82491	-1.80635	O	-4.53464	2.99967	-1.03461

C	1.49879	-0.91059	-3.23083
O	2.04848	1.22076	0.69304
H	1.55567	0.86712	1.45543
C	-0.92305	2.23551	2.42218
C	-1.30811	1.54218	3.71007
H	-1.77521	2.76976	1.9799
H	-0.11335	2.96939	2.58032
C	0.80144	3.36058	-3.72364
H	-1.58581	2.75998	-2.66385
C	-0.46796	4.13094	-4.15706
C	1.15149	2.42055	-4.89634
C	1.88702	4.43428	-3.49423
H	1.57658	5.12442	-2.69389
H	2.01919	5.02196	-4.41605
H	2.86158	4.01325	-3.23198
H	0.3457	1.68451	-5.05046
H	2.09697	1.89614	-4.73752
H	1.23907	3.01684	-5.81913
H	-1.28649	3.46456	-4.46733
H	-0.21416	4.75157	-5.0288
H	-0.84273	4.79912	-3.36716
H	0.4409	-0.69086	-3.41845
H	1.73915	-1.92803	-3.56963
H	2.12833	-0.16802	-3.73899
H	-2.02074	0.72859	3.51333
H	-1.78505	2.26424	4.39009
H	-0.42982	1.12378	4.22183
H	-0.4645	-4.57197	-3.09329
H	-2.1952	-4.61958	-2.61933
H	-1.26154	-6.13773	-2.7624
H	-3.80119	-1.87084	-2.95842
H	-4.42401	-0.84452	-1.64958
H	-3.84938	-2.51235	-1.28938

PaCoC

atom	x	y	z
C	-0.20519	5.17591	-0.01285
C	0.39164	-1.3903	1.86764
C	-2.26715	6.5841	0.63417
C	-2.20557	4.89083	-1.26544
C	-3.12647	0.64733	-1.85812
C	-1.3549	7.16574	1.72436
C	-2.41999	1.3191	-2.91771
C	-0.87881	-2.49184	0.172
C	0.46807	4.23808	-0.81117
C	-2.74727	7.73286	-0.277

O	-3.82255	4.67837	0.73348
O	0.23146	2.38898	-3.36593
H	-0.5881	1.97996	-3.7035
C	2.73135	-1.25754	-4.68706
O	2.21262	1.3786	0.3883
C	1.57142	0.91105	1.5705
C	1.44729	-2.09086	-4.88575
C	3.97169	-2.17221	-4.72034
C	2.82856	-0.24886	-5.84065
H	1.32202	-2.85261	-4.10102
H	1.46914	-2.61036	-5.85735
H	0.56061	-1.43787	-4.86769
H	1.9595	0.42779	-5.86694
H	2.86099	-0.78983	-6.79971
H	3.73822	0.36768	-5.77334
H	3.92932	-2.94975	-3.94258
H	4.88895	-1.58316	-4.56083
H	4.05216	-2.67969	-5.69531
H	2.76423	1.50603	-4.01467
C	2.55997	-1.31808	-2.15525
H	2.60921	-2.40556	-2.20295
H	1.48919	1.79279	2.21511
H	2.17572	0.14814	2.07578
H	0.56701	0.53782	1.33059
H	-4.57419	3.01183	-5.23035
H	-4.63926	1.76068	-3.95867
H	-3.34618	1.71602	-5.19475
H	-1.20417	-1.0664	-3.23047
H	0.02077	-0.05926	-2.41535
H	-1.70972	0.07735	-1.96328
H	-4.12609	-0.11352	-2.89446
H	-5.61329	-1.0379	-2.53466
H	-4.02403	-1.67427	-2.02551

PaCoD

atom	x	y	z
C	-4.61024	1.19203	0.49581
C	6.88245	-1.19817	-0.61813
C	2.65742	5.7961	0.12209
C	2.18852	4.57296	0.94526
C	2.04824	-4.19768	3.11456
H	-3.80366	3.8225	-1.4593
C	1.11025	-3.69667	2.00854
S	-2.80563	-2.76391	1.7644
H	-0.38402	0.97094	0.68704
S	-2.73457	2.92071	1.31283

C	-0.03632	-1.83897	-5.11995	C	2.49566	-2.97981	3.95251
C	-1.42031	0.63855	-3.674	C	5.54513	-1.36332	1.52369
C	-3.48964	5.92819	1.31281	C	4.15616	0.99818	-0.92008
C	-1.61211	-1.26489	-2.20763	H	-0.47534	-0.65739	-1.66902
C	4.02644	2.1793	-0.3857	C	0.72232	-2.91399	-0.29102
C	4.58874	0.95861	0.01266	C	2.1735	0.10123	-2.02737
C	-1.85495	-2.81328	1.11387	C	-1.31042	2.84871	-1.10528
C	-0.95858	-0.6531	-3.26293	C	-0.34397	0.69184	1.74873
C	-5.5713	1.25365	-2.05459	C	5.85506	-0.49215	0.28973
C	-0.59518	-1.69294	2.81162	C	-1.07556	-2.93004	1.27669
C	2.78693	2.58824	0.09502	C	-4.65985	-1.17585	0.64968
C	-4.38233	1.13327	-1.07584	C	-7.30336	1.35145	-0.56003
C	2.02631	1.82963	1.0162	C	-0.23767	-3.41744	2.2703
C	3.81952	0.17388	0.86617	O	-1.58118	-2.26625	-0.92272
C	2.562	0.56179	1.34932	C	-1.30458	3.17108	0.23807
C	-4.79932	0.11292	0.00676	O	-1.47281	-0.14663	2.03382
C	-1.53552	5.54016	-0.22042	C	-3.31615	1.22721	1.0463
C	-4.14664	2.4607	-0.32689	C	-3.39961	-1.15532	1.22141
C	-1.74181	-2.41397	2.45738	C	3.75712	-1.35571	-0.87871
C	-2.68597	-0.63838	-1.56387	C	1.06501	3.8292	0.15978
C	-0.20269	3.5872	-1.8707	C	3.29448	-4.88494	2.52812
C	0.28118	-1.78005	0.52692	C	-7.654	-0.77801	0.69946
C	-1.57424	3.91815	-2.02959	C	1.10633	3.32453	-1.18904
C	2.23135	-2.3928	-0.71005	C	-1.51378	-0.9876	-1.58486
C	-0.2707	1.71113	1.86482	S	2.63252	2.91043	-2.13896
C	-1.30528	2.72742	2.29022	C	-6.72815	-0.04945	-0.29855
C	6.37518	-0.85147	0.09007	C	4.5665	-0.27351	-0.51138
C	5.97707	0.5239	-0.47354	C	6.45134	0.83974	0.77575
O	0.13944	-1.15365	-3.86734	C	1.31645	-5.20121	4.02527
S	-1.23667	-2.91324	-1.56059	S	1.59444	-2.70879	-1.86743
C	-2.85378	-2.7626	3.45918	C	-6.67305	-0.82916	-1.62934
H	1.75456	-3.37661	-0.81708	C	1.55213	-3.44689	0.71104
C	-2.52775	-2.26294	4.87626	C	2.98115	1.18795	-1.64454
S	2.25842	4.22126	-0.48483	C	1.65948	5.13783	2.28517
O	1.19714	-1.41629	-0.39719	C	-0.16122	3.70954	0.82417
O	0.90298	2.42315	1.44674	C	2.56881	-1.18087	-1.59562
S	1.90924	-0.6623	2.52406	C	-5.31394	0.00965	0.28846
C	5.97327	0.44119	-2.01237	C	3.36013	3.64554	1.32983
C	-3.0336	-4.29446	3.50535	C	-3.33717	3.30081	-2.30707
C	7.01297	1.56628	-0.00476	C	-0.10186	2.95701	-1.8385
C	-4.17544	-2.10485	3.00479	C	-2.67634	0.03344	1.46445
S	-2.57427	3.09749	-3.28939	C	-0.64849	-2.66517	-0.04141
H	-0.67908	-1.23138	-5.77731	O	-2.3829	2.33872	-1.77864
H	0.38076	5.62992	0.78293	H	-5.0544	2.15201	0.24685
H	-3.24661	5.11666	-1.49067	H	7.12274	-0.57223	-1.49177
H	-0.99965	6.38838	2.41823	H	6.50199	-2.16164	-0.99135

H	-0.47617	7.67113	1.2951	H	7.81701	-1.39055	-0.06741
H	-1.91298	7.90842	2.31547	H	1.81347	6.482	-0.05427
H	-3.44084	7.37691	-1.05445	H	3.09196	5.50693	-0.83854
H	-1.89609	8.22021	-0.7772	H	3.42525	6.34544	0.69014
H	-3.27794	8.49352	0.31812	H	3.01761	-2.24243	3.32344
C	1.33296	-2.04785	-5.72966	H	1.63335	-2.47764	4.41765
H	-0.53734	-2.80071	-4.91404	H	3.18418	-3.29455	4.7534
H	-4.18423	5.49706	0.57666	H	4.81751	-0.8637	2.18264
H	-3.17941	5.12464	1.99773	H	5.1278	-2.34192	1.24568
H	-4.04669	6.67734	1.89892	H	6.463	-1.54768	2.10346
H	4.53443	2.83088	-1.09904	H	4.75714	1.87589	-0.70323
H	-2.72369	-3.36817	0.75726	H	-0.42254	1.597	2.36089
H	-5.8258	0.26785	-2.47514	C	0.9457	-0.0458	2.03246
H	-5.35312	1.94497	-2.87407	H	-5.11225	-2.15429	0.48514
H	-6.4547	1.63363	-1.51536	H	-6.68909	1.92717	-1.26743
H	-0.43639	-1.34392	3.83155	H	-7.38879	1.93436	0.36968
H	4.18633	-0.79048	1.2037	H	-8.31092	1.26612	-0.99614
H	-5.09002	-0.86106	-0.4124	H	-0.6718	-3.56296	3.25891
H	-4.01021	-0.05104	0.75672	H	4.05116	-2.37335	-0.62649
H	-5.67963	0.51179	0.53199	H	3.93477	-4.17967	1.97757
H	-4.22439	3.31928	-0.99338	H	3.02271	-5.70447	1.84468
H	-4.90986	2.58599	0.45598	H	3.90544	-5.30526	3.34172
H	-3.16507	2.47339	0.16551	H	-7.29969	-1.79556	0.92271
H	-3.19167	-1.21026	-0.7969	H	-7.71271	-0.22529	1.65001
H	2.93349	-2.42125	0.13688	H	-8.67105	-0.86198	0.28495
C	2.87426	-1.96576	-2.00426	C	-2.14054	-1.1085	-2.95352
H	-0.01802	1.06665	2.71206	H	-2.0672	-0.26043	-0.9724
H	-0.63044	1.0929	1.02348	H	5.73442	1.40191	1.39488
H	6.455	-0.83978	1.18809	H	6.76091	1.48332	-0.06166
H	5.65639	-1.63609	-0.19111	H	7.34475	0.64566	1.38827
H	7.35719	-1.14374	-0.31183	H	0.90095	-6.03807	3.44185
H	-1.59513	-2.70095	5.26329	H	0.49268	-4.72849	4.5797
H	-2.43638	-1.16607	4.91379	H	2.01499	-5.61505	4.76962
H	-3.33647	-2.54855	5.56595	H	-6.25908	-1.84028	-1.49272
H	5.70625	1.40385	-2.47476	H	-6.03816	-0.30477	-2.36095
H	5.25203	-0.31124	-2.36015	H	-7.68166	-0.9321	-2.0601
H	6.96881	0.14978	-2.38431	H	2.57438	-3.68456	0.43161
H	-3.3289	-4.70493	2.52775	H	1.37266	4.34814	2.99618
H	-2.1033	-4.79261	3.81987	H	0.80608	5.81899	2.15257
H	-3.82193	-4.56037	4.22734	H	2.46972	5.71448	2.75479
H	6.7972	2.56625	-0.41192	H	-0.25294	4.00079	1.865
H	7.02016	1.64316	1.09383	H	2.9903	2.76633	1.88146
H	8.02287	1.27777	-0.33837	H	3.92783	3.3183	0.46036
H	-4.47336	-2.43191	1.99691	H	4.05073	4.18987	1.99368
H	-4.08946	-1.00672	2.99402	H	-2.78636	4.03946	-2.91492
H	-4.98958	-2.37467	3.69591	C	-4.33913	2.5289	-3.13296

O	-1.95172	3.32796	-4.61993	O	3.78511	3.67155	-1.63351
O	2.49133	5.17418	0.60755	O	2.35812	2.95869	-3.57394
O	1.57936	0.02383	3.78539	O	0.63694	-2.54612	-2.96354
O	-3.94867	3.59012	-3.13406	O	2.59439	-3.78523	-1.91965
O	2.93313	4.43315	-1.77826	O	-3.59618	-3.75988	1.02959
O	2.87041	-1.7782	2.55259	O	-2.8182	-2.76185	3.23474
O	-0.07569	-3.51033	-2.22877	O	-2.25916	3.03357	2.6989
O	-2.50714	-3.65392	-1.52842	O	-3.79385	3.82725	0.83418
O	-0.79456	1.10807	-4.74662	O	1.05525	0.30277	-2.75245
O	0.26066	2.71759	-2.76878	O	-0.18889	2.67596	-3.14342
H	-1.09794	2.04103	-4.92168	H	-1.05668	2.24891	-3.26114
C	1.37482	1.83925	-2.59818	C	1.18941	0.16189	-4.1792
H	1.30338	1.12579	-3.42655	H	0.18412	0.29554	-4.59344
H	1.2928	1.29223	-1.64927	H	1.54806	-0.84742	-4.4207
H	2.31523	2.39814	-2.65146	H	1.85555	0.94566	-4.56137
H	1.24614	-2.59863	-6.67895	H	-3.84752	1.99194	-3.95897
H	1.97336	-2.62843	-5.04919	H	-5.08731	3.21415	-3.55978
H	1.82	-1.08108	-5.93076	H	-4.85913	1.78898	-2.50829
H	-2.19855	2.20242	2.66339	H	1.7855	0.64164	1.84164
H	-1.60254	3.37486	1.45625	H	1.06746	-0.91683	1.3786
H	-0.90888	3.36174	3.09792	H	0.99781	-0.38264	3.0775
H	2.12331	-1.97482	-2.80463	H	-2.1478	-0.11588	-3.43055
H	3.69812	-2.64953	-2.26016	H	-3.1808	-1.46256	-2.87886
H	3.28231	-0.94896	-1.91195	H	-1.56445	-1.80562	-3.57456

6. References

(1) Marx, A.; Thiele, C. Orientational Properties of Poly-gamma-benzyl-L-glutamate: Influence of Molecular Weight and Solvent on Order Parameters of the Solute. *Chem. Eur. J.* **2009**, *15*, 254-260.

(2) Marx, A.; Schmidts, V.; Thiele, C. M. How different are diastereomorphous orientations of enantiomers in the liquid crystalline phases of PBLG and PBDG: a case study. *Magn. Reson. Chem.* **2009**, *47*, 734-740.

(3) Jeziorowski, S.; Thiele, C. Poly-gamma-*p*-biphenylmethyl-glutamate as Enantiodifferentiating Alignment Medium for NMR-Spectroscopy with Temperature Tunable Properties. *Chem. Eur. J.* **2018**, , 15631-15637.

(4) Thiele, C. M.; Bermel, W. Speeding up the measurement of one-bond scalar (^1J) and residual dipolar couplings (1D) by using non-uniform sampling (NUS). *J. Magn. Reson.* **2012**, *216*, 134-143.

(5) Thiele, C. M.; Schmidts, V.; Böttcher, B.; Louzao, I.; Berger, R.; Maliniak, A.; Stevensson, B. On the Treatment of Conformational Flexibility when Using Residual Dipolar Couplings for Structure Determination. *Angew. Chem. Int.* **2009**, *48*, 6708-6712.

(6) Frisch, M. J.; Trucks, G. W.; Schlegel, H. B.; Scuseria, G. E.; Robb, M. A.; Cheeseman, J. R.; Montgomery Jr., J. A.; Vreven, T.; Kudin, K. N.; Burant, J. C.; et al. Gaussian 03, Revision D.01. Gaussian, Inc., Wallingford CT, **2004**.

(7) Lee, C.; Yang, W.; Parr, R. G. Development of the Colle-Salvetti correlation-energy formula into a functional of the electron density. *Phys. Rev. B* **1988**, *37*, 785-789.

(8) Ditchfield, R.; Hehre, W. J.; Pople, J. A. Self-Consistent Molecular-Orbital Methods. IX. An Extended Gaussian-Type Basis for Molecular-Orbital Studies of Organic Molecules. *J. Chem. Phys.* **1971**, *54*, 724-728.

(9) Neese, F. Software update: the ORCA program system, version 4.0. *WIREs Computational Molecular Science* **2018**, *8*, e1327.

(10) Schäfer, A.; Horn, H.; Ahlrichs, R. Fully optimized contracted Gaussian basis sets for atoms Li to Kr. *J. Chem. Phys.* **1992**, *97*, 2571-2577.

(11) Grimme, S.; Ehrlich, S.; Goerigk, L. Effect of the damping function in dispersion corrected density functional theory. *J Comput. Chem.* **2011**, *32*, 1456-1465.

(12) Ochterski, J. W. Vibrational analysis in Gaussian. *help@ gaussian. com* **1999**.
